# The Role of Intrinsic Factors in Explaining Range Shifts of European Breeding Birds: A Meta‐Analysis

**DOI:** 10.1002/ece3.71308

**Published:** 2025-04-21

**Authors:** Femke E. M. Warmer, Wessel A. van Vliet, Pim van Hooft, Anouschka R. Hof

**Affiliations:** ^1^ Wildlife Ecology and Conservation Group Wageningen University Wageningen the Netherlands

**Keywords:** birds, climate change, functional traits, life history, meta‐analyses, species range shifts

## Abstract

Species are shifting their distribution ranges in response to climate and land‐use change. However, the observed range shift patterns are idiosyncratic in rate and direction. Species traits, such as ecological, life‐history and movement traits, may play an important role in determining range shifts by influencing a species' capacity to shift successfully. Whilst several studies investigate the role of different species traits in driving range shifts, they generally consider few traits and range shift types. Range shift types such as abundance shift and centroid shift are generally not taken into account. Drivers of range shifts may, however, differ per range shift type. We conducted a meta‐analysis to uncover the role of intrinsic factors (nine species functional traits and five spatial abundance characteristics) in explaining six contemporary range shift types (range size changes: expansion/contraction, relative change and rate of change; latitudinal shifts: abundance shift, centroid shift and range margin shift) in European breeding birds (*n* = 270). We found that the role of intrinsic factors in explaining contemporary range shifts in European breeding birds is indeed range shift type dependent. Natal dispersal distance and clutch size were, for instance, positively related to range size changes, while diet breadth and conservation status showed both negative and positive relationships depending on the range shift type. Acknowledging limitations regarding unevenness of data availability across the study region, the region of study was an important predictor for range size changes, suggesting a relative importance of local context and extrinsic drivers. Future trait‐based analyses of range shifts would benefit from accounting for intraspecific variation in functional traits across time and space, the inclusion of additional traits like phenological traits, exposure to environmental pressures, and competitive ability, and should be investigated across multiple scales and for multiple types of range shifts.

## Graphical abstract

We conducted a meta‐analysis on the role of intrinsic factors in explaining range shifts in European breeding birds and identified several intrinsic factors with significant effects. Our study further highlights a bias in range shift studies towards a handful of countries in Europe. Furthermore, future trait‐based analyses of range shifts would benefit from accounting for intraspecific variation in functional traits across time and space, and the inclusion of additional traits.

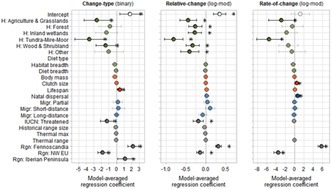

## 1 Introduction

Global human‐induced environmental changes, such as climate and land‐use change, are well known to cause changes in species distribution ranges (for example Chen et al. 2011; Parmesan 2006; Parmesan and Yohe 2003). Over the years, numerous studies have demonstrated that species from many taxa are shifting their geographic distribution ranges, generally polewards and to higher elevations, in response to climate and land‐use change (e.g., Brommer et al. 2012; Chen et al. 2011; Eglington and Pearce‐Higgins 2012; La Sorte and Thompson III 2007; Massimino et al. 2015; Parmesan and Yohe 2003; Howard et al. 2023). Despite the many documented poleward and elevational shifts, the observed range shift patterns seem to be idiosyncratic in rate and direction, with species showing great variation in shifts (Chen et al. 2011; Howard et al. 2023) that vary substantially across locations (Neate‐Clegg et al. 2021) and that may also be dependent on the season (Lehikoinen et al. 2021).

Understanding the dynamics and drivers (both extrinsic and intrinsic) involved with range shifts in species distributions is important for assessing species vulnerability to changing environments as well as aiding (future) conservation efforts (see for example Araújo et al. 2011; Wang et al. 2022). Thus, the observed heterogeneous range shift patterns limit our ability to accurately predict future changes in species distributions and to draw inferences from other studies. Small‐scale differences in climate effects or interactions with other drivers such as land‐use change or habitat availability play a role in the idiosyncrasy of responses (Chen et al. 2011; Eglington and Pearce‐Higgins 2012; Grenouillet and Comte 2014; Hovick et al. 2016; Li and Park 2020; Sirami et al. 2017; Tingley et al. 2012). Additionally, climatic tolerances of species may also affect the extent to which species will shift in response to climate change (Estrada et al. 2016; Jiguet et al. 2010).

Yet, range shifts are not solely the results of environmental or extrinsic drivers. A recent publication by Howard et al. (2023) for instance, revealed that changes in climate suitability poorly explained local colonisations and extinctions of bird species in Europe. Instead, they found that the distance to areas where birds were present, initial climate suitability, and, in case of colonisation, range size or, in case of extinction, the distance to the species' centre of gravity, were the strongest drivers of local colonisation and extinction events. Such findings illustrate the complexity of the drivers of distributional shifts. Besides these extrinsic and biogeographic factors, intrinsic factors (i.e., species traits including traits related to life history, morphology, phenology, physiology, behavior, movement, conservation status) could affect range and altitude shift patterns by influencing a species' capacity to shift its range (Couet et al. 2022; Estrada et al. 2016).

Over the last decade there has been more attention paid to species traits being possible predictors for range shifts (Angert et al. 2011; Buckley and Kingsolver 2012; Estrada et al. 2016; MacLean and Beissinger 2017; Pacifici et al. 2020). The capacity of a species to make a climate‐induced shift can rely on the ability of that species to move to and establish itself in new areas (Estrada et al. 2016). In this context, successful range shifts may firstly be influenced by a species' mobility and dispersal capacity, which can be determined by, for instance, natal dispersal ability and migration strategy (Bradshaw et al. 2014; Estrada et al. 2016). Although being migratory could be expected to increase movement ability, migratory species often show strong site fidelity and limited ability to expand their range due to constraints related to complex migration behaviors (Angert et al. 2011; Bensch 1999; Estrada et al. 2016). Second, a species has to be able to establish itself and persist in the new area. Traits associated with that ability are, for example, diet and habitat breadth, as well as reproductive strategy. Ecological generalists might be better at establishing themselves in new areas, as they are not bound to one habitat or diet type (Angert et al. 2011; Estrada et al. 2016). Species with a fast reproductive strategy will more rapidly establish a relatively large population, increasing the chance of avoiding small population effects (Auer and King 2014; Estrada et al. 2016). Species with a fast reproductive strategy are generally characterized by a small body mass, large number of offspring and a relatively short lifespan (Gaillard et al. 1989; Sæther 1987; Salguero‐Gómez et al. 2016).

Although some works find that species' traits are weak predictors of species' range shifts (Beissinger and Riddell 2021; Howard et al. 2023), others demonstrate that species traits can in fact be related to range shift patterns (e.g., Auer and King 2014; Estrada et al. 2015; Hof et al. 2017; Pacifici et al. 2020; Sunday et al. 2015). The generality of the results is however not straightforward, and traits seem to vary in their explanatory power (Angert et al. 2011; MacLean and Beissinger 2017). Additionally, there have been counterintuitive results for some traits. For example, increased diet breadth is expected to be positively correlated with range shifts, which has been supported by several studies (Angert et al. 2011; Pacifici et al. 2020) but rejected by others (Auer and King 2014). These mixed findings stress the idiosyncrasy of species' responses to climatic changes and the complexity of uncovering drivers of responses. Although there is no consensus on the generality of species traits as range shift predictors, various ecological and life‐history traits do seem to correlate with (the ability to) shift geographic distribution ranges, and there is compelling hypothetical support.

Getting a better understanding of range shift trends and related explanatory factors is beneficial for future species vulnerability assessments and directing conservation efforts. The identification of species traits related to range shifts will aid more comprehensive distribution predictions under future climate scenarios. Meta‐analysis approaches have been used to study the role of species traits in explaining species range shifts for a variety of taxa (Angert et al. 2011; MacLean and Beissinger 2017; Przeslawski et al. 2012; Sunday et al. 2015); however, only three focused specifically on birds (Angert et al. 2011; Yang et al. 2020; Neate‐Clegg et al. 2021) despite it being a well‐studied taxon (BirdLife International 2018). To date, the role of species traits in explaining range shifts of birds has been studied across the tropics and in North America, China and the United Kingdom (Angert et al. 2011; Auer and King 2014; Bradshaw et al. 2014; Yang et al. 2020; Neate‐Clegg et al. 2021). Another method to investigate the relationship between intrinsic factors and range shifts is to use data from species range atlases from two or more time periods. This method has been adopted by Howard et al. (2023). They used data from two breeding bird atlases to investigate drivers of local colonisation and extinction, considering climate and land‐use change as well as seven species traits. All these studies, as well as studies on other taxa, generally took a limited number of traits into account and have sparsely or not included potentially relevant traits like natal dispersal distance and thermal tolerance. Furthermore, the existing studies that try to uncover the role of different species traits in driving range shifts have not considered a large variety of range shift types (e.g., centroid shift, northern margin shift, see below). As different traits have been shown to drive, for example, local colonisation versus local extinction (Howard et al. 2023), the idiosyncrasy of responses may well be partly caused by the type of range shift considered. Here, we thus ask (1) how intrinsic species traits affect different types of range shifts in European breeding birds and (2) if drivers vary depending on the type of range shift. Specifically, we studied the role of a large number (*n* = 14) of intrinsic factors on six different range shift types (range size changes: expansion/contraction, relative change and rate of change; latitudinal shifts: abundance shift, centroid shift and range margin shift) in European breeding birds. We hypothesised that the effect of species traits varies among the different range shift types and that multiple traits play a role in explaining the variation in observed range shift patterns.

We used a meta‐analysis approach. Although data are inevitably available for more species when uncovering temporal trends from atlases compared to using meta‐analysis, there are other challenges to address with using atlas data. First, differences in survey effort between atlas periods can affect reporting rates, necessitating adjustments for accurate comparisons (Barrett et al. 2007). When combining data from different survey methods, it is vital to validate the comparability between datasets (Freeman et al. 2007). Although the two European Breeding Birds Atlases (EBBA1 and EBBA2) are very useful, there are some differences in data collection methods and observer efforts as well as some regions lacking data in EBBA1 (Herrando et al. 2013; Keller 2017). This may complicate comparability and present issues when analysing temporal trends in range shifts. Additionally, regional differences in population trends may exist, further complicating range shift analyses across large geographic areas (Freeman et al. 2007). This is corroborated by Neate‐Clegg et al. (2021), who found that responses of tropical montane bird communities were best predicted within a local or regional context. We thus chose to investigate the relationship between intrinsic factors and range shifts by means of a meta‐analysis of published literature. We do not necessarily suggest that a meta‐analysis approach may lead to better inferences than a breeding bird atlas approach. Instead, similarity between results using these different approaches increases the robustness of conclusions that are drawn.

## 2 Materials and Methods

### 2.1 Range Shift Data

Range shift data were extracted from literature. Scientific papers were identified from the online databases Web of Science and Scopus following a systematic review protocol (Page et al. 2021). The web application Rayyan was used for screening of titles and abstracts (Ouzzani et al. 2016). The following inclusion criteria were used in the screening process to assess the search results: (1) papers needed to study changes in species distribution ranges, (2) study subjects needed to be bird species with breeding ranges in Europe and (3) papers needed to study range shifts that have occurred between 1970 and the present day (i.e., no predicted future changes). The year 1970 was chosen as the baseline, as global changes attributed to human activity, such as global warming and land‐use intensification, have increased substantially from around that time onwards (Kastner et al. 2022; Knutson et al. 2017; Krausmann et al. 2013; Vose et al. 2012; Wuebbles et al. 2017). Finally, included studies reported on at minimum qualitative changes in the case of expansion/contraction and/or on quantitative changes for any other range shift types and had openly available data. See Appendix S1 for a full overview of the used search queries and the systematic review flow diagram. A final set of 41 papers (Appendix S2) was included, containing 1334 datapoints for 308 species.

**FIGURE 1:**
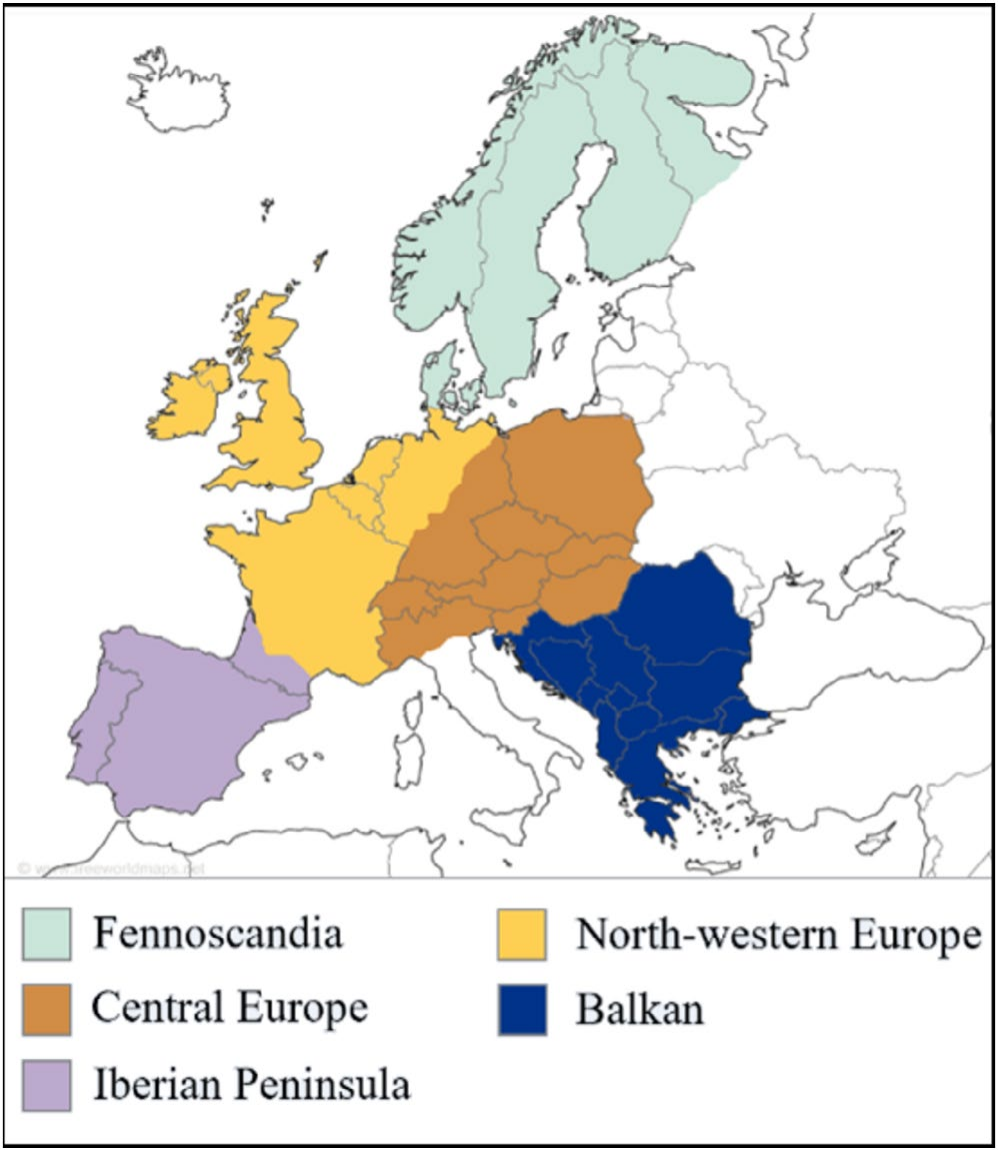
Map of the regions included in this study: Fennoscandia (light blue), North‐western Europe (yellow), Central Europe (brown), Balkan (dark blue) and Iberian Peninsula (lilac).

Range shift data were extracted either directly from Data S1 or from the pdf file with the use of webtools (Extracttable (ExtractTable 2021), Docsumo (Docsumo 2021) and WebPlotDigitizer (Rohatgi 2021)). When needed, magnitude values were transformed to standard metric units to allow for direct comparison. Data were assigned to a region based on the study area of the respective paper. Regions included in the dataset were Fennoscandia, North‐western Europe (NW EU), Central Europe (Central EU), Iberian Peninsula, Balkan, and Europe‐wide (for data that could not be divided into separate regions) (Figure 1). Several papers used the same source data, and additional filtering was applied to eliminate duplicate datapoints with identical source data per species per region. Furthermore, for three species in the United Kingdom ( *Emberiza cirlus* , *Caprimulgus europaeus* and *Lullula arborea* ) data from two sources were combined, as they represented studies from consecutive time periods. The included papers studied a variety of different range shift types, and range shift data were therefore categorised into six types: three related to range size changes (expansion/contraction, rate of change and relative change) and three related to latitudinal shifts (abundance shift, centroid shift and range margin shift).

### 2.2 Range Size Changes

This range shift type describes the changes in species range sizes, and data were expressed qualitatively as Change‐type (Expansion or Contraction; no‐shift cases were excluded from analysis). A no‐shift was assumed when no or minimal range difference was observed (i.e., reported range at time 2 = reported range at time 1, < 0.005% relative change, < 0.5 km^2^/year) and quantitatively as either Rate‐of‐change in km^2^/year or relative amount of change (Relative‐change, unitless). The latter was estimated using the formula by Böhning‐Gaese and Bauer (1996): Change = (range size at time 2 − range size at time 1)/((range size at time 2 + range size at time 1)/2). This formula makes it possible for change to be expressed symmetrically for expansion and contraction (respectively max. 2 or −2), instead of infinite positive change and a maximum of 100% negative change. As far as possible, data from each paper were included in the dataset in all three units (Change‐type: *n* = 679, Relative‐change: *n* = 581; Rate‐of‐change: *n* = 419), with negative values indicating range contraction and positive values indicating range expansion.

### 2.3 Abundance Shift

Two kinds of shifts were grouped into this range shift type: density shift and shift in centre of gravity. Two articles explicitly studied density shifts, where latitudinal values were weighted by species densities (Välimäki et al. 2016) or species densities were used for calculating an arithmetic centre of gravity (Lehikoinen and Virkkala 2016). Shifts were mainly weighted by species occurrence or count data for changes in centre of gravity. Shifts in density and centre of gravity do not necessarily also imply a shift in the latitudinal position of the range margin or the total distribution and are as such a separate range shift type. Abundance shifts were expressed in the unit km/year (*n* = 224). Negative values indicate a southward shift and positive values a northward shift.

### 2.4 Centroid Shift

This range shift type covers shifts in the latitudinal median of a species range and is not weighted by abundance or density. Centroid shift is expressed in the unit km/year (*n* = 227). Negative values indicate a southward shift and positive values indicate a northward shift.

### 2.5 Range Margin Shift

This constitutes changes in the latitudinal position of one of the margins of a species range. Papers reported on shifts for species with either their northern range margin (N‐margin) or their southern range margin (S‐margin) in the study area. Range margin shift was therefore included in two range shift types (N‐margin: *n* = 169, S‐margin: *n* = 35), with the unit km/year. Negative values indicate a southward shift and positive values a northward shift.

### 2.6 Predictors

Predictors included ecological factors (habitat breadth and type, diet breadth and type), life history traits (body mass, clutch size, and lifespan), movement‐related factors (natal dispersal distance, migration strategy), thermal tolerance indicators (thermal maximum, thermal range), spatial abundance indicators (threat status, historical range size (no log transformation) and northern limit in 1972–1995) and an extrinsic region indicator (except the latter, all are listed and explained in Table 1). The extrinsic region indicator included six regions based on where the source study was conducted (Fennoscandia, North‐western Europe (NW EU), Central Europe (Central EU), Iberian Peninsula, Balkan, and Europe‐wide for data that could not be divided into separate regions; Figure 1). Data on ecological factors, life history traits and migration strategy were collected from a dataset compiled by Storchová and Hořák (2018), which was extended with data from Hof et al. (2017), and online information by the International Union for Conservation of Nature (IUCN 2021), Birds of the World (Billerman et al. 2021), the British Trust for Ornithology (BTO 2021) and the AnAge database (Animal Ageing and Logevity Database; Tacutu et al. 2018). Data on natal dispersal distances were retrieved from a dataset by Barbet‐Massin et al. (2012). Following Jiguet et al. (2010), thermal maximum was determined by taking the mean spring/summer temperature for the 5% hottest grid cells in the historical European range (temperatures as mean monthly March–August temperatures for the years 1970–2000 as per the data from the WorldClim database (Fick and Hijmans 2017)). Thermal range was calculated as the difference between the thermal maximum and minimum (mean spring/summer temperature of the 5% coldest grid cells in the historical European range). As such, thermal variables relate to realised niches, not fundamental niches. Historical range variables were determined using data from the first European Breeding Bird Atlas (1972–1995; Hagemeijer et al. 2016; data accessed via GBIF.org). Historical European range size (km^2^) was manually calculated as the total area of occupied 50 × 50 km grid cells in the atlas data, and historical European northern limit as the average latitude of the 5% most northern grid cells the species was present in. Historical range variables and thermal variables were manually computed using R version 4.1.2 (R Core Team 2021). Mean values of functional traits were used in the analyses to identify interspecific differences.

**TABLE 1:** An overview of the species variables included in this study, with explanations.

Group	Species variable	Variable explanation	Units	Categories	Category definition
Ecological	Habitat breadth	# Habitat categories used	[−]	Forest, woodland, shrubland, savanna, tundra, grassland, mountain meadows, wetlands, desert, freshwater, marine, and rocks	
Ecological	Habitat type	Main habitat type	[−]	Habitat generalist	Species is associated with ≥ 2 of the categories below
				Agriculture & Grasslands	Species associated with agricultural and grassland habitats
				Forest	Boreal and temperate forest
				Inland wetlands	Species associated with inland wetlands, marshes, and reeds
				Tundra–Mire–Moor	For species that occupy tundra‐like habitats, tundra wetlands, or moor/heathland‐type habitats
				Wood & Shrubland	A species' habitat includes woodland, (temperate) shrubland or grassland/mountain meadows, or a combination of the three
				Fresh & Salt water	When a species is neither solely associated with inland wetlands or marine & coastal habitats
				Marine & Coastal	Species associated with marine and coastal habitats
				Open dry	A species inhabits dry open country, also including Mediterranean‐type shrubland
				Rocky areas	Species with rocky areas needed in breeding range
Ecological	Diet breadth	# Diet categories used	[−]	Folivore, frugivore, granivore, invertebrates, fish, other vertebrates, and carrion	
Ecological	Diet type	Main diet type	[−]	Herbivore	Diet of plant material and/or seeds
				Invertivore	Arthropods and other invertebrates
				Herbi‐invertivore	Species that switch between plant material and invertebrates depending on season
				Carnivore	Vertebrate diet other than fish and carrion
				Piscivore	Feeding on fish
				Omnicarnivore	Feeding on more than one type of animal matter (vertebrate, invertebrate, or carrion)
				Omnivore	Diet comprised of animal and plant matter
Life history	Body mass	Average adult body mass	[g]		
Life history	Clutch size	Average number of eggs per clutch	[−]		
Life history	Lifespan	Maximum lifespan (recorded in wild)	[years]		
Movement	Migration strategy	The type of migration	[−]	Sedentary	No migration
				Partial	Neither exclusively sedentary nor exclusively migratory
				Short‐distance	Migration within the western palearctic
				Long‐distance	Migration outside the western palearctic
Movement	Natal dispersal distance	Average displacement of first‐time breeding birds from their natal area	[km]		
Spatial abundance	IUCN status	The IUCN threat category for Europe	[−]	Least concern → Non‐threatened	IUCN status categories were grouped into Threatened and Non‐Threatened due to limited number of observations
				Near threatened → Threatened	
				Vulnerable → Threatened	
				Endangered → Threatened	
Spatial abundance	Historical range size	The size of a species total European range in 1972–1995	[km^2^]		
Spatial abundance	Historical northern limit	The latitude of the northern margin of a species European range in 1972–1995	[°]		
Thermal tolerance	Thermal maximum	The maximum mean spring/summer temperatures in the species European range	[°C]		
Thermal tolerance	Thermal range	The difference between maximum and minimum mean spring/summer temperatures in a species European range	[°C]		

Habitat breadth was expressed as the number of habitat categories a species occupies, based on the categories forest, woodland, shrubland, savanna, tundra, grassland, mountain meadows, wetlands, desert, freshwater, marine and rocks. The habitat type groups were: Habitat generalist, Agriculture & Grasslands, Forest, Inland wetlands, Tundra–Mire–Moor, Wood & Shrubland, Fresh & Salt water, Marine & Coastal, Open dry and Rocky areas. Diet breadth constitutes the number of diet categories (folivore, frugivore, granivore, invertebrates, fish, other vertebrates and carrion) a species uses and species were assigned to one of the following groups as main diet type: Herbivore, Invertivore, Herbi‐invertivore, Piscivore, Carnivore, Omnicarnivore and Omnivore. Migration strategy was included as a categorical variable with four levels: sedentary, partial, short‐distance and long‐distance. See Table 1 for category definitions and Appendix S6 (sheet: sps traits_explanations) for specific source information per predictors and categories.

Threat status was retrieved from the IUCN Red List website (IUCN 2021) and will from here on be referred to as IUCN status. The European IUCN status was used to assure uniformity across the different analyses, and because the approach of this study was to investigate range shifts across Europe. IUCN status categories included in this study were: Least concern, Near threatened, Vulnerable and Endangered. The categorical variables diet type, habitat type and IUCN status required merging of categories to adhere to the statistical requirements for some of the analyses; see Section 2.7 for more details.

### 2.7 Statistical Analysis

The study unit (one observation: *n*) in the current data represents a range shift identified in a source paper; this may result in more than one range shift per species per region per range shift type. Many observations came from relatively few papers and papers generally did not report similar data. Also, not every source provided each range shift type. These limitations necessitated a multi‐model approach because not all response variables were linear and random factors could not always be included. Thus, we used either linear models (lm's), linear mixed‐effect models (lmm's) or generalised linear mixed‐effect models (glmm's) to test for relationships between range shifts (response variable) on the one hand and species functional traits and spatial abundance characteristics (predictor variables) on the other (Appendix S3, Table S1). For all Range size change models, Species was included as a random factor to account for some species being present in more than one region. For simplicity and to allow for a more direct comparison between mixed and linear models, we decided not to include ‘study’ as a random factor. Besides, it had little effect on the full models (Appendix S4). Except for the extrinsic factor Region, standard errors differed by less than 15% and effect sizes by less than 35%, while, despite markedly increased standard errors for Region, only Fennoscandia in the Relative‐change model became non‐significant (*p* = 0.20, all other *p* < 0.033). Lmm's were used for Relative‐change and Rate‐of‐change, and a glmm was used for the binary response variable Change‐type. Lm's were used for Abundance, Centroid and N‐margin shifts, as these shift types either included data on only one region or the random factor species had zero variance in the mixed‐effect model. S‐margin shifts did not yield enough data for analysis. Model assumptions were checked for all model calls and log‐modulus transformation (John and Draper 1980) was applied to Relative‐change, Rate‐of‐change and Centroid shift to improve residual diagnostics (Appendix S5).

The continuous predictor variables were scaled to a mean of 0 and a standard deviation of 1 to ensure model convergence and to allow for comparison across traits and characteristics. Historical northern limit was not included in the Range size change model calls, since Range size change is assumed to be multidirectional. Regarding the categorical variable habitat type, all levels with fewer than 10 occurrences were grouped into the category Other. Habitat type was not included in the analyses for Abundance, Centroid and N‐margin shift due to lumping of half of the categories, rendering the variable less informative. The diet type categories Piscivore, Carnivore and Omnicarnivore were grouped into Omni‐carnivore for the Abundance, Centroid and N‐margin shift datasets, as one or more of these categories were not large enough (fewer than 10 occurrences) to be included as separate categories. Moreover, level Herbi‐invertivore was excluded from the analysis for N‐margin due to too few observations (*n* = 5) in this category. Only IUCN category Least concern yielded enough observations (*n* > 10) (for each shift type) to be included as a separate category; all other categories were grouped into Threatened. Additionally, regions Balkan and Europe‐wide had too few observations (*n* = 2 for both) and were excluded from the analyses. Multicollinearity was assessed with the use of generalised variance inflation factor scores (GVIF), and the variable thermal range was excluded due to GFIV above 5 for correlations with Abundance, Centroid, and N‐margin shift (in all other cases GFIV < 5).

With the dredge function in the *MuMIn* package (Barton 2020), the Akaike Information Criterion, adjusted for small sample sizes (AICc), was used to assess the best subset of models out of all possible models. All models with an AICc difference (∆_*i*_ = AICc_*i*_ − AICc_min_) of ≤ 2 compared to the model with the lowest AICc value are reported (∆_*i*_ ≤ 2, Appendix S3, Tables S2–S7). The number of candidate predictor variables in a model was limited to roughly one‐tenth of the number of observations in the response variable (Harrell et al. 1996). We lacked data on particular predictor variables for several species, so different subsets of data had to be used for each analysis (Relative‐change: *n* = 546, Rate‐of‐change: *n* = 394, Abundance: *n* = 213, Centroid: *n* = 211, N‐margin: *n* = 149). Additionally, Change‐type category No‐shift was not included in the model selection (*n* = 579). To account for model uncertainty, the regression coefficients *β* in all models with ∆_*i*_ ≤ 2 were averaged, with values of 0 when a variable was not included in a model (Burnham and Anderson 2004), which, in combination with the multi‐model approach, provided a robust sensitivity analysis. We acknowledge the fact that the effects and strength of coefficients can vary between models depending on the present covariates (Cade 2015). However, seeing the often large number of models with ∆_*i*_ ≤ 2 and the absence of a clear top‐ranked model, we opted to apply model averaging. An *α*‐level of 0.05 was maintained to assess the significance of the regression coefficients, and the regression coefficients for the log‐modulus transformed response variables were expressed as odds ratios (exp *β*) or percentages ((exp *β*) − 1) × 100. Significance of between‐factor level differences was determined using the *emmeans* package in R (Lenth 2022) and related *p* are reported after Bonferroni adjustment. The 95% confidence intervals of the predictor variables are shown in Appendix S3 (Tables S2–S7). The amount of variation explained by the models was estimated with marginal *R* ^2^ in the case of mixed models (Range size changes) and adjusted *R* ^2^ in the case of linear models (Abundance, Centroid and N‐margin shifts).

All data analyses were performed in R version 4.1.2 (R Core Team 2021) using the packages *lme4* version 1.1‐27.1 (Bates et al. 2015), *MuMIn* version 1.43.17 (Barton 2020), *DHARMa* version 0.4.5 (Hartig 2022), and *emmeans* version 1.7.2 (Lenth 2022). The packages *ggplot2* (Wickham 2016) and *gridExtra* version 2.3. (Auguie 2017) were used for data visualisation and *dplyr* version 1.0.7 (Wickham et al. 2021) was used during data manipulation. All raw data is available in Appendix S6. R scripts are stored in Dryad; Appendix S7–S11 Dataset DOI: 10.5061/dryad.wstqjq2z5.

## 3 Results

A total of 270 distinct species were included in the final dataset, across a total of 1151 individual range shifts (observations). Per region, this encompasses 183 species and 300 observations for Central Europe, 217 species and 672 observations for Fennoscandia, 41 species and 0 observations for the Iberian Peninsula, and finally 113 species and 138 observations for Northwestern Europe (regions Balkan and Europe‐wide did not yield enough observations to be included in the analysis).

**FIGURE 2:**
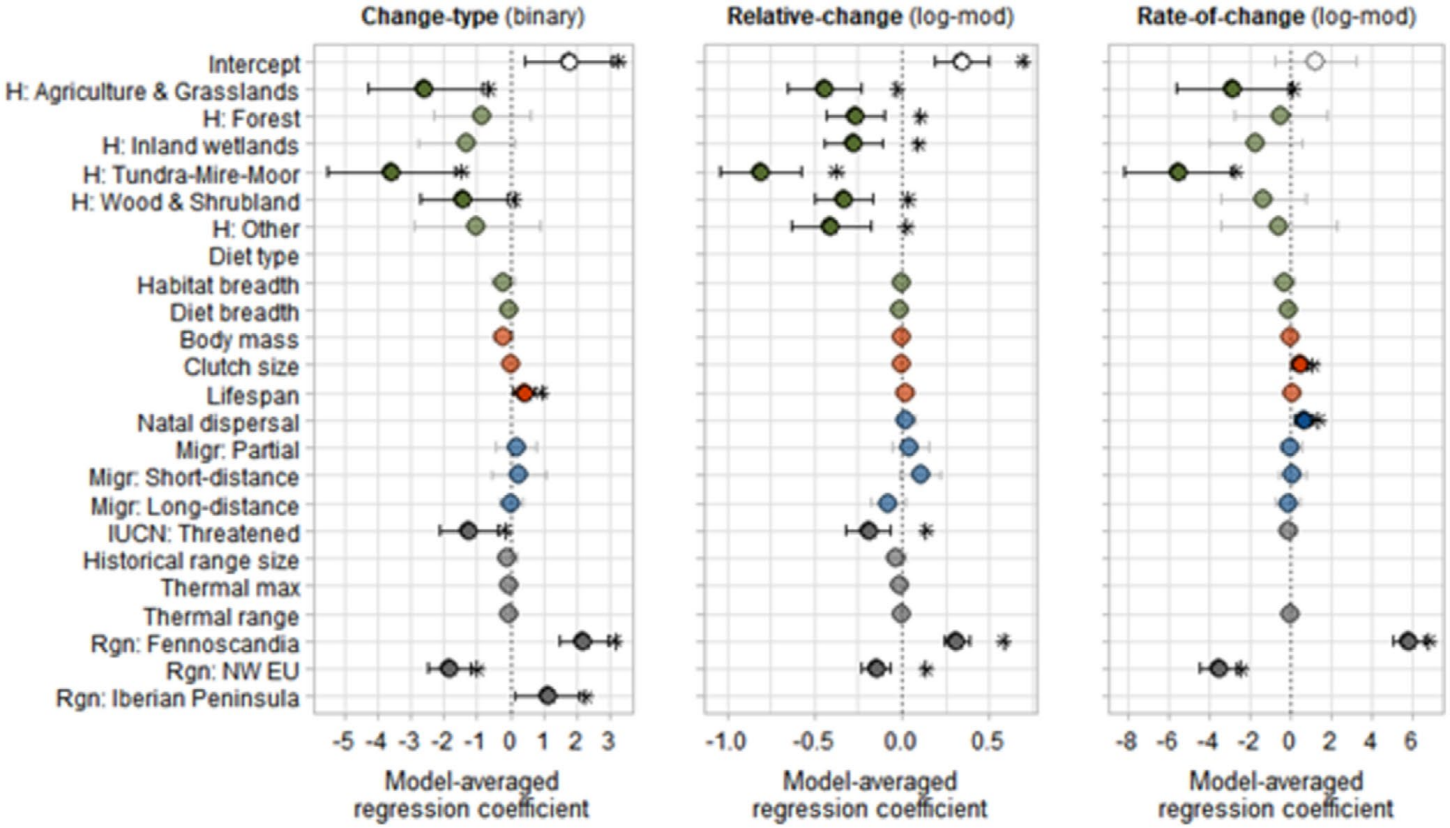
Plots of the model averaged regression coefficient *β* with confidence intervals (CI) of all predictor variables for the three range size change variables. Points with asterisks indicate a significant *β* (*p* < 0.05) with CI not including zero. Variables with ‘H:…’ refer to the habitat type categories with reference category ‘H: Habitat generalist’. ‘Migr:…’ indicates the categories for migration strategy with reference ‘Migr: Sedentary’ and ‘Rgn:…’ are the region categories with reference ‘Rgn: Central EU’. Colours indicate the predictor variable groups; green = Ecological, red = Life‐history, blue = Movement, grey = Other. Empty spaces indicate that a predictor was not included in the top‐ranked models. Detailed model output can be found in Appendix S3, Tables S2 and S4.

**FIGURE 3:**
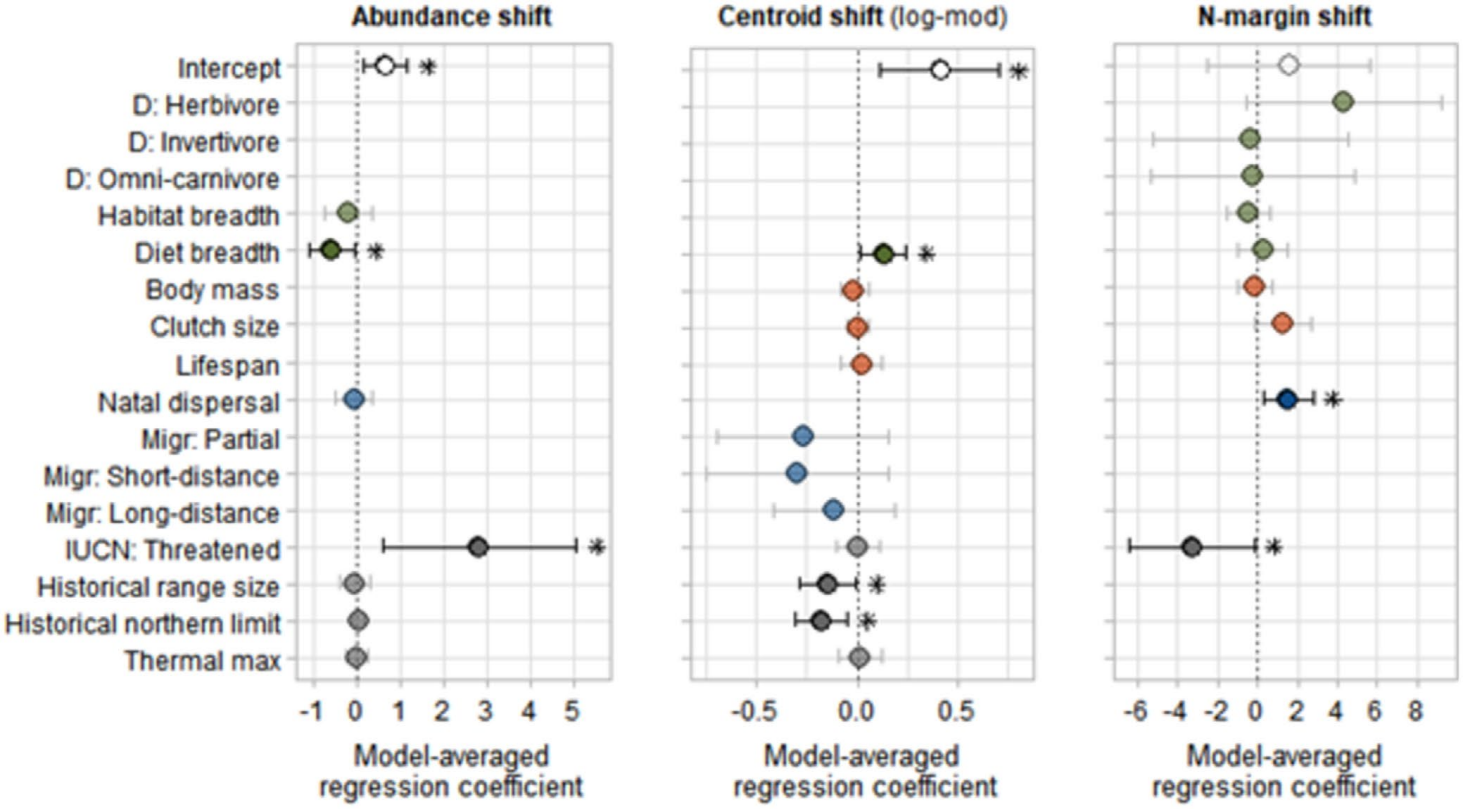
Plots of the model averaged regression coefficient *β* with confidence intervals (CI) of all predictor variables for the three latitudinal shift types: Abundance shift, centroid shift (with log‐modulus transformation) and N‐margin shift. Points with asterisks indicate a significant *β* (*p* < 0.05) with CI not including zero. Variables with ‘D:…’ are categories of predictor diet type with reference category ‘D: Omnivore’. ‘Migr:…’ indicates the categories for the predictor migration strategy with reference category ‘Migr: Sedentary’. Colours indicate the predictor variable groups; green = ecological, red = life history, blue = movement, grey = other. Empty spaces indicate that a predictor was not included in the top‐ranked models. Detailed model output can be found in Appendix S3, Tables S5–S7.

General result patterns of relationships between range shift types and species variables are summarised in Table 2 and visualised in Figures 2 and 3. The results are discussed per range shift type below.

**TABLE 2:** Summary table of the model results for the six range shift types and all species variables.

Variables	Range size change			Abun.	Centroid	N‐margin
	Type	Relative	Rate‐of			
Body mass						
Clutch size			+			
Lifespan	+					
Migratory strategy						
Natal dispersal			+			+
Habitat type	−	−	−			
Habitat breadth						
Diet type						
Diet breadth				−	+	
Thermal max						
Thermal range						
Historical range size					−	
Historical northern limit					−	
IUCN status	−	−		+		−
Region	+/−	+/−	+/−			

*Note:* Significant relationships between range shift types and species variables are indicated with + in green cells (positive relationship), − in blue cells (negative relationship), or +/− in yellow cells (either positive or negative depending on predictor category). Empty cells indicate that a variable either did not show a significant relationship or was not included in the top‐ranked models. Grey cells indicate that a variable was not included in the model call.

### 3.1 Range Size Change

The Change‐type dataset included data (*n* = 579) for four regions (Fennoscandia, NW EU, Central EU, and Iberian Peninsula) and 238 distinct species from 31 papers. Moreover, 84 out of the 136 species present in more than one region showed contrasting change types in different regions. Model selection revealed 24 models with ∆_*i*_ ≤ 2 and four intrinsic factors with significant influence on the probability of range expansion after model averaging (Figure 2; Appendix S3, Table S2). Within trait Habitat type, Agriculture & Grasslands, Tundra‐Mire‐Moor and Wood & Shrubland all had significant negative effects relative to category Habitat Generalist on the probability of expansion, with the respective odds ratios being 0.08, 0.03 and 0.25. IUCN status Threatened reduced the probability of range expansion by 72% compared to Least concern. Lifespan had a positive effect on the probability of expansion, with one standard deviation (SD) increase in lifespan (6.98 years) resulting in a 50% increase in the likelihood of expansion. Finally, the four regions differed in their effect on change type. Fennoscandia and Iberian Peninsula both were associated with a higher probability of expansion relative to Central EU (odds ratios of 9.00 and 3.00 respectively), while species in NW EU had a lower probability of expansion relative to Central EU (odds ratio = 0.16). Fennoscandia, Central EU, and NW EU were all significantly different from each other (*p* < 0.001), and the Iberian Peninsula was only significantly different from NW EU (*p* < 0.001; all other cases *p* > 0.05). Body mass was not significant after model averaging. The marginal *R* ^2^ of the top‐ranked models (∆_*i*_ ≤ 2) ranged between 0.401 and 0.434. Including the extrinsic factor region as random instead of fixed factor decreased the marginal *R* ^2^ of the top‐ranked model to 0.106.

Three regions (Fennoscandia, NW EU, Central EU) and 229 distinct species from 14 papers were included in the Relative‐change dataset (*n* = 546). The average Relative‐change was +0.12 (on a scale of −2 to +2, Appendix S12). Only three factors had significant effects on Relative‐change after model averaging (Figure 2; Appendix S3, Table S3). Species appointed as habitat generalists showed a larger relative amount of change in their range relative to species in other habitat groups, with the biggest difference being between categories Habitat generalist and Tundra‐Mire‐Moor (odds ratio = 0.44). Species classified as Threatened had a 17% lower Relative‐change than species classified as Least concern. Finally, the region categories differed significantly (*p* < 0.002), with the largest relative amount of change for species ranges in Fennoscandia (37% higher than Central EU) and the lowest for species ranges in NW EU (14% lower than Central EU). Although migration strategy was included in all top‐ranked models, there was no significant relationship with Relative‐change after model averaging. The marginal *R* ^2^ values of the top‐ranked models varied between 0.282 and 0.292. Including the extrinsic factor region as a random instead of fixed factor decreased the marginal *R* ^2^ of the top‐ranked model to 0.145.

Data on Rate‐of‐change (*n* = 394) included three regions (Fennoscandia, NW EU, and Central EU) and 223 species from 13 papers. Species ranges changed on average +457.9 km^2^/year (range: −3725 to +8281 km^2^/year, Appendix S12). The intrinsic factors were moderate predictors for the Rate‐of‐change, and four variables were present in all 10 top‐ranked models, which all showed significant effects after model averaging (Figure 2; Appendix S3, Table S4). Only the habitat categories Agriculture & Grasslands and Tundra‐Mire‐Moor showed significant relationships with Rate‐of‐change, with respective odds ratios of 0.06 and 0.004. Region also had a significant relationship with Rate‐of‐change. Compared to Central EU, Fennoscandia had significantly higher Rate‐of‐change (odds ratio = 346.19), and NW EU had a significant negative effect on Rate‐of‐change (odds ratio = 0.03). All three Region levels were significantly different (*p* < 0.001). Natal dispersal and clutch size both had a positive effect on Rate‐of‐change, with a 99% increase for every SD increase in the natal dispersal distance (17.4 km) and a 61% increase for every SD increase in clutch size (2.15 eggs). The marginal *R* ^2^ values of the top‐ranked models ranged between 0.527 and 0.537. Including the extrinsic factor region as a random instead of fixed factor decreased the marginal *R* ^2^ of the top‐ranked model to 0.059.

When comparing the output for the three Range size change variables, similar patterns are visible (Figure 2). Species categorised as Habitat generalists were more likely to expand their range relative to (some of) the other habitat types. Similarly, increased clutch size, lifespan, and natal dispersal distance increased the probability of expansion. Contrastingly, the probability of expansion was lower for species with IUCN status Threatened and for species living in NW EU compared to the other regions. Diet type was not included in the top‐ranked models for any of the Range size change variables.

### 3.2 Abundance—Centroid—N‐Margin Shift

Data on abundance shifts included 212 observations for Fennoscandia, but only one for NW EU, with 140 distinct species from four papers. Species abundances shifted on average +0.78 km/year (range: −11.5 to +11.7, Appendix S12). Seven intrinsic factors were included in the top‐ranked models, with two variables, diet breadth and IUCN status, present in all top‐ranked models (*w*_sum_ = 1.00), both with a significant effect on the rate of abundance shift after model averaging (Figure 3, Appendix S3, Table S5). Diet breadth was negatively related to the rate of abundance shift with a −0.6 km/year decrease for every SD increase (0.9 diet groups), while species with IUCN status Threatened had a +2.8 km/year higher rate of abundance shift compared to category Least concern. The adjusted *R* ^2^ value for the top‐ranked models ranged from 0.052 to 0.061 (Appendix S3, Table S5).

Centroid shifts were identified for 211 species and only included observations for Fennoscandia (*n* = 211; as source papers [2] studying Centroid shifts were only available for Fennoscandia). The mean rate of centroid shift was +0.56 km/year (range: −7.2 to +14.1 km/year, Appendix S12). The top‐ranked linear models had adjusted *R* ^2^ values ranging between 0.058 and 0.080 (Appendix S3, Table S6). Diet breadth, historical northern limit and historical range size were included in all top‐ranked models, and all had a significant effect on the rate of centroid shift after model averaging (Figure 3; Appendix S3, Table S6). For every SD increase in diet breadth (0.88 diet groups) the rate of centroid shift increased 14%. Both historical range values had a negative effect on the rate of centroid shift: a 16% decrease for every SD increase in historical range size (2.1 million km^2^) and a 14% decrease for every SD increase in historical northern limit (4.12° latitude). Migration categories were not significantly related to the rate of centroid shift after model averaging.

Data included in the analyses for N‐margin shift (*n* = 149) contained 121 species from five papers and were spread across two regions: Fennoscandia (*n* = 105) and NW EU (*n* = 44). The average rate of margin shift was +1.56 km/year (range: −17.1 to +20.83, Appendix S11). Model selection followed by averaging revealed four species variables with high relative importance (IUCN status, natal dispersal, diet type and clutch size; *w*_sum_ = 0.91–1.00), of which two had a significant effect on the rate of margin shift (Figure 3; Appendix S3, Table S7). IUCN status was significantly related to the rate of margin shift, with category Threatened having a 3.25 km/year lower rate of margin shift compared to category Least concern. Natal dispersal distance had a positive influence on the rate of margin shift, with a 1.45 km/year increase in margin shift rate for every SD increase in natal dispersal distance (16.6 km). The adjusted *R* ^2^ values for the top‐ranked models varied between 0.057 and 0.077 (Appendix S3, Table S7).

Some contrasting results become visible when comparing the output for Abundance, Centroid and N‐margin shift (Figure 3). The rate of shift for species with a larger diet breadth was reduced for Abundance shifts but increased for Centroid shifts. Similarly, species with IUCN status Threatened had a decreased N‐margin rate of shift but an increased Abundance rate of shift. Historical range variables were only related to the rate of shift for Centroid shifts, and natal dispersal distance only for N‐margin shifts.

## 4 Discussion

Here we investigated how intrinsic species traits affect six different types of range shifts in European breeding birds and if drivers, of which we included 14 intrinsic factors, varied depending on the type of range shift. We hypothesised that the effect of species traits varied among the different range shift types and that multiple traits played a role in explaining the variation in observed range shift patterns. In contrast to the most recent study investigating drivers of range shifts in European breeding birds we are aware of (Howard et al. 2023), we found several functional traits and spatial abundance characteristics to be important in explaining range shifts. Differences between our findings and those of Howard et al. (2023) may be explained by the difference in approach used: comparison between breeding bird atlases versus a meta‐analysis. The differences in the results between our meta‐analysis and the study by Howard et al. (2023) based on atlas data likely stem from the fact that both methods have advantages and drawbacks. Drawbacks of using bird atlases comprise challenges related to comparability between methods used to compile atlases and gaps in data for which inferences are generally made. Yet the big advantage is that large geographic regions and many species are covered. Drawbacks of a meta‐analysis approach include (1) that not as many species are covered, (2) that not all included studies use the same metrics which could influence the uniformity of the data due to different adopted methods and time periods and (3) that included studies are often at smaller spatial scales. The big advantage of a meta‐analysis is, however, that the small scale of said included studies may uncover the existence of species' responses in a local or regional context. Although similarity between results using these different approaches increases the robustness of conclusions that are drawn, differences stress the complexity of uncovering drivers. Drivers may be scale dependent and, as found here, range shift type specific.

We found that multiple different intrinsic traits related to the different types of range shift. For example, habitat type played a relatively large and significant role in all shift types related to range size changes; change type (expansion/contraction), relative change, and rate of change. Whilst diet breadth played a role in two out of three latitudinal shift types; abundance shifts and centroid shifts. Yet, threat status and natal dispersal played a role in shift types related to range size changes and in latitudinal shift types.

We found that species with a larger natal dispersal distance had a greater rate of expansion as well as a greater rate of N‐margin shift. Dispersal ability has generally been theorised as a mechanism driving differences in geographical range sizes between species (Lester et al. 2007), and it is often regarded as an important variable in more complex species distribution modelling (Franklin 2010; Miller and Holloway 2015; Zurell 2017). Interestingly, in the current study, natal dispersal distance did significantly influence Rate‐of‐change but had no significant relationship with Relative‐change or Change‐type, which could suggest that species with larger natal dispersal distances can expand their range at a higher rate but do not necessarily achieve more or less expansion. Likewise, Lester et al. (2007) found no universal positive relationship between dispersal ability and geographical range size for marine species, and the authors mention in the context of site colonisation that, in general, dispersal ability might have a greater impact on the rate of range expansion rather than on the eventual size of the range per se. Additionally, it may be that the amount of range change is more influenced by extrinsic factors, such as local environmental context. Bradshaw et al. (2014) found that higher natal dispersal distance was negatively related to the amount of range change for both contracting and expanding breeding birds in the United Kingdom, which may be related to a relatively high importance of extrinsic factors in explaining range size changes.

In our study, clutch size was positively associated with range expansion rate. A larger clutch size is an indicator of a fast reproduction, which can aid in the rapid establishment of a species in a new area (Angert et al. 2011; Estrada et al. 2016). This link with rapid establishment is reflected in our results, as similar to natal dispersal distance, clutch size was significantly related to Rate‐of‐change, but not with Relative‐change or Change‐type. This indicates that species with larger clutch sizes might be able to establish themselves faster in newly available areas but are not automatically also able to expand more. The findings from Howard et al. (2023) however do not corroborate this; clutch size was not significantly related to local colonisation in their study. Likewise, a study on waterbirds in southern Africa found no relationship between clutch size and whether a species had expanded or contracted its range (Okes et al. 2008). They however did not study the relationship with clutch size on Rate‐of‐change like we did.

Besides clutch size, other indicators of fast reproduction (and thus potential higher rates of shift) are a short lifespan and a small body mass (Estrada et al. 2016; Gaillard et al. 1989; Salguero‐Gómez et al. 2016). Surprisingly, species with longer lifespans were more likely to expand their range according to our results, yet body mass was not significantly related to any of the range shift types. It could be that lifespan and body mass are not strong indicators of reproductive strategy for the included species. One reason could be that waterbirds, which are increasingly overwintering in the more northern European regions (Pavón‐Jordán et al. 2015; Marchowski et al. 2017), could be expanding their ranges fast. However, the underlying mechanism for the positive relationship between lifespan and likelihood of expansion is unclear but may be related to long‐lived species having longer generation times, which may result in relatively slow microevolutionary responses to a changing environment (Chevin et al. 2010). These slow microevolutionary responses may hinder a species from reacting to their changing local environment, increasing the need to seek other suitable areas, especially if plastic responses are not sufficient (Gienapp et al. 2008).

Habitat type proved to be an important predictor for all three range size change types. Species associated with habitat type Tundra‐Mire‐Moor showed the strongest negative relationship with Range size change. Furthermore, for Relative‐change, all habitat types were significantly different from the group Habitat generalist, indicating that Habitat generalists were more likely to show positive, higher relative amounts of range change (i.e., to expand more). Ecological generalists are expected to shift their range at a higher rate, as they are not restricted to one type of habitat or diet (Angert et al. 2011; Estrada et al. 2016). Interestingly, habitat breadth did not show a significant relationship with any of the range shift types, which would indicate that habitat generalists in this sense were not particularly more likely to shift than specialists. Hence, type of habitat seems to be more important than number of habitats or habitat generalisation. This relative importance of the specific habitat type as a predictor for range change could indicate the importance of extrinsic factors. Habitat type Tundra‐Mire‐Moor was associated with the strongest negative effect for all three range size change types, and Moorland, for instance (which covers a larger area of north western EU) has been subject to degradation by, among others, increased pressure from sheep grazing, large‐scale afforestation, and acidic deposition (Holden et al. 2007; Thompson et al. 1995). Similarly, tundra ecosystems have been affected by climate change, resulting in, for example, advancing treelines and/or shrub vegetation (Callaghan et al. 2013; Molau 2010). This arctic greening was also found to be related to increased predation risk for ground nesting birds in tundra ecosystems (Ims et al. 2019). Moreover, Fraixedas et al. (2017) found substantial population declines in Fennoscandia for bird species associated with peatlands. Further, Elmhagen et al. (2015) studied range shifts for mammal and bird species in northern Sweden on the border between the boreal forest in the south and the tundra in the north and found that northern species showed significantly more contractions than southern species perhaps driven by a synergy of land use and climate change.

Threat status may indicate a species' sensitivity to pressures from extrinsic factors and thus local extinction and range contraction. We indeed found a significant negative relationship between threat status and Relative‐Change, Change‐type and N‐margin shift. Species with IUCN status threatened were more likely to experience a range contraction and had a higher rate of abundance shift than species classified as Least concern. This relationship is however biased by locality as the Abundance shift dataset only included data for Fennoscandia. The European IUCN status may not necessarily reflect a species' threat status in Fennoscandia. The Eurasian oystercatcher ( *Haematopus ostralegus* ) and Northern lapwing ( *Vanellus vanellus* ) are, for instance, indeed categorised as least concern on the 2019 Red List of Finnish species (Lehikoinen et al. 2019) as opposed to the classification of Vulnerable in Europe by the IUCN (BirdLife International 2021a, 2021b). However, for the other six Threatened species in the Abundance shift data, the two statuses did not differ. Vulnerable species might specifically rely on protected areas (Jackson and Gaston 2008), which in Finland are mainly located in the North (Virkkala and Rajasärkkä 2011). Virkkala and Rajasärkkä (2011) found that more northerly situated protected areas had a positive effect on the general population trends (either higher increase or lower decrease) of Finnish bird species. This positive effect of northern protected areas might explain a more northward Abundance shift for threatened species.

We further found that species with a larger diet breadth were negatively related to Abundance shifts and hence more likely to shift their abundance towards the south (or towards the north at a lower rate). However, at the same time, their range, irrespective of abundance, was more likely to shift towards the north (i.e., diet breadth was positively related to Centroid shifts). Species with a broad diet may be able to shift their ranges faster or may more easily adapt to local changes in environmental conditions. Previous studies identified both positive (Angert et al. 2011) and negative effects (Auer and King 2014) of diet breadth on northern range margin shifts in north American songbirds. Both the Abundance and Centroid shift datasets contained only data for Fennoscandia, while most species occur in many more areas across Europe. Essentially, Fennoscandia can be seen as the northern range edge for these species. As such, northward Centroid shifts in Fennoscandia could coincide with a northward shift of the centre of a species' entire European range and hence an increase of individuals in the south of Fennoscandia, since species abundances tend to be higher towards the centre of a species range (Brown 1984; Lawton 1993). This increase of individuals in the south could subsequently draw the centre of abundance in Fennoscandia southwards or slow down a northward abundance shift, while at the same time, the Centroid shift is positive/northwards. Thus, a larger diet breadth could be related to both a smaller/negative Abundance shift and a larger/positive Centroid shift within Fennoscandia. This theory is, however, dependent on species ranges extending beyond the southern edge of Fennoscandia and a disproportional increase of individuals in the south of Fennoscandia and the absence of ecological barriers between central Europe and Fennoscandia (Marjakangas et al. 2023).

The relative importance of extrinsic factors, such as changes in land‐use and climate change, in explaining range shifts is indicated by the significant regional differences. Species in Fennoscandia generally expanded their range whilst those in NW EU generally contracted their range. Species in Central EU generally contracted their range as well, but less so than those in NW EU. These patterns can be explained by different extrinsic factors influencing the various regions. Land‐use intensification in Europe over the past couple of decades has been highest in NW EU, while in Fennoscandia, intensification remained low and Central EU was characterised by both moderate land‐use intensification as well as extensification and agricultural land‐abandonment (Navarro and Pereira 2015, 11; Rasmussen and Weber 2013, 45–47). At the same time, NW EU is dominated by urban and suburban areas (Rasmussen and Weber 2013, 29). Furthermore, the effects of climate change have been and are projected to be greatest at higher latitudes (IPCC 2007; Kovats et al. 2014) and might therefore favour range expansions in Fennoscandia. However, this greater effect of climate change at high latitudes can also cause range contractions in cold climate specialists (Hof et al. 2017). The importance of local (regional) and extrinsic factors is further emphasised by the fact that, in the Change‐type dataset, two thirds of the species present in more than one region showed contrasting change types in different regions. Some examples are the Coal tit ( *Periparus ater* ) and Eurasian jay ( *Garrulus glandarius* ) with contraction in NW EU, but expansion in all other regions, the Hazel grouse (*Tetrates bonasia*) with expansion in Fennoscandia and contraction in Central EU, and the Long‐eared owl ( *Asio otus* ) with Expansion in Fennoscandia and contraction in Central and NW EU. However, direct comparison of all different range shift types in the current study is not entirely valid, as there were no data available for several of the regions for the Abundance, Centroid and N‐margin datasets. As a result, the current study also shows a bias of European range shift studies towards only a couple of countries within Europe. Data in Fennoscandia are dominated by studies from Finland (86% of the Fennoscandian data), NW EU is dominated by data from the British Isles (89%), and the Czech Republic is the main country contributing data for Central EU (59%). Moreover, the Balkan Peninsula was missing almost entirely from the complete dataset, encompassing only one datapoint.

We did not find significant relations between body mass and range shifts; adding to the variety of findings that have been found for the relationship between these factors (Angert et al. 2011; Bradshaw et al. 2014; MacLean and Beissinger 2017; Välimäki et al. 2016). The ultimate effect of body mass therefore remains unclear. It must however be noted that (sex‐specific) body mass can be a proxy for other life‐history and functional traits that we did not include in our study, such as survival rates (Cornioley et al. 2017). Likewise, migration strategy, thermal range and thermal maximum were not significantly related to any of the six range shift types; however, confidence intervals were large. We therefore cannot draw any firm conclusions. These factors previously all have shown positive, negative or, like in our case, no significant effects (Auer and King 2014; Hällfors et al. 2024).

Previous studies also found weak relationships between species traits and northern range margin shifts (Angert et al. 2011; MacLean and Beissinger 2017). This would suggest that species traits in general may not be strong predictors of (latitudinal) range shifts. However, we did not consider several aspects that could influence the predictive power of species traits. In most studies, average functional trait values per species are considered; however, considerable individual variation in functional trait values can occur across space and time (Beissinger and Riddell 2021). Individuals at the leading edge of a species range can experience different selection pressures, which can result in differences in functional traits related to dispersal ability, morphology, life‐history and behavior (Chuang and Peterson 2016), and local adaptation and phenotypic plasticity might induce intraspecific variation in functional trait values across time (Beissinger and Riddell 2021). Changes in phenology are a well‐observed example of plastic responses/adaptation to climate change, and multiple examples exist of advancements in the timing of avian breeding and migration (reviews by Charmantier and Gienapp 2014; Leech and Crick 2007; Visser and Both 2005). Accounting for intraspecific variation in functional trait values (across space and time) can thus be a valuable addition to trait‐based analyses. An additional extension of the current trait‐based research would be the incorporation of species traits related to, for instance, exposure to environmental pressures or community competition (reviewed by Beissinger and Riddell 2021). Traits related to exposure could include, for instance, fur or plumage attributes (such as colour and structure) that influence the experienced thermal load (Stuart‐Fox et al. 2017) or specific behaviors related to thermal regulation, such as activity patterns and habitat use/preferences (Long et al. 2005; Marshall et al. 2013; Murray and Smith 2012; Riddell et al. 2021). Both Beissinger and Riddell (2021) and Estrada et al. (2016) mentioned the possible importance of competitive ability; yet related traits can be difficult to quantify and are often context‐dependent, which makes it hard to incorporate this trait into trait‐based analyses of range shifts (Beissinger and Riddell 2021). Possible proxies for competitive ability could be local abundance (related to dominance) or brain size (related to innovation) (Estrada et al. 2016).

Regardless of the predictive power of various functional traits and spatial abundance characteristics, this study highlights that it may be difficult to generalise the effects of intrinsic factors as predictors across various types of range shifts, since the effects of the intrinsic factors differ among the six range shift types used in this study. It would be good practice for future range shift studies to consider several measures of range shifts. The current data indicate the importance of local context and regional differences in range changes, even within species. Focusing on country‐level data, like in the current study, can give a more detailed view of range shift dynamics and thus account for these local and regional differences.

## Author Contributions

**Femke E. M. Warmer:** formal analysis (lead), writing – original draft (lead), writing – review and editing (equal). **Wessel A. van Vliet:** formal analysis (supporting), writing – original draft (supporting), writing – review and editing (equal). **Pim van Hooft:** supervision (equal), writing – original draft (supporting), writing – review and editing (equal). **Anouschka R. Hof:** conceptualization (equal), supervision (equal), writing – original draft (supporting), writing – review and editing (equal).

## Conflicts of Interest

The authors declare no conflicts of interest.

## Supporting information


Data S1.

## Data Availability

The data used for this study was sourced from freely available publications. A list of publications used is included. Scripts for the analyses are stored in Dryad. Dataset DOI: 10.5061/dryad.wstqjq2z5.
