# Supplementary material for: The Role of Intrinsic Factors in Explaining Range Shifts of European Breeding Birds: A Meta‐Analysis
Source: Ecol Evol. 2025 Apr 21;15(4):e71308. doi: 10.1002/ece3.71308 (PMC12012262; doi:10.1002/ece3.71308)
Supplement: Supplementary file 1 — Data S1. [file ECE3-15-e71308-s001.zip › sm_0001-Supinfo.docx]

**Appendix 3 – Supplementary tables**

**Table S1** Model calls used in the model selection and multi-model inference process.

**Table S2** Output of the model selection for Change-type using generalized linear mixed models. All models with Δi≤2 are reported with **x** indicating when a predictor (with **x** for categories of that predictor) was included in a model. Per model, positive coefficients are shaded green and negative coefficients are shaded red. Akaike weights (wi, indicating the relative likelihood of a model (Burnham & Anderson, 2004)) and marginal R2 values are reported for each of the top-ranked models. For each predictor variable, the model averaged regression coefficient β and its confidence interval (CI) are reported, plus the exponent of β for odds ratios. The predictor specific w_sum_ is the sum of w_i_ for the models a predictor is included in and indicates the relative importance of a predictor variable. w_sum_ is 1 when a predictor is included in all top-ranked models. p-values are reported, and bold values mark significant β values with CI not including zero. Per model significances are illustrated with asterisks.

**Table S3** Output of the model selection for Relative-change using linear mixed models. Table components and variables are as explained for Table S2.

**Table S4** Output of the model selection for Rate-of-change using linear mixed models. Table components and variables are as explained for Table S2.

**Table S5** Output of the model selection for Abundance shift using linear models. Table components and variables are as explained for Table S2.

**Table S6** Output of the model selection for Centroid shift using linear models. Table components and variables are as explained for Table S2.

**Table S7** Output of the model selection for N-margin shift using linear models. Table components and variables are as explained for Table S2.

**Table S1**

|  |  | | |  |  |  |
| --- | --- | --- | --- | --- | --- | --- |
| **Response variables** | **Range size change** | | | **Abundance shift** | **Centroid shift** | **N-margin shift** |
|  | **Change-type^a^** | **Relative-change^b^** | **Rate-of-change^c^** |  |  |  |
| **Type of test** | glmm | lmm | lmm | lm | lm | lm |
| **n** | 211/368 | 546 | 394 | 213 | 211 | 149 |
|  |  |  |  |  |  |  |
| **Predictor variables** |  |  |  |  |  |  |
| Habitat type | **✓** | **✓** | **✓** | **x** | **x** | **x** |
| Habitat breadth | **✓** | **✓** | **✓** | **✓** | **✓** | **✓** |
| Diet type | **✓** | **✓** | **✓** | **✓** | **✓** | **✓** |
| Diet breadth | **✓** | **✓** | **✓** | **✓** | **✓** | **✓** |
| Body mass | **✓** | **✓** | **✓** | **✓** | **✓** | **✓** |
| Clutch size | **✓** | **✓** | **✓** | **✓** | **✓** | **✓** |
| Lifespan | **✓** | **✓** | **✓** | **✓** | **✓** | **✓** |
| Migration strategy | **✓** | **✓** | **✓** | **✓** | **✓** | **✓** |
| Natal dispersal | **✓** | **✓** | **✓** | **✓** | **✓** | **✓** |
| IUCN status | **✓** | **✓** | **✓** | **✓** | **✓** | **✓** |
| Thermal max | **✓** | **✓** | **✓** | **✓** | **✓** | **✓** |
| Thermal range | **✓** | **✓** | **✓** | **x** | **x** | **x** |
| Historical range size | **✓** | **✓** | **✓** | **✓** | **✓** | **✓** |
| Historical northern limit | **x** | **x** | **x** | **✓** | **✓** | **✓** |
| Region | **✓** | **✓** | **✓** | **x** | **x** | **✓** |
|  |  |  |  |  |  |  |
| **Random factor** | Species | Species | Species | x | x | x |
| **Notes** | Binomial distribution | Log-modulus transformed | Log-modulus transformed |  | Log modulus transformed |  |

a Range contraction or range expansion

b Relative-change = (range size at time 2 – range size at time 1)/((range size at time 2 + range size at time 1)/2)

c Rate of the change in range size (km2/year)

**Table S2**

|  |  |  |  |  |  |  |  |  |  |  |  |  |  |  |  |  |  |  |  |  |  |  |  |  |  |  | |  | |  | |  | |  | |  | |  |  |  |  |
| --- | --- | --- | --- | --- | --- | --- | --- | --- | --- | --- | --- | --- | --- | --- | --- | --- | --- | --- | --- | --- | --- | --- | --- | --- | --- | --- | --- | --- | --- | --- | --- | --- | --- | --- | --- | --- | --- | --- | --- | --- | --- |
|  |  | glmm - Model rank | | | | | | | | | | | | | | | | | | | | | | | | |  | |  | |  | |  | |  | | Model average | | | |  |
|  |  | 1 | 2 | 3 | 4 | 5 | 6 | 7 | 8 | 9 | 10 | 11 | 12 | 13 | 14 | 15 | 16 | 17 | 18 | 19 | 20 | 21 | 22 | 23 | 24 | 25 | | 26 | | 27 | | 28 | |  | | β | | 95% CI | w_sum_ | exp β | P |
| **Intercept** | | **x**** | **x**** | **x**** | **x**** | **x**** | **x**** | **x**** | **x**** | **x**** | **x**** | **x**** | **x**** | **x**** | **x**** | **x**** | **x**** | **x**** | **x**** | **x**** | **x*** | **x**** | **x**** | **x*** | **x***** | **x**** | | **x**** | | **x**** | | **x**** | |  | | **1.75** | | **0.46 to 3.03** | **-** | **-** | **0.008** |
| **Habitat type (ref: habitat generalist)** | | **x**** | **x**** | **x**** | **x**** | **x**** | **x**** | **x**** | **x**** | **x**** | **x**** | **x**** | **x**** | **x**** | **x**** | **x**** | **x**** | **x**** | **x**** | **x**** | **x**** | **x**** | **x**** | **x**** | **x**** | **x**** | | **x**** | | **x**** | | **x**** | |  | |  | |  | **1.00** |  |  |
| **Agriculture & Grasslands** |  | **x**** | **x**** | **x**** | **x***** | **x***** | **x**** | **x***** | **x**** | **x***** | **x**** | **x**** | **x**** | **x***** | **x***** | **x**** | **x***** | **x**** | **x***** | **x**** | **x**** | **x**** | **x***** | **x**** | **x***** | **x**** | | **x**** | | **x***** | | **x***** | |  | | **-2.57** | | **-4.28 to -0.87** |  | **0.08** | **0.003** |
| Forest |  | x | x | x | x | x | x | x | x | x | x | x | x | x* | x | x | x | x | x | x | x | x | x | x | x | x | | x | | x | | x | |  | | -0.83 | | -2.29 to 0.62 |  | 0.46 | 0.260 |
| Inland wetlands |  | x* | x | x | x* | x* | x* | x* | x | x** | x | x* | x* | x** | x* | x | x* | x | x* | x* | x | x | x* | x | x* | x* | | x* | | x** | | x* | |  | | -1.32 | | -2.76 to 0.12 |  | 0.28 | 0.072 |
| **Tundra-Mire-Moor** |  | **x***** | **x***** | **x***** | **x***** | **x***** | **x***** | **x***** | **x***** | **x***** | **x***** | **x***** | **x***** | **x***** | **x***** | **x***** | **x***** | **x***** | **x***** | **x***** | **x***** | **x***** | **x***** | **x***** | **x***** | **x***** | | **x***** | | **x***** | | **x***** | |  | | **-3.58** | | **-5.48 to -1.69** |  | **0.03** | **<0.001** |
| **Wood & Shrubland** |  | **x*** | **x*** | **x** | **x**** | **x**** | **x*** | **x*** | **x** | **x**** | **x*** | **x*** | **x*** | **x**** | **x*** | **x** | **x**** | **x** | **x**** | **x*** | **x*** | **x** | **x*** | **x*** | **x*** | **x*** | | **x*** | | **x**** | | **x*** | |  | | **-1.41** | | **-2.73 to -0.08** |  | **0.26** | **0.038** |
| Other |  | x | x | x | x | x | x | x | x | x | x | x | x | x | x | x | x | x | x | x | x | x | x | x | x | x | | x | | x | | x | |  | | -1.01 | | -2.89 to 0.87 |  | 0.39 | 0.294 |
| **IUCN status: 'Threatened'** | | **x**** | **x**** | **x**** | **x**** | **x**** | **x**** | **x**** | **x*** | **x**** | **x*** | **x**** | **x**** | **x**** | **x**** | **x*** | **x**** | **x**** | **x**** | **x**** | **x**** | **x**** | **x**** | **x**** | **x**** | **x**** | | **x**** | | **x**** | | **x**** | |  | | **-1.26** | | **-2.15 to -0.38** | **1.00** | **0.28** | **0.005** |
| **Lifespan** | | **x**** | **x**** | **x**** | **x*** | **x*** | **x**** | **x**** | **x*** | **x*** | **x*** | **x**** | **x**** | **x*** | **x**** | **x*** | **x*** | **x**** | **x**** | **x**** | **x*** | **x**** | **x** | **x** | **x**** | **x**** | | **x**** | | **x**** | | **x**** | |  | | **0.40** | | **0.08 to 0.73** | **1.00** | **1.50** | **0.014** |
| **Region (ref: Central EU)** | | **x**** | **x**** | **x**** | **x**** | **x**** | **x**** | **x**** | **x**** | **x**** | **x**** | **x**** | **x**** | **x**** | **x**** | **x**** | **x**** | **x**** | **x**** | **x**** | **x**** | **x**** | **x**** | **x**** | **x**** | **x**** | | **x***** | | **x**** | | **x**** | |  | |  | |  | **1.00** |  |  |
| **Fennoscandia** |  | **x***** | **x***** | **x***** | **x***** | **x***** | **x***** | **x***** | **x***** | **x***** | **x***** | **x***** | **x***** | **x***** | **x***** | **x***** | **x***** | **x***** | **x***** | **x***** | **x***** | **x***** | **x***** | **x***** | **x***** | **x***** | | **x***** | | **x***** | | **x***** | |  | | **2.20** | | **1.45 to 2.94** |  | **9.00** | **<0.001** |
| **Iberian Peninsula** |  | **x*** | **x*** | **x*** | **x*** | **x*** | **x*** | **x*** | **x*** | **x*** | **x*** | **x*** | **x*** | **x*** | **x*** | **x*** | **x*** | **x*** | **x*** | **x*** | **x*** | **x*** | **x*** | **x*** | **x*** | **x*** | | **x*** | | **x*** | | **x*** | |  | | **1.10** | | **0.15 to 2.05** |  | **3.02** | **0.024** |
| **North-western EU** |  | **x***** | **x***** | **x***** | **x***** | **x***** | **x***** | **x***** | **x***** | **x***** | **x***** | **x***** | **x***** | **x***** | **x***** | **x***** | **x***** | **x***** | **x***** | **x***** | **x***** | **x***** | **x***** | **x***** | **x***** | **x***** | | **x***** | | **x***** | | **x***** | |  | | **-1.86** | | **-2.49 to -1.22** |  | **0.16** | **<0.001** |
| Body mass | | x* | x | x | x | x* | x* | x | x | x* |  | x | x* | x* | x |  | x | x | x* | x* | x | x |  | x | x | x | | x* | | x* | | x | |  | | -0.22 | | -0.50 to 0.07 | 0.90 | 0.81 | 0.133 |
| Habitat breadth | | x | x |  | x | x | x | x |  | x | x | x | x | x | x |  | x |  | x | x |  |  | x |  | x | x | | x | | x | | x | |  | | -0.20 | | -0.55 to 0.14 | 0.76 | 0.82 | 0.253 |
| Historical range size | | x |  | x |  | x | x |  |  |  |  |  | x |  |  |  |  | x | x | x | x |  |  |  |  |  | | x | | x | |  | |  | | -0.09 | | -0.35 to 0.18 | 0.41 | 0.92 | 0.528 |
| Migration strategy (ref: sedentary) | |  |  |  | x | x |  |  |  | x |  |  |  | x |  |  | x |  | x |  | x |  | x | x |  |  | |  | | x | |  | |  | |  | |  | 0.34 |  |  |
| Partial |  |  |  |  | x | x |  |  |  | x |  |  |  | x |  |  | x |  | x |  | x |  | x | x |  |  | |  | | x | |  | |  | | 0.18 | | -0.45 to 0.81 |  | 1.17 | 0.575 |
| Short-distance |  |  |  |  | x* | x |  |  |  | x* |  |  |  | x* |  |  | x* |  | x |  | x |  | x* | x |  |  | |  | | x | |  | |  | | 0.25 | | -0.57 to 1.07 |  | 1.25 | 0.551 |
| Long-distance |  |  |  |  | x | x |  |  |  | x |  |  |  | x |  |  | x |  | x |  | x |  | x | x |  |  | |  | | x | |  | |  | | 0.02 | | -0.35 to 0.39 |  | 1.02 | 0.634 |
| Diet breadth | |  |  |  |  |  | x |  |  |  |  | x |  | x |  |  | x | x | x |  |  | x |  |  | x |  | | x | |  | | x | |  | | -0.05 | | -0.24 to 0.14 | 0.31 | 0.95 | 0.634 |
| Thermal Range | |  |  |  |  |  |  |  |  | x |  |  |  | x | x |  |  |  |  |  |  |  |  |  |  |  | |  | |  | | x | |  | | -0.02 | | -0.16 to 0.12 | 0.13 | 0.98 | 0.766 |
| Clutch size | |  |  |  |  |  |  |  |  |  |  |  | x |  |  |  |  |  |  |  |  |  |  |  |  | x | | x | | x | |  | |  | | 0.01 | | -0.10 to 0.13 | 0.11 | 1.01 | 0.813 |
| Thermal max | |  |  |  |  |  |  | x |  |  |  |  |  |  |  |  |  |  |  | x |  |  |  |  | x |  | |  | |  | |  | |  | | -0.02 | | -0.16 to 0.13 | 0.10 | 0.98 | 0.819 |
| Natal dispersal | |  |  |  |  |  |  |  |  |  |  |  |  |  |  |  |  |  |  |  |  |  |  |  |  |  | |  | |  | |  | |  | | - | | - | - | - | - |
| Diet type | |  |  |  |  |  |  |  |  |  |  |  |  |  |  |  |  |  |  |  |  |  |  |  |  |  | |  | |  | |  | |  | | - | | - | - | - | - |
|  |  |  |  |  |  |  |  |  |  |  |  |  |  |  |  |  |  |  |  |  |  |  |  |  |  |  | |  | |  | |  | |  | |  | |  |  |  |  |
| Δ_i_ | | 0.00 | 0.39 | 0.63 | 0.76 | 0.83 | 0.95 | 1.01 | 1.04 | 1.08 | 1.16 | 1.22 | 1.23 | 1.32 | 1.32 | 1.39 | 1.41 | 1.44 | 1.48 | 1.58 | 1.69 | 1.71 | 1.73 | 1.76 | 1.85 | 1.88 | | 1.92 | | 1.96 | | 1.96 | |  | |  | |  |  |  |  |
| w_i_ | | 0.067 | 0.055 | 0.049 | 0.046 | 0.044 | 0.042 | 0.040 | 0.040 | 0.039 | 0.037 | 0.036 | 0.036 | 0.035 | 0.034 | 0.033 | 0.033 | 0.033 | 0.032 | 0.030 | 0.029 | 0.028 | 0.028 | 0.028 | 0.026 | 0.026 | | 0.026 | | 0.025 | | 0.025 | |  | |  | |  |  |  |  |
| marginal R^2^ | | 0.421 | 0.413 | 0.414 | 0.424 | 0.430 | 0.424 | 0.419 | 0.407 | 0.430 | 0.407 | 0.419 | 0.422 | 0.434 | 0.417 | 0.401 | 0.428 | 0.418 | 0.434 | 0.424 | 0.424 | 0.411 | 0.418 | 0.418 | 0.421 | 0.415 | | 0.426 | | 0.432 | | 0.420 | |  | |  | |  |  |  |  |
|  |  |  |  |  |  |  |  |  |  |  |  |  |  |  |  |  |  |  |  |  |  |  |  |  |  |  | |  | |  | |  | |  | |  | |  |  |  |  |

Sign. codes: * p<0.05, ** p<0.01, *** p<0.001

**Table S3**

|  |  |  |  |  |  |  |  |  |  |  |  |  |  |  |  |  |  |  |  |  | |  | |  | |  |  |  |  |
| --- | --- | --- | --- | --- | --- | --- | --- | --- | --- | --- | --- | --- | --- | --- | --- | --- | --- | --- | --- | --- | --- | --- | --- | --- | --- | --- | --- | --- | --- |
|  |  | lmm - Model rank | | | | | | | | | | | | | | | | | | |  | |  | | Model average | | |  |  |
|  |  | 1 | 2 | 3 | 4 | 5 | 6 | 7 | 8 | 9 | 10 | 11 | 12 | 13 | 14 | 15 | 16 | 17 | 18 | 19 | |  | | β | | 95% CI | w_sum_ | Exp β | p |
| **Intercept** | | **x***** | **x***** | **x***** | **x***** | **x***** | **x***** | **x***** | **x***** | **x***** | **x***** | **x***** | **x***** | **x***** | **x***** | **x***** | **x***** | **x***** | **x***** | **x***** | |  | | **0.34** | | **0.19 to 0.49** | **-** | **1.40** | **<0.001** |
| **Habitat type (ref: Habitat generalist)** | | **x***** | **x***** | **x***** | **x***** | **x***** | **x***** | **x***** | **x***** | **x***** | **x***** | **x***** | **x***** | **x***** | **x***** | **x***** | **x***** | **x***** | **x***** | **x***** | |  | |  | |  | **1.00** |  |  |
| **Agriculture & Grasslands** |  | **x***** | **x***** | **x***** | **x***** | **x***** | **x***** | **x***** | **x***** | **x***** | **x***** | **x***** | **x***** | **x***** | **x***** | **x***** | **x***** | **x***** | **x***** | **x***** | |  | | **-0.44** | | **-0.66 to -0.23** |  | **0.64** | **<0.001** |
| **Forest** |  | **x**** | **x**** | **x**** | **x***** | **x**** | **x***** | **x***** | **x**** | **x**** | **x***** | **x***** | **x**** | **x**** | **x***** | **x***** | **x**** | **x**** | **x**** | **x**** | |  | | **-0.26** | | **-0.43 to -0.10** |  | **0.77** | **0.002** |
| **Inland wetlands** |  | **x***** | **x**** | **x**** | **x***** | **x**** | **x***** | **x***** | **x**** | **x**** | **x***** | **x**** | **x**** | **x***** | **x***** | **x***** | **x**** | **x**** | **x**** | **x**** | |  | | **-0.27** | | **-0.44 to -0.11** |  | **0.76** | **0.001** |
| **Tundra-Mire-Moor** |  | **x***** | **x***** | **x***** | **x***** | **x***** | **x***** | **x***** | **x***** | **x***** | **x***** | **x***** | **x***** | **x***** | **x***** | **x***** | **x***** | **x***** | **x***** | **x***** | |  | | **-0.81** | | **-1.05 to -0.58** |  | **0.44** | **<0.001** |
| **Wood & Shrubland** |  | **x***** | **x***** | **x***** | **x***** | **x***** | **x***** | **x***** | **x***** | **x***** | **x***** | **x***** | **x***** | **x***** | **x***** | **x***** | **x***** | **x***** | **x***** | **x***** | |  | | **-0.33** | | **-0.49 to -0.17** |  | **0.72** | **<0.001** |
| **Other** |  | **x***** | **x***** | **x***** | **x***** | **x***** | **x***** | **x***** | **x***** | **x**** | **x***** | **x***** | **x***** | **x**** | **x***** | **x***** | **x***** | **x***** | **x***** | **x***** | |  | | **-0.40** | | **-0.63 to -0.18** |  | **0.67** | **0.001** |
| **IUCN status: Threatened** | | **x**** | **x**** | **x**** | **x**** | **x**** | **x**** | **x**** | **x**** | **x**** | **x**** | **x**** | **x**** | **x**** | **x**** | **x**** | **x**** | **x**** | **x**** | **x**** | |  | | **-0.19** | | **-0.32 to -0.06** | **1.00** | **0.83** | **0.004** |
| **Region (ref: Central EU)** | | **x***** | **x***** | **x***** | **x***** | **x***** | **x***** | **x***** | **x***** | **x***** | **x***** | **x***** | **x***** | **x***** | **x***** | **x***** | **x***** | **x***** | **x***** | **x***** | |  | |  | |  | **1.00** |  |  |
| **Fennoscandia** |  | **x***** | **x***** | **x***** | **x***** | **x***** | **x***** | **x***** | **x***** | **x***** | **x***** | **x***** | **x***** | **x***** | **x***** | **x***** | **x***** | **x***** | **x***** | **x***** | |  | | **0.31** | | **0.24 to 0.38** |  | **1.37** | **<0.001** |
| **North-western EU** |  | **x***** | **x***** | **x***** | **x***** | **x***** | **x***** | **x***** | **x***** | **x***** | **x***** | **x***** | **x***** | **x***** | **x***** | **x***** | **x***** | **x***** | **x***** | **x***** | |  | | **-0.15** | | **-0.23 to -0.06** |  | **0.86** | **0.001** |
| Migration strategy (ref: sedentary) | | x | x | x | x | x | x | x | x | x | x | x | x | x | x | x | x | x | x | x | |  | |  | |  | 1.00 |  |  |
| Partial |  | x | x | x | x | x | x | x | x | x | x | x | x | x | x | x | x | x | x | x | |  | | 0.05 | | -0.05 to 0.15 |  | 1.05 | 0.351 |
| Short-distance |  | x* | x | x | x | x | x* | x | x | x* | x | x | x* | x* | x | x* | x | x | x | x | |  | | 0.11 | | -0.01 to 0.22 |  | 1.11 | 0.063 |
| Long-distance |  | x | x | x | x | x | x | x | x | x | x | x | x | x | x | x | x | x | x | x | |  | | -0.08 | | -0.18 to 0.02 |  | 0.93 | 0.127 |
| Historical range size | | x | x* | x* |  | x* | x | x | x* |  |  |  | x | x | x** |  | x | x* | x* | x* | |  | | -0.03 | | -0.08 to 0.02 | 0.75 | 0.97 | 0.217 |
| Natal dispersal | | x* |  |  | x* | x | x | x* | x | x* | x* | x* | x* | x* |  | x* |  |  |  |  | |  | | 0.03 | | -0.03 to 0.08 | 0.64 | 1.03 | 0.322 |
| Lifespan | |  | x* | x |  | x |  |  | x |  |  |  |  |  | x* |  | x* | x* | x* | x* | |  | | 0.02 | | -0.03 to 0.07 | 0.47 | 1.02 | 0.455 |
| Diet breadth | |  | x |  |  |  | x |  | x |  | x |  |  |  | x |  | x | x | x |  | |  | | -0.01 | | -0.04 to 0.02 | 0.40 | 0.99 | 0.552 |
| Thermal max | |  |  |  | x |  |  | x |  |  | x | x |  |  |  |  | x |  |  |  | |  | | -0.01 | | -0.04 to 0.03 | 0.25 | 0.99 | 0.667 |
| Habitat breadth | |  |  |  |  |  |  |  |  |  |  | x | x |  |  |  |  | x |  | x | |  | | -0.003 | | -0.03 to 0.02 | 0.17 | 1.00 | 0.781 |
| Clutch size | |  |  |  |  |  |  |  |  |  |  |  |  | x |  |  |  |  | x |  | |  | | 0.001 | | -0.01 to 0.01 | 0.08 | 1.00 | 0.866 |
| Body mass | |  |  |  |  |  |  |  |  |  |  |  |  |  | x |  |  |  |  |  | |  | | -0.001 | | -0.01 to 0.01 | 0.04 | 1.00 | 0.897 |
| Thermal range | |  |  |  |  |  |  |  |  |  |  |  |  |  |  | x |  |  |  |  | |  | | -0.001 | | -0.01 to 0.01 | 0.04 | 1.00 | 0.870 |
| Diet type | |  |  |  |  |  |  |  |  |  |  |  |  |  |  |  |  |  |  |  | |  | | - | | - | - | - | - |
|  |  |  |  |  |  |  |  |  |  |  |  |  |  |  |  |  |  |  |  |  | |  | |  | |  |  |  |  |
| Δ_i_ | | 0.00 | 0.09 | 0.38 | 0.60 | 0.85 | 1.08 | 1.31 | 1.33 | 1.37 | 1.41 | 1.44 | 1.46 | 1.53 | 1.59 | 1.66 | 1.68 | 1.69 | 1.80 | 1.89 | |  | |  | |  |  |  |  |
| w_i_ | | 0.093 | 0.089 | 0.077 | 0.069 | 0.061 | 0.054 | 0.048 | 0.048 | 0.047 | 0.046 | 0.045 | 0.045 | 0.043 | 0.042 | 0.041 | 0.040 | 0.040 | 0.038 | 0.036 | |  | |  | |  |  |  |  |
| marginal R^2^ | | 0.287 | 0.291 | 0.288 | 0.286 | 0.289 | 0.288 | 0.288 | 0.292 | 0.281 | 0.288 | 0.288 | 0.288 | 0.288 | 0.292 | 0.284 | 0.292 | 0.292 | 0.291 | 0.289 | |  | |  | |  |  |  |  |
|  |  |  |  |  |  |  |  |  |  |  |  |  |  |  |  |  |  |  |  |  | |  | |  | |  |  |  |  |

Sign. codes: * p<0.05, ** p<0.01, *** p<0.001

**Table S4**

|  | | | | | | | | | | | | | | | | | | |
| --- | --- | --- | --- | --- | --- | --- | --- | --- | --- | --- | --- | --- | --- | --- | --- | --- | --- | --- |
|  |  |  |  |  |  |  |  |  |  |  |  |  |  |  |  |  |  |  |
|  |  | lmm - Model rank | | | | | | | | | |  |  | Model average | | | |  |
|  |  | 1 | 2 | 3 | 4 | 5 | 6 | 7 | 8 | 9 | 10 | 11 |  | β | 95% CI | w_sum_ | exp β | p |
| Intercept | | **x** | **x** | **x** | **x** | **x** | **x** | **x** | **x** | **x** | **x** | **x** |  | 1.26 | -0.77 to 3.30 |  | 3.53 | 0.244 |
| Habitat type (ref: Habitat generalist) | | **x** | **x** | **x** | **x** | **x** | **x** | **x** | **x** | **x** | **x** | **x** |  |  |  | **1.00** |  |  |
| **Agriculture & Grasslands** |  | **x*** | **x*** | **x** | **x*** | **x** | **x** | **x*** | **x*** | **x*** | **x*** | **x*** |  | **-2.81** | **-5.57 to -0.04** |  | **0.06** | **0.047** |
| Forest |  | x | x | x | x | x | x | x | x | x | x | x |  | -0.46 | -2.76 to 1.83 |  | 0.63 | 0.693 |
| Inland wetlands |  | x | x | x | x | x | x | x | x | x | x | x |  | -1.71 | -4.02 to 0.59 |  | 0.18 | 0.145 |
| **Tundra-Mire-Moor** |  | **x***** | **x***** | **x***** | **x***** | **x***** | **x***** | **x***** | **x***** | **x***** | **x***** | **x***** |  | **-5.53** | **-8.15 to -2.91** |  | **0.004** | **<0.001** |
| Wood & Shrubland |  | x | x | x | x | x | x | x | x | x | x | x |  | -1.34 | -3.45 to 0.78 |  | 0.26 | 0.216 |
| Other |  | x | x | x | x | x | x | x | x | x | x | x |  | -0.54 | -3.36 to 2.29 |  | 0.59 | 0.710 |
| **Region (ref: Central EU)** | | **x***** | **x***** | **x***** | **x***** | **x***** | **x***** | **x***** | **x***** | **x***** | **x***** | **x***** |  |  |  | **1.00** |  |  |
| **Fennoscandia** |  | **x***** | **x***** | **x***** | **x***** | **x***** | **x***** | **x***** | **x***** | **x***** | **x***** | **x***** |  | **5.85** | **5.06 to 6.63** |  | **346.19** | **<0.001** |
| **North-western EU** |  | **x***** | **x***** | **x***** | **x***** | **x***** | **x***** | **x***** | **x***** | **x***** | **x***** | **x***** |  | **-3.54** | **-4.47 to -2.60** |  | **0.03** | **<0.001** |
| **Clutch size** | | **x*** | **x*** | **x** | **x*** | **x*** | **x*** | **x*** | **x*** | **x*** | **x*** | **x*** |  | **0.47** | **0.04 to 0.91** | **1.00** | **1.61** | **0.034** |
| **Natal dispersal** | | **x**** | **x**** | **x**** | **x***** | **x*** | **x**** | **x**** | **x*** | **x**** | **x**** | **x**** |  | **0.69** | **0.22 to 1.16** | **1.00** | **1.99** | **0.004** |
| Habitat breadth | | x | x |  | x |  |  | x | x | x | x | x |  | -0.29 | -0.85 to 0.26 | 0.71 | 0.75 | 0.302 |
| Lifespan | |  | x |  |  | x |  |  | x |  |  |  |  | 0.08 | -0.27 to 0.44 | 0.28 | 1.09 | 0.638 |
| Migration strategy (ref: Sedentary) | |  |  |  | x |  | x |  |  |  |  |  |  |  |  | 0.18 |  |  |
| Partial |  |  |  |  | x |  | x |  |  |  |  |  |  | 0.06 | -0.47 to 0.59 |  | 1.06 | 0.827 |
| Short-distance |  |  |  |  | x |  | x |  |  |  |  |  |  | 0.11 | -0.59 to 0.81 |  | 1.12 | 0.758 |
| Long-distance |  |  |  |  | x |  | x |  |  |  |  |  |  | -0.10 | -0.74 to 0.53 |  | 0.90 | 0.746 |
| Diet breadth | |  |  |  |  |  |  |  | x | x |  |  |  | -0.02 | -0.20 to 0.16 | 0.13 | 0.98 | 0.824 |
| Thermal range | |  |  |  |  |  |  | x |  |  |  |  |  | 0.01 | -0.12 to 0.15 | 0.07 | 1.01 | 0.862 |
| Body mass | |  |  |  |  |  |  |  |  |  | x |  |  | 0.01 | -0.09 to 0.11 | 0.06 | 1.01 | 0.914 |
| IUCN status: Threatened | |  |  |  |  |  |  |  |  |  |  | x |  | -0.02 | -0.40 to 0.36 | 0.06 | 0.98 | 0.915 |
| Thermal max | |  |  |  |  |  |  |  |  |  |  |  |  | - | - | - | - | - |
| Historical range size | |  |  |  |  |  |  |  |  |  |  |  |  | - | - | - | - | - |
| Diet type | |  |  |  |  |  |  |  |  |  |  |  |  | - | - | - | - | - |
| - |  |  |  |  |  |  |  |  |  |  |  |  |  |  |  |  |  |  |
| Δ_i_ | | 0.00 | 0.33 | 0.39 | 0.88 | 1.17 | 1.29 | 1.50 | 1.67 | 1.80 | 1.92 | 1.93 |  |  |  |  |  |  |
| w_i_ | | 0.154 | 0.131 | 0.127 | 0.099 | 0.086 | 0.081 | 0.073 | 0.067 | 0.063 | 0.059 | 0.059 |  |  |  |  |  |  |
| marginal R^2^ | | 0.530 | 0.533 | 0.527 | 0.537 | 0.529 | 0.534 | 0.531 | 0.534 | 0.531 | 0.531 | 0.531 |  |  |  |  |  |  |
|  |  |  |  |  |  |  |  |  |  |  |  |  |  |  |  |  |  |  |

Sign. codes: * p<0.05, ** p<0.01, *** p<0.001

**Table S5**

|  | |  | | | | | | | | | |  |  | |  |  |
| --- | --- | --- | --- | --- | --- | --- | --- | --- | --- | --- | --- | --- | --- | --- | --- | --- |
|  | | lm - Model rank | | | | | | | | | |  | Model average | |  |  |
|  |  | 1 | 2 | 3 | 4 | 5 | 6 | 7 | 8 | 9 | 10 |  | β | 95% CI | w_sum_ | p |
| **Intercept** | | **x*** | **x*** | **x*** | **x*** | **x*** | **x*** | **x*** | **x*** | **x*** | **x*** |  | **0.62** | **0.13 to 1.12** | - | **0.014** |
| **Diet breadth** | | **x*** | **x*** | **x*** | **x*** | **x*** | **x*** | **x*** | **x*** | **x*** | **x*** |  | **-0.60** | **-1.10 to -0.09** | **1.00** | **0.020** |
| **IUCN status: Threatened** | | **x*** | **x*** | **x**** | **x**** | **x*** | **x*** | **x**** | **x*** | **x*** | **x*** |  | **2.80** | **0.58 to 5.02** | **1.00** | **0.013** |
| Habitat breadth | | x |  | x |  | x | x |  |  |  | x |  | -0.22 | -0.76 to 0.32 | 0.55 | 0.422 |
| Natal dispersal | |  |  | x | x |  |  | x |  |  |  |  | -0.09 | -0.49 to 0.32 | 0.28 | 0.665 |
| Historical range size | |  |  |  |  | x |  | x | x |  |  |  | -0.06 | -0.40 to 0.28 | 0.25 | 0.710 |
| Thermal max | |  |  |  |  |  | x |  |  | x |  |  | -0.03 | -0.29 to 0.22 | 0.16 | 0.792 |
| Historical northern limit | |  |  |  |  |  |  |  |  |  | x |  | 0.01 | -0.14 to 0.15 | 0.07 | 0.917 |
| Body mass | |  |  |  |  |  |  |  |  |  |  |  | - | - | - | - |
| Clutch size | |  |  |  |  |  |  |  |  |  |  |  | - | - | - | - |
| Lifespan | |  |  |  |  |  |  |  |  |  |  |  | - | - | - | - |
| Migration strategy | |  |  |  |  |  |  |  |  |  |  |  | - | - | - | - |
| Diet type | |  |  |  |  |  |  |  |  |  |  |  | - | - | - | - |
|  |  |  |  |  |  |  |  |  |  |  |  |  |  |  |  |  |
|  | Δ_i_ | 0.00 | 0.81 | 1.11 | 1.63 | 1.65 | 1.68 | 1.71 | 1.80 | 1.81 | 1.94 |  |  |  |  |  |
|  | w_i_ | 0.193 | 0.129 | 0.110 | 0.085 | 0.085 | 0.084 | 0.082 | 0.079 | 0.078 | 0.073 |  |  |  |  |  |
|  | adj. R^2^ | 0.061 | 0.052 | 0.061 | 0.053 | 0.058 | 0.058 | 0.058 | 0.053 | 0.053 | 0.057 |  |  |  |  |  |
|  |  |  |  |  |  |  |  |  |  |  |  |  |  |  |  |  |

Sign. codes: * p<0.05, ** p<0.01, *** p<0.001

|  | | |  |  |  |  |  |  |  |  |  |  |  |  |  |  |  |  |  |
| --- | --- | --- | --- | --- | --- | --- | --- | --- | --- | --- | --- | --- | --- | --- | --- | --- | --- | --- | --- |
|  | | | lm - Model rank | | | | | | | | | | |  | Model average | |  |  |  |
|  |  | | 1 | 2 | 3 | 4 | 5 | 6 | 7 | 8 | 9 | 10 | 11 |  | β | 95% CI | w_sum_ | P | expβ |
| **Intercept** | | | **x***** | **x***** | **x***** | **x***** | **x***** | **x***** | **x***** | **x***** | **x***** | **x***** | **x***** |  | **0.41** | **0.12 to 0.71** | - | **0.006** | **1.51** |
| **Diet breadth** | | | **x*** | **x*** | **x*** | **x*** | **x*** | **x*** | **x*** | **x*** | **x*** | **x**** | **x*** |  | **0.13** | **0.01 to 0.24** | **1.00** | **0.027** | **1.14** |
| **Historical northern limit** | | | **x**** | **x**** | **x**** | **x** | **x**** | **x**** | **x**** | **x**** | **x**** | **x**** | **x**** |  | **-0.18** | **-0.31 to -0.05** | **1.00** | **0.007** | **0.84** |
| **Historical range size** | | | **x*** | **x** | **x*** | **x*** | **x*** | **x*** | **x*** | **x** | **x*** | **x*** | **x*** |  | **-0.15** | **-0.29 to -0.01** | **1.00** | **0.040** | **0.86** |
| Migration strategy (ref: Sedentary) | | | x |  | x | x | x | x | x |  | x | x |  |  |  |  | 0.74 |  |  |
|  | Partial |  | **x*** |  | **x*** | **x*** | **x*** | **x*** | **x*** |  | **x*** | **x*** |  |  | -0.27 | -0.70 to 0.16 |  | 0.219 | 0.76 |
|  | Short-distance |  | **x*** |  | **x*** | **x*** | **x*** | **x*** | **x*** |  | **x*** | **x*** |  |  | -0.30 | -0.75 to 0.16 |  | 0.203 | 0.74 |
|  | Long-distance |  | x |  | x | x | x | x | x |  | x | x |  |  | -0.12 | -0.42 to 0.19 |  | 0.463 | 0.89 |
| Lifespan | | |  |  | x | x |  |  | x |  |  |  |  |  | 0.03 | -0.08 to 0.13 | 0.28 | 0.625 | 1.03 |
| Body mass | | |  |  |  | x |  | x |  | x |  |  |  |  | -0.01 | -0.09 to 0.06 | 0.22 | 0.725 | 0.99 |
| Thermal max | | |  |  |  |  | x |  |  |  |  |  | x |  | 0.01 | -0.09 to 0.12 | 0.16 | 0.796 | 1.01 |
| Clutch size | | |  |  |  |  |  |  | x |  |  | x |  |  | 0.01 | -0.05 to 0.06 | 0.13 | 0.823 | 1.01 |
| IUCN status: Threatened | | |  |  |  |  |  |  |  |  | x |  |  |  | 0.01 | -0.10 to 0.12 | 0.07 | 0.888 | 1.01 |
| Natal dispersal | | |  |  |  |  |  |  |  |  |  |  |  |  | - | - | - | - | -  -  - |
| Habitat breadth | | |  |  |  |  |  |  |  |  |  |  |  |  | - | - | - | - | -  - |
| Diet type | | |  |  |  |  |  |  |  |  |  |  |  |  | - | - | - | - | - |
|  |  | |  |  |  |  |  |  |  |  |  |  |  |  |  |  |  |  |  |
|  | Δ_i_ | | 0.00 | 0.50 | 0.52 | 1.14 | 1.34 | 1.69 | 1.70 | 1.74 | 1.77 | 1.90 | 1.93 |  |  |  |  |  |  |
|  | w_i_ | | 0.164 | 0.128 | 0.127 | 0.093 | 0.084 | 0.071 | 0.070 | 0.069 | 0.068 | 0.063 | 0.063 |  |  |  |  |  |  |
|  | adj. R^2^ | | 0.076 | 0.059 | 0.078 | 0.080 | 0.075 | 0.073 | 0.078 | 0.058 | 0.073 | 0.072 | 0.057 |  |  |  |  |  |  |
|  |  | |  |  |  |  |  |  |  |  |  |  |  |  |  |  |  |  |  |

**Table S6**

Sign. codes: * p<0.05, ** p<0.01, *** p<0.001

**Table S7**

|  |  |  |  |  |  |  |  |  |  |  |  |  |  |  |
| --- | --- | --- | --- | --- | --- | --- | --- | --- | --- | --- | --- | --- | --- | --- |
|  |  |  | lm - Model rank | | | | | | |  | Model average | |  |  |
|  |  |  | 1 | 2 | 3 | 4 | 5 | 6 | 7 |  | β | 95% CI | w_sum_ | p |
|  | Intercept | | **x** | **x** | **x** | **x** | **x** | **x** | **x*** |  | 1.58 | -2.49 to 5.62 | - | 0.437 |
|  | **IUCN status: Threatened** | | **x*** | **x*** | **x*** | **x** | **x** | **x*** | **x*** |  | **-3.25** | **-6.30 to -0.19** | **1.00** | **0.038** |
|  | **Natal dispersal** | | **x*** | **x*** | **x*** | **x*** | **x*** | **x*** | **x*** |  | **1.45** | **0.26 to 2.65** | **1.00** | **0.017** |
|  | Diet type (ref: Omnivore) | | x | x | x | x | x | x | x |  |  |  | 1.00 |  |
|  | Herbivore |  | x | **x*** | **x*** | **x** | **x*** | x | x |  | 4.23 | -0.57 to 9.03 |  | 0.084 |
|  | Invertivore |  | x | x | x | x | x | x | x |  | -0.42 | -5.14 to 4.30 |  | 0.861 |
|  | Omni-carnivore |  | x | x | x | x | x | x | x |  | -0.27 | -5.31 to 4.76 |  | 0.915 |
|  | Clutch size | | x* | x* | x* | x* | x* | x* |  |  | 1.25 | -0.18 to 2.52 | 0.91 | 0.090 |
|  | Habitat breadth | | x |  | x |  |  | x | x |  | -0.43 | -1.47 to 0.62 | 0.56 | 0.424 |
|  | Diet breadth | |  |  | x | x |  |  |  |  | 0.25 | -0.98 to 1.47 | 0.25 | 0.694 |
|  | Body mass | |  |  |  |  | x | x |  |  | -0.14 | -0.96 to 0.67 | 0.22 | 0.729 |
|  | Lifespan | |  |  |  |  |  |  |  |  | - | - | - | - |
|  | Migration strategy | |  |  |  |  |  |  |  |  | - | - | - | - |
|  | Thermal max | |  |  |  |  |  |  |  |  | - | - | - | - |
|  | Historical range size | |  |  |  |  |  |  |  |  | - | - | - | - |
|  | Historical northern limit | |  |  |  |  |  |  |  |  | - | - | - | - |
|  | Region | |  |  |  |  |  |  |  |  | - | - | - | - |
|  |  |  |  |  |  |  |  |  |  |  |  |  |  |  |
|  | Δ_i_ | | 0.00 | 0.08 | 0.095 | 1.28 | 1.31 | 1.38 | 1.78 |  |  |  |  |  |
|  | w_i_ | | 0.220 | 0.212 | 0.137 | 0.116 | 0.115 | 0.110 | 0.090 |  |  |  |  |  |
|  | adj. R^2^ | | 0.076 | 0.068 | 0.077 | 0.068 | 0.068 | 0.075 | 0.057 |  |  |  |  |  |
|  |  |  |  |  |  |  |  |  |  |  |  |  |  |  |
|  |  |  |  |  |  |  |  |  |  |  |  |  |  |  |

Sign. codes: * p<0.05, ** p<0.01, *** p<0.001

**Appendix 4 – Comparison between full models**

Comparison between full models (lowest AICc values) including both study and species as random factor and those including only species as random factor.

###### Relative change (D): both study (authors) and species (eng_name) included as random factors

Linear mixed model fit by maximum likelihood . t-tests use Satterthwaite's method ['lmerModLmerTest']

Formula: (sign(D) * log(abs(D) + 1)) ~ IUCN + H_type + D_type + H_breadth_sum +

D_breadth_sum + migration + body_mass + clutch_size + lifespan +

natal_dispersal + thermal_max + thermal_range + hist_range + region + (1 | eng_name) + (1 | authors)

Data: D_glmm

AIC BIC logLik deviance df.resid

422.7 555.7 -180.3 360.7 509

Scaled residuals:

Min 1Q Median 3Q Max

-3.4438 -0.4382 0.0017 0.4248 3.5132

Random effects:

Groups Name Variance Std.Dev.

eng_name (Intercept) 0.01299 0.1140

authors (Intercept) 0.02211 0.1487

Residual 0.09878 0.3143

Number of obs: 540, groups: eng_name, 226; authors, 14

Fixed effects:

Estimate Std. Error df t value Pr(>|t|)

(Intercept) 0.475412 0.134906 30.233639 3.524 0.001375 **

IUCNThreatened -0.241266 0.065586 183.731030 -3.679 0.000308 ***

H_typeAgriculture_Grasslands -0.496723 0.119776 131.937395 -4.147 5.99e-05 ***

H_typeForest -0.385462 0.101487 147.476401 -3.798 0.000213 ***

H_typeInland wetlands -0.356610 0.099827 149.912628 -3.572 0.000476 ***

H_typeTundraMireMoor -0.903473 0.134045 220.106237 -6.740 1.37e-10 ***

H_typeWood_Shrubland -0.392817 0.093572 152.016127 -4.198 4.57e-05 ***

H_typeOther -0.454154 0.137580 200.820139 -3.301 0.001140 **

D_typeHerbi-invertivore -0.124121 0.098888 123.327166 -1.255 0.211793

D_typeHerbivore 0.024020 0.073091 122.800855 0.329 0.742997

D_typeInvertivore -0.081253 0.058727 128.104349 -1.384 0.168902

D_typeOmnicarnivore 0.044611 0.108686 167.513200 0.410 0.681992

D_typeOmnivore -0.005567 0.125499 117.316275 -0.044 0.964691

D_typePiscivore -0.102579 0.115480 177.069082 -0.888 0.375593

H_breadth_sum -0.028052 0.022715 128.501917 -1.235 0.219100

D_breadth_sum -0.051693 0.030868 134.144652 -1.675 0.096342 .

migrationMixed 0.060559 0.049368 97.702406 1.227 0.222890

migrationShort-distance 0.102782 0.058375 144.910279 1.761 0.080394 .

migrationLong-distance -0.078930 0.050482 112.688860 -1.564 0.120736

body_mass -0.030368 0.018569 267.161343 -1.635 0.103142

clutch_size 0.022373 0.022061 133.140009 1.014 0.312358

lifespan 0.029038 0.026584 140.039054 1.092 0.276570

natal_dispersal 0.041523 0.024696 138.283014 1.681 0.094948 .

thermal_max -0.017026 0.025503 264.527850 -0.668 0.504966

thermal_range -0.008181 0.025238 197.383687 -0.324 0.746179

hist_range -0.045073 0.029780 182.536368 -1.514 0.131871

regionFennoscandia 0.228669 0.159041 6.378558 1.438 0.197683

regionNorthwestern Europe -0.323066 0.124024 7.959547 -2.605 0.031513 *

---

R2m R2c

[1,] 0.3125525 0.4927934

##### Relative change (D): only species (eng_name) included as random factor

Linear mixed model fit by maximum likelihood . t-tests use Satterthwaite's method ['lmerModLmerTest']

Formula: (sign(D) * log(abs(D) + 1)) ~ IUCN + H_type + D_type + H_breadth_sum +

D_breadth_sum + migration + body_mass + clutch_size + lifespan +

natal_dispersal + thermal_max + thermal_range + hist_range + region + (1 | eng_name)

Data: D_glmm

AIC BIC logLik deviance df.resid

434.1 562.8 -187.0 374.1 510

Scaled residuals:

Min 1Q Median 3Q Max

-3.3064 -0.4902 -0.0017 0.4889 3.3302

Random effects:

Groups Name Variance Std.Dev.

eng_name (Intercept) 0.01368 0.1170

Residual 0.10476 0.3237

Number of obs: 540, groups: eng_name, 226

Fixed effects:

Estimate Std. Error df t value Pr(>|t|)

(Intercept) 4.748e-01 1.067e-01 1.519e+02 4.449 1.66e-05 ***

IUCNThreatened -2.085e-01 6.713e-02 1.892e+02 -3.106 0.002192 **

H_typeAgriculture_Grasslands -4.985e-01 1.231e-01 1.390e+02 -4.051 8.45e-05 ***

H_typeForest -3.679e-01 1.041e-01 1.542e+02 -3.533 0.000542 ***

H_typeInland wetlands -3.382e-01 1.023e-01 1.565e+02 -3.306 0.001174 **

H_typeTundraMireMoor -9.117e-01 1.355e-01 2.245e+02 -6.731 1.39e-10 ***

H_typeWood_Shrubland -3.991e-01 9.617e-02 1.599e+02 -4.150 5.39e-05 ***

H_typeOther -4.764e-01 1.414e-01 2.105e+02 -3.370 0.000894 ***

D_typeHerbi-invertivore -1.229e-01 1.011e-01 1.290e+02 -1.216 0.226207

D_typeHerbivore 1.763e-02 7.504e-02 1.282e+02 0.235 0.814673

D_typeInvertivore -8.672e-02 6.016e-02 1.332e+02 -1.442 0.151753

D_typeOmnicarnivore 5.856e-02 1.116e-01 1.745e+02 0.525 0.600300

D_typeOmnivore -3.805e-02 1.287e-01 1.218e+02 -0.296 0.767962

D_typePiscivore -8.533e-02 1.186e-01 1.860e+02 -0.720 0.472701

H_breadth_sum -2.523e-02 2.328e-02 1.338e+02 -1.084 0.280355

D_breadth_sum -4.747e-02 3.165e-02 1.406e+02 -1.500 0.135830

migrationMixed 5.205e-02 5.064e-02 1.021e+02 1.028 0.306499

migrationShort-distance 8.546e-02 5.962e-02 1.500e+02 1.433 0.153807

migrationLong-distance -8.544e-02 5.182e-02 1.180e+02 -1.649 0.101857

body_mass -2.831e-02 1.904e-02 2.765e+02 -1.487 0.138152

clutch_size 2.335e-02 2.263e-02 1.387e+02 1.032 0.303914

lifespan 3.214e-02 2.721e-02 1.468e+02 1.181 0.239354

natal_dispersal 3.376e-02 2.518e-02 1.430e+02 1.341 0.182146

thermal_max -2.781e-02 2.485e-02 2.780e+02 -1.119 0.264045

thermal_range 2.463e-04 2.552e-02 2.049e+02 0.010 0.992310

hist_range -2.590e-02 2.963e-02 1.864e+02 -0.874 0.383139

regionFennoscandia 3.118e-01 3.655e-02 3.624e+02 8.532 3.98e-16 ***

regionNorthwestern Europe -1.477e-01 4.241e-02 3.367e+02 -3.482 0.000564 ***

---

R2m R2c

[1,] 0.3102611 0.3899435

##### Rate-of-change (km2/year):both study (authors) and species (eng_name) included as random factors

Linear mixed model fit by maximum likelihood . t-tests use Satterthwaite's method ['lmerModLmerTest']

Formula: (sign(km2.year) * log(abs(km2.year) + 1)) ~ H_type + D_type +

H_breadth_sum + D_breadth_sum + migration + IUCN + body_mass + clutch_size + lifespan + natal_dispersal + thermal_max +

thermal_range + hist_range + region + (1 | eng_name) + (1 | authors)

Data: km2_glmm

AIC BIC logLik deviance df.resid

2129.0 2251.9 -1033.5 2067.0 358

Scaled residuals:

Min 1Q Median 3Q Max

-3.6973 -0.4717 0.0391 0.4748 3.2641

Random effects:

Groups Name Variance Std.Dev.

eng_name (Intercept) 0.9595 0.9795

authors (Intercept) 0.8443 0.9189

Residual 10.7025 3.2715

Number of obs: 389, groups: eng_name, 220; authors, 13

Fixed effects:

Estimate Std. Error df t value Pr(>|t|)

(Intercept) 2.17791 1.41808 33.93869 1.536 0.133854

H_typeAgriculture_Grasslands -2.81504 1.38628 212.87321 -2.031 0.043535 *

H_typeForest -1.10051 1.18267 239.39914 -0.931 0.353036

H_typeInland wetlands -2.23421 1.16439 239.99777 -1.919 0.056198 .

H_typeTundraMireMoor -5.78856 1.49355 295.05142 -3.876 0.000131 ***

H_typeWood_Shrubland -1.60171 1.10035 245.44487 -1.456 0.146771

H_typeOther -1.28969 1.61275 253.17785 -0.800 0.424644

D_typeHerbi-invertivore -0.23650 1.10672 182.82452 -0.214 0.831020

D_typeHerbivore 0.20381 0.82858 184.62186 0.246 0.805971

D_typeInvertivore -0.39280 0.66987 198.10463 -0.586 0.558288

D_typeOmnicarnivore 1.73419 1.24859 258.83954 1.389 0.166051

D_typeOmnivore 1.27454 1.40773 177.93074 0.905 0.366485

D_typePiscivore 0.07299 1.35620 236.51275 0.054 0.957124

H_breadth_sum -0.44430 0.25077 199.51033 -1.772 0.077962 .

D_breadth_sum -0.61983 0.34417 205.13548 -1.801 0.073179 .

migrationMixed 0.32167 0.55956 152.19347 0.575 0.566237

migrationShort-distance 0.53853 0.67831 220.63805 0.794 0.428085

migrationLong-distance -0.61851 0.57940 181.96023 -1.068 0.287160

IUCNThreatened -0.73456 0.73397 257.68506 -1.001 0.317861

body_mass -0.09327 0.21102 358.48304 -0.442 0.658753

clutch_size 0.42247 0.25188 202.66815 1.677 0.095031 .

lifespan 0.06991 0.30545 219.24697 0.229 0.819185

natal_dispersal 0.68353 0.28185 209.81322 2.425 0.016148 *

thermal_max -0.10254 0.30717 293.60867 -0.334 0.738742

thermal_range 0.17058 0.30737 296.29504 0.555 0.579347

hist_range -0.03659 0.35780 263.56035 -0.102 0.918633

regionFennoscandia 4.87689 1.19099 2.66844 4.095 0.032840 *

regionNorthwestern Europe -4.53887 1.01643 3.74979 -4.466 0.012861 *

---

R2m R2c

[1,] 0.516228 0.5860024

##### Rate-of-change (km2/year): only species (eng_name) included as random factor

Linear mixed model fit by maximum likelihood . t-tests use Satterthwaite's method ['lmerModLmerTest']

Formula: (sign(km2.year) * log(abs(km2.year) + 1)) ~ H_type + D_type +

H_breadth_sum + D_breadth_sum + migration + IUCN + body_mass + clutch_size + lifespan + natal_dispersal + thermal_max +

thermal_range + hist_range + region + (1 | eng_name)

Data: km2_glmm

AIC BIC logLik deviance df.resid

2128.7 2247.6 -1034.4 2068.7 359

Scaled residuals:

Min 1Q Median 3Q Max

-3.6424 -0.4791 0.0280 0.4645 3.3592

Random effects:

Groups Name Variance Std.Dev.

eng_name (Intercept) 1.009 1.004

Residual 10.982 3.314

Number of obs: 389, groups: eng_name, 220

Fixed effects:

Estimate Std. Error df t value Pr(>|t|)

(Intercept) 1.74993 1.24336 248.08345 1.407 0.160554

H_typeAgriculture_Grasslands -2.91499 1.40556 219.31859 -2.074 0.039257 *

H_typeForest -1.00966 1.19748 244.89550 -0.843 0.399968

H_typeInland wetlands -2.21855 1.17833 245.31814 -1.883 0.060913 .

H_typeTundraMireMoor -5.82742 1.49945 294.43334 -3.886 0.000126 ***

H_typeWood_Shrubland -1.62615 1.11547 252.76381 -1.458 0.146133

H_typeOther -1.33564 1.63314 259.33986 -0.818 0.414200

D_typeHerbi-invertivore -0.28229 1.11958 186.11769 -0.252 0.801211

D_typeHerbivore 0.12030 0.83974 188.99489 0.143 0.886241

D_typeInvertivore -0.36930 0.67753 201.03421 -0.545 0.586311

D_typeOmnicarnivore 1.83693 1.26422 263.86443 1.453 0.147409

D_typeOmnivore 0.99687 1.42459 180.52207 0.700 0.484979

D_typePiscivore 0.09580 1.37473 243.04911 0.070 0.944500

H_breadth_sum -0.43785 0.25383 202.82501 -1.725 0.086056 .

D_breadth_sum -0.55664 0.34819 209.87040 -1.599 0.111406

migrationMixed 0.31065 0.56699 155.18243 0.548 0.584558

migrationShort-distance 0.52638 0.68371 221.42175 0.770 0.442188

migrationLong-distance -0.65123 0.58698 185.64539 -1.109 0.268666

IUCNThreatened -0.54908 0.74015 258.26528 -0.742 0.458851

body_mass -0.05555 0.21301 363.22079 -0.261 0.794419

clutch_size 0.43548 0.25498 206.19099 1.708 0.089162 .

lifespan 0.06051 0.30873 222.83989 0.196 0.844789

natal_dispersal 0.67360 0.28431 212.46859 2.369 0.018720 *

thermal_max -0.03246 0.29612 323.97455 -0.110 0.912783

thermal_range 0.15235 0.30701 295.67209 0.496 0.620100

hist_range -0.02550 0.35244 266.25618 -0.072 0.942382

regionFennoscandia 5.86549 0.42822 272.30940 13.697 < 2e-16 ***

regionNorthwestern Europe -3.52858 0.47991 263.16963 -7.353 2.46e-12 ***

---

R2m R2c

[1,] 0.53964 0.5783645

##### Change-type (shift): both study (authors) and species (eng_name) included as random factors

Generalized linear mixed model fit by maximum likelihood (Laplace Approximation) ['glmerMod']

Family: binomial ( logit )

Formula: shift_type ~ H_type + H_breadth_sum + D_breadth_sum + D_type +

migration + IUCN + body_mass + clutch_size + lifespan + natal_dispersal +

thermal_max + thermal_range + hist_range + region + (1 | eng_name) + (1 | authors)

Data: EC_glmm

Control: glmerControl(optimizer = "bobyqa", optCtrl = list(maxfun = 1e+06))

AIC BIC logLik deviance df.resid

649.6 787.7 -293.8 587.6 605

Scaled residuals:

Min 1Q Median 3Q Max

-5.4171 -0.4907 0.2429 0.5374 3.9153

Random effects:

Groups Name Variance Std.Dev.

eng_name (Intercept) 0.2251 0.4745

authors (Intercept) 0.4970 0.7050

Number of obs: 636, groups: eng_name, 244; authors, 33

Fixed effects:

Estimate Std. Error z value Pr(>|z|)

(Intercept) 1.875028 0.864326 2.169 0.030056 *

H_typeAgriculture_Grasslands -2.756427 0.892365 -3.089 0.002009 **

H_typeForest -1.178894 0.757875 -1.556 0.119821

H_typeInland wetlands -1.877053 0.752730 -2.494 0.012643 *

H_typeTundraMireMoor -3.394515 0.995339 -3.410 0.000649 ***

H_typeWood_Shrubland -1.677300 0.704018 -2.382 0.017197 *

H_typeOther -1.234079 1.039107 -1.188 0.234978

H_breadth_sum -0.220775 0.159468 -1.384 0.166222

D_breadth_sum -0.511744 0.224515 -2.279 0.022647 *

D_typeHerbi-invertivore -0.263563 0.740272 -0.356 0.721814

D_typeHerbivore 0.809563 0.564116 1.435 0.151259

D_typeInvertivore -0.003148 0.435669 -0.007 0.994235

D_typeOmnicarnivore 2.081062 0.901737 2.308 0.021008 *

D_typeOmnivore 1.345269 0.906664 1.484 0.137873

D_typePiscivore 1.002257 0.881472 1.137 0.255528

migrationMixed 0.608003 0.362220 1.679 0.093240 .

migrationShort-distance 0.563335 0.424801 1.326 0.184802

migrationLong-distance 0.000653 0.368431 0.002 0.998586

IUCNThreatened -1.507915 0.462095 -3.263 0.001102 **

body_mass -0.301326 0.141363 -2.132 0.033042 *

clutch_size 0.096796 0.167976 0.576 0.564447

lifespan 0.367262 0.205180 1.790 0.073461 .

natal_dispersal 0.026124 0.192411 0.136 0.892003

thermal_max -0.139338 0.195051 -0.714 0.475001

thermal_range 0.062076 0.185572 0.335 0.737994

hist_range -0.167686 0.217092 -0.772 0.439867

regionFennoscandia 1.557543 0.752819 2.069 0.038551 *

regionIberian peninsula 1.711833 0.790288 2.166 0.030304 *

regionNorthwestern Europe -2.245478 0.628117 -3.575 0.000350 ***

---

R2m R2c

theoretical 0.4141208 0.5195757

delta 0.3729298 0.4678955

##### Change-type (shift): only species (eng_name) included as random factor

Generalized linear mixed model fit by maximum likelihood (Laplace Approximation) ['glmerMod']

Family: binomial ( logit )

Formula: shift_type ~ H_type + H_breadth_sum + D_breadth_sum + D_type +

migration + IUCN + body_mass + clutch_size + lifespan + natal_dispersal +

thermal_max + thermal_range + hist_range + region + (1 | eng_name)

Data: EC_glmm

Control: glmerControl(optimizer = "bobyqa", optCtrl = list(maxfun = 1e+06))

AIC BIC logLik deviance df.resid

656.1 789.7 -298.0 596.1 606

Scaled residuals:

Min 1Q Median 3Q Max

-5.6707 -0.5377 0.2689 0.5733 4.5861

Random effects:

Groups Name Variance Std.Dev.

eng_name (Intercept) 0.1314 0.3625

Number of obs: 636, groups: eng_name, 244

Fixed effects:

Estimate Std. Error z value Pr(>|z|)

(Intercept) 1.680870 0.737244 2.280 0.022611 *

H_typeAgriculture_Grasslands -2.731837 0.845277 -3.232 0.001230 **

H_typeForest -1.145187 0.719628 -1.591 0.111529

H_typeInland wetlands -1.720086 0.711670 -2.417 0.015650 *

H_typeTundraMireMoor -3.299590 0.935374 -3.528 0.000419 ***

H_typeWood_Shrubland -1.637706 0.671014 -2.441 0.014661 *

H_typeOther -1.226080 0.977590 -1.254 0.209774

H_breadth_sum -0.227785 0.150183 -1.517 0.129338

D_breadth_sum -0.463666 0.213839 -2.168 0.030136 *

D_typeHerbi-invertivore -0.257502 0.703737 -0.366 0.714435

D_typeHerbivore 0.785699 0.535408 1.467 0.142246

D_typeInvertivore -0.006326 0.412462 -0.015 0.987763

D_typeOmnicarnivore 1.996339 0.866696 2.303 0.021257 *

D_typeOmnivore 1.171778 0.860274 1.362 0.173167

D_typePiscivore 0.894573 0.827888 1.081 0.279898

migrationMixed 0.570345 0.344187 1.657 0.097503 .

migrationShort-distance 0.518994 0.396627 1.309 0.190697

migrationLong-distance 0.002402 0.348764 0.007 0.994504

IUCNThreatened -1.446766 0.434488 -3.330 0.000869 ***

body_mass -0.286621 0.132430 -2.164 0.030440 *

clutch_size 0.104433 0.160033 0.653 0.514030

lifespan 0.393889 0.194217 2.028 0.042551 *

natal_dispersal 0.038626 0.179064 0.216 0.829215

thermal_max -0.169681 0.177078 -0.958 0.337949

thermal_range 0.038791 0.172832 0.224 0.822412

hist_range -0.128601 0.196677 -0.654 0.513199

regionFennoscandia 1.874480 0.344835 5.436 5.45e-08 ***

regionIberian peninsula 1.665640 0.479430 3.474 0.000512 ***

regionNorthwestern Europe -1.835201 0.315385 -5.819 5.92e-09 ***

---

R2m R2c

theoretical 0.4399665 0.4614761

delta 0.3701616 0.3882585

**Appendix 1 – Methods, additional information**

**Search queries:**

Web of Science

1. ALL=(distribut* OR occupancy OR biogeography OR “range shift” OR "range change" OR expansion OR contraction) AND ALL=(bird* OR avifauna) AND ALL=(europ* OR “western palearctic” OR palearctic OR Scandinavia OR Mediterranean OR British OR Baltic OR Balkan) NOT ALL=(genet*) NOT ALL=(infection OR immunity OR disease) NOT ALL=(sexual OR selection) AND pubyear > 1969

AND WC=(Ecology or Ornithology or Biodiversity Conservation or Environmental Sciences or Zoology or Multidisciplinary Sciences or Evolutionary Biology or Geography Physical or Biology or Marine Freshwater Biology or Behavioral Sciences or Forestry or Environmental Studies or Agriculture Multidisciplinary or Geography or Remote Sensing or Computer Science Theory Methods ) AND SU=(Environmental Sciences Ecology OR Zoology OR Biodiversity Conservation OR Science Technology Other Topics OR Physical Geography OR Forestry OR Agriculture)

1. (distribution OR range OR occupancy OR biogeography) AND (shift OR change OR size OR expansion OR contraction OR reduction OR northward OR latitudinal) AND (bird* OR avifauna)) NOT (mammal* OR carnivore OR fish OR insect) AND (europ* OR palearctic OR scandinavia* OR british OR baltic OR balkan OR mediterranean) NOT (asia OR china OR america OR canada) AND Ecology or Ornithology or Biodiversity Conservation or Environmental Sciences or Zoology or Multidisciplinary Sciences or Evolutionary Biology or Geography Physical or Biology or Behavioral Sciences (Web of Science Categories)

Scopus

1. ( TITLE-ABS-KEY ( distribut* OR occupancy OR biogeography OR "range shift" OR "range change" OR expansion OR contraction OR reduction ) AND TITLE-ABS-KEY ( bird* OR avifauna ) AND TITLE-ABS-KEY ( europ* OR "western palearctic" OR palearctic OR scandinavia OR mediterranean OR british OR baltic OR balkan ) AND NOT TITLE-ABS-KEY ( carnivore* OR mammal* OR insect ) AND NOT TITLE-ABS-KEY ( asia OR america ) AND NOT TITLE-ABS-KEY ( infection OR immunity OR disease ) AND NOT TITLE-ABS-KEY ( sexual OR selection ) AND NOT TITLE-ABS-KEY ( foraging ) ) AND PUBYEAR > 1969 AND ( LIMIT-TO ( SUBJAREA , "AGRI" ) OR LIMIT-TO ( SUBJAREA , "ENVI" ) OR LIMIT-TO ( SUBJAREA , "BIOC" ) OR LIMIT-TO ( SUBJAREA , "EART" ) OR LIMIT-TO ( SUBJAREA , "MULT" ) )

**
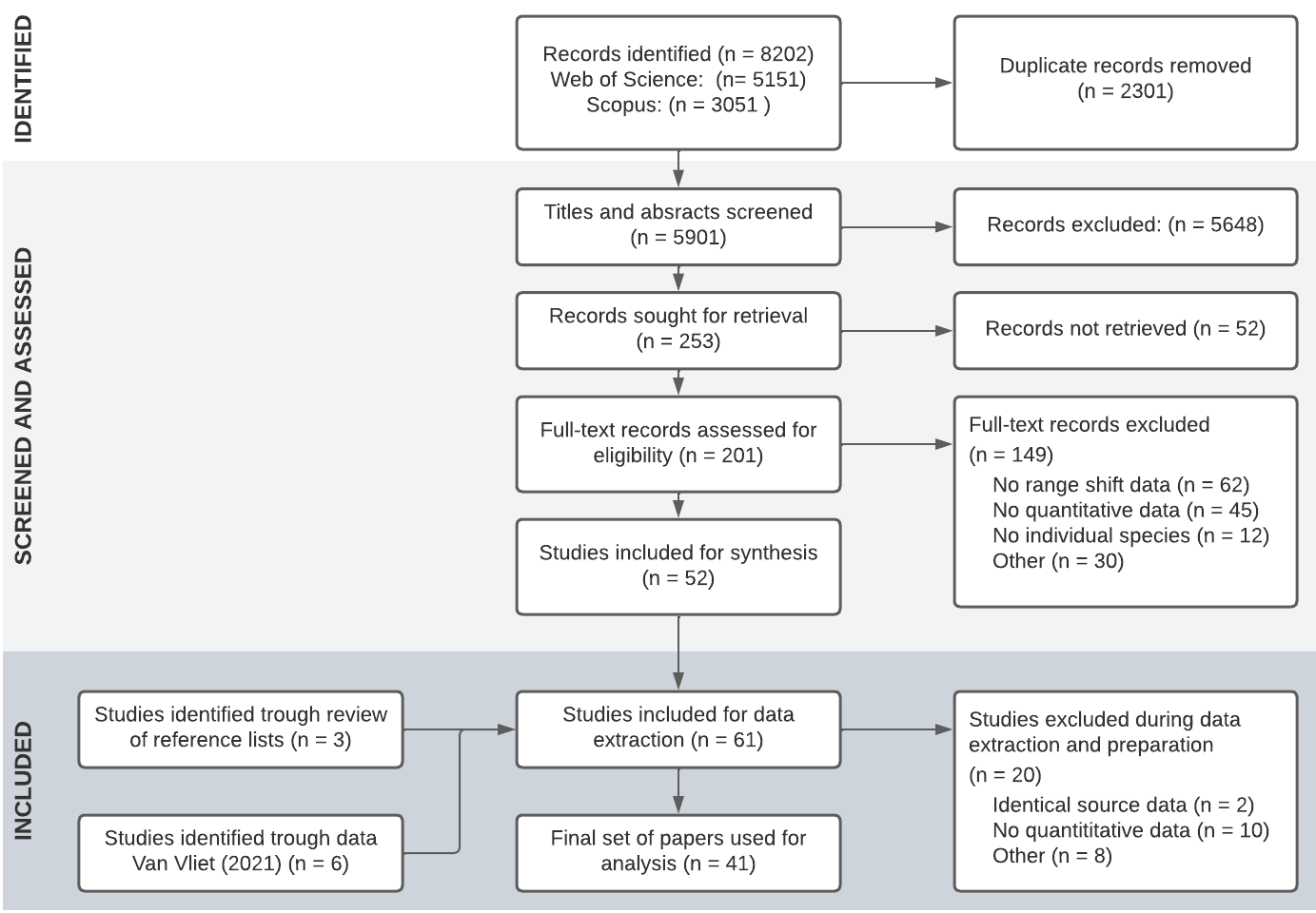
Structured literature review flow diagram, adapted from Page et al. (2021):**

**Appendix 2 – Source papers**

**Reference list of the papers used for range shift data collection.**

Arroyo, B., Lafitte, J., Sourp, E., Rousseau, D., Albert, L., Heuacker, V., Terrasse, J. F., & Razin, M. (2021). Population expansion and breeding success of Bearded Vultures Gypaetus barbatus in the French Pyrenees: results from long-term population monitoring. Ibis, 163(1), 213-230. <https://doi.org/10.1111/ibi.12852>

Assandri, G., & Morganti, M. (2015). Is the Spectacled Warbler Sylvia conspicillata expanding northward because of climate warming? Bird Study, 62(1), 126-131. <https://doi.org/10.1080/00063657.2014.988600>

Banks, A. N., Crick, H. Q. P., Coombes, R., Benn, S., Ratcliffe, D. A., & Humphreys, E. M. (2010). The breeding status of Peregrine Falcons Falco peregrinus in the UK and Isle of Man in 2002. Bird Study, 57(4), 421-436. <https://doi.org/10.1080/00063657.2010.511148>

Böhning-Gaese, K., & Bauer, H. G. (1996). Changes in species abundance, distribution, and diversity in a central European bird community. Conservation Biology, 10(1), 175-187. <https://doi.org/10.1046/j.1523-1739.1996.10010175.x>

Brommer, J. E. (2004). The range margins of northern birds shift polewards. Annales Zoologici Fennici, 41(2), 391-397.

Brommer, J. E., Lehikoinen, A., & Valkama, J. (2012). The Breeding Ranges of Central European and Arctic Bird Species Move Poleward. PloS one, 7(9), 7, Article e43648. <https://doi.org/10.1371/journal.pone.0043648>

Brotons, L., Herrando, S., & Pons, P. (2008). Wildfires and the expansion of threatened farmland birds: the ortolan bunting Emberiza hortulana in Mediterranean landscapes. Journal of Applied Ecology, 45(4), 1059-1066. <https://doi.org/10.1111/j.1365-2664.2008.01467.x>

Carrillo, C., Barbosa, A., Valera, F., Barrientos, R., & Moreno, E. (2007). Northward expansion of a desert bird: effects of climate change? Ibis, 149(1), 166-169.

Conway, G., Wotton, S., Henderson, I., Eaton, M., Drewitt, A., & Spencer, J. (2009). The status of breeding Woodlarks Lullula arborea in Britain in 2006. Bird Study, 56, 310-325, Article Pii 913194031. <https://doi.org/10.1080/00063650902792163>

Conway, G., Wotton, S., Henderson, I., Langston, R., Drewitt, A., & Currie, F. (2007). Status and distribution of European Nightiars Caprimuigus europaeus in the UK in 2004. Bird Study, 54, 98-111. <https://doi.org/10.1080/00063650709461461>

Donald, P. F., & Greenwood, J. J. D. (2001). Spatial patterns of range contraction in British breeding birds. Ibis, 143(4), 593-601. <https://doi.org/10.1111/j.1474-919X.2001.tb04887.x>

Dougall, T. W., Holland, R. K., & Yalden, D. W. (2010). The population biology of Common Sandpipers in Britain. British Birds, 103(2), 100-114.

Engström, H. (2001). The occurrence of the Great Cormorant Phalacrocorax carbo in Sweden, with special emphasis on the recent population growth. Ornis Svecica, 11(3), 155-170.

Ferrer, X., Motis, A., & Peris, S. J. (1991). Changes in the breeding range of starlings in the Iberian Peninsula during the last 30 years: competition as a limiting factor. Journal of Biogeography, 18(6), 631-636. <https://doi.org/10.2307/2845544>

Fuller, R. J., Gregory, R. D., Gibbons, D. W., Marchant, J. H., Wilson, J. D., Baillie, S. R., & Carter, N. (1995). Population declines and range contractions among lowland farmland birds in Britain. Conservation Biology, 9(6), 1425-1441. <https://doi.org/10.1046/j.1523-1739.1995.09061425.x>

Fuller, R. J., Noble, D. G., Smith, K. W., & Vanhinsbergh, D. (2005). Recent declines in populations of woodland birds in Britain: A review of possible causes. British Birds, 98(3), 116-143.

Gil-Tena, A., Brotons, L., & Saura, S. (2009). Mediterranean forest dynamics and forest bird distribution changes in the late 20th century. Global change biology, 15(2), 474-485. <https://doi.org/10.1111/j.1365-2486.2008.01730.x>

Gregory, R. D., Wilkinson, N. I., Noble, D. G., Robinson, J. A., Brown, A. F., Hughes, J., Procter, D., Gibbons, D. W., & Galbraith, C. A. (2002). The population status of birds in the United Kingdom, Channel Islands and Isle of Man: An analysis of conservation concern 2002-2007. British Birds, 95(9), 410-448.

Henderson, I., Calladine, J., Massimino, D., Taylor, J. A., & Gillings, S. (2014). Evidence for contrasting causes of population change in two closely related, sympatric breeding species the Whinchat Saxicola rubetra and Stonechat Saxicola torquata in Britain. Bird Study, 61(4), 553-565. <https://doi.org/10.1080/00063657.2014.962482>

Henderson, I., Wilson, A., Steele, D., & Vickery, J. (2002). Population estimates, trends and habitat associations of breeding Lapwing Vanellus vanellus, Curlew Numenius arquata and Snipe Gallinago gallinago in Northern Ireland in 1999. Bird Study, 49(1), 17-25.

Heward, C. J., Hoodless, A. N., Conway, G. J., Aebischer, N. J., Gillings, S., & Fuller, R. J. (2015). Current status and recent trend of the Eurasian Woodcock Scolopax rusticola as a breeding bird in Britain. Bird Study, 62(4), 535-551. <https://doi.org/10.1080/00063657.2015.1092497>

Kolecek, J., & Reif, J. (2011). Differences between the predictors of abundance, trend and distribution as three measures of avian population change. Acta Ornithologica, 46(2), 143-153. <https://doi.org/10.3161/000164511x625919>

Lehikoinen, A., & Virkkala, R. (2016). North by north-west: climate change and directions of density shifts in birds. Global change biology, 22(3), 1121-1129. https://doi.org/10.1111/gcb.13150

Maclean, I. M. D., Austin, G. E., Rehfisch, M. M., Blew, J., Crowe, O., Delany, S., Devos, K., Deceuninck, B., Gunther, K., Laursen, K., Van Roomen, M., & Wahl, J. (2008). Climate change causes rapid changes in the distribution and site abundance of birds in winter. Global change biology, 14(11), 2489-2500. <https://doi.org/10.1111/j.1365-2486.2008.01666.x>

Marion, L., & Bergerot, B. (2018). Northern range shift may be due to increased competition induced by protection of species rather than to climate change alone [Article]. Ecology and Evolution, 8(16), 8364-8379. <https://doi.org/10.1002/ece3.4348>

Mason, S. C., Palmer, G., Fox, R., Gillings, S., Hill, J. K., Thomas, C. D., & Oliver, T. H. (2015). Geographical range margins of many taxonomic groups continue to shift polewards. Biological Journal of the Linnean Society, 115(3), 586-597. <https://doi.org/10.1111/bij.12574>

Pagel, J., Martinez-Abrain, A., Gomez, J. A., Jimenez, J., & Oro, D. (2014). A Long-Term Macroecological Analysis of the Recovery of a Waterbird Metacommunity after Site Protection. PloS one, 9(8), 13, Article e105202. <https://doi.org/10.1371/journal.pone.0105202>

Potvin, D. A., Välimäki, K., & Lehikoinen, A. (2016). Differences in shifts of wintering and breeding ranges lead to changing migration distances in European birds. Journal of Avian Biology, 47(5), 619-628. <https://doi.org/10.1111/jav.00941>

Purger, J. J. (2008). Numbers and distribution of red-footed falcons (Falco vespertinus) breeding in Voivodina (northern Serbia): A comparison between 1990-1991 and 2000-2001. Belgian Journal of Zoology, 138(1), 3-7.

Raine, A. F., Brown, A. F., Amano, T., & Sutherland, W. J. (2009). Assessing population changes from disparate data sources: the decline of the Twite Carduelis flavirostris in England. Bird Conservation International, 19(4), 401-416. <https://doi.org/10.1017/s0959270909990086>

Reif, J., St'astny, K., & Bejcek, V. (2010). Contrasting effects of climatic and habitat changes on birds with northern range limits in central Europe as revealed by an analysis of breeding bird distribution in the Czech Republic. Acta Ornithologica, 45(1), 83-90. <https://doi.org/10.3161/000164510x516128>

Stanbury, A., Davies, M., Grice, P., Gregory, R., & Wotton, S. (2010). The status of the Cirl Bunting in the UK in 2009. British Birds, 103(12), 702-711.

Tayleur, C., Caplat, P., Massimino, D., Johnston, A., Jonzen, N., Smith, H. G., & Lindstrom, A. (2015). Swedish birds are tracking temperature but not rainfall: evidence from a decade of abundance changes. Global Ecology and Biogeography, 24(7), 859-872. https://doi.org/10.1111/geb.12308

Taylor, K., Hudson, R., & Horne, G. (1988). Buzzard breeding distribution and abundance in britain and northern ireland in 1983. Bird Study, 35(2), 109-118. <https://doi.org/10.1080/00063658809480387>

Thorup, K., Sunde, P., Jacobsen, L. B., & Rahbek, C. (2010). Breeding season food limitation drives population decline of the Little Owl Athene noctua in Denmark. Ibis, 152(4), 803-814. <https://doi.org/10.1111/j.1474-919X.2010.01046.x>

Treinys, R., Dementavičius, D., Rumbutis, S., Švažas, S., Butkauskas, D., Sruoga, A., & Dagys, M. (2016). Settlement, habitat preference, reproduction, and genetic diversity in recovering the white-tailed eagle haliaeetus albicilla population. Journal of Ornithology, 157(1), 311-323. <https://doi.org/10.1007/s10336-015-1280-8>

Valera, F., Rey, P., Sanchez-Lafuente, A. M., & Muñoz-Cobo, J. (1993). Expansion of Penduline Tit (Remiz pendulinus) through migration and wintering. Journal Fur Ornithologie, 134(3), 273-282.

Välimäki, K., Linden, A., & Lehikoinen, A. (2016). Velocity of density shifts in Finnish landbird species depends on their migration ecology and body mass. Oecologia, 181(1), 313-321. <https://doi.org/10.1007/s00442-015-3525-x>

Van Rijn, S. (2018). Broedende Rode Wouwen in Nederland in 1976-2017. Limosa, 91(1), 3-15.

Velevski, M., Nikolov, S. C., Ben, H., Dobrev, V., Sidiropoulos, L., Saravia, V., Tsiakiris, R., Arkumarev, V., Galanaki, A., Kominos, T., Stara, K., Kret, E., Grubac, B., Lisicanec, E., Kastritis, T., Vavylis, D., Topi, M., Hoxha, B., & Oppel, S. (2015). Population decline and range contraction of the Egyptian Vulture Neophron percnopterus in the Balkan Peninsula. Bird Conservation International, 25(4), 440-450. <https://doi.org/10.1017/s0959270914000343>

Virkkala, R., Heikkinen, R. K., Lehikoinen, A., & Valkama, J. (2014). Matching trends between recent distributional changes of northern-boreal birds and species-climate model predictions. Biological Conservation, 172, 124-127. <https://doi.org/10.1016/j.biocon.2014.01.041>

Virkkala, R., & Lehikoinen, A. (2017). Birds on the move in the face of climate change: High species turnover in northern Europe. Ecology and Evolution, 7(20), 8201-8209. <https://doi.org/10.1002/ece3.3328>

Zielińska, M., Zieliński, P., Kołodziejczyk, P., Szewczyk, P., & Betleja, J. (2007). Expansion of the Mediterranean gull Larus melanocephalus in Poland. Journal of Ornithology, 148(4), 543-548.

**Appendix 3 – Supplementary tables**

Supplementary file with all supplementary tables (S1-S7).

Filename: Warmer et al._Appx3.docx

**Appendix 4 – Model comparisons full models**

Supplementary file with all full model comparisons.

Filename: Warmer et al._Appx4.docx

**Appendix 5 – Residual diagnostics**

DHARMa residual diagnostics output for Change-type:


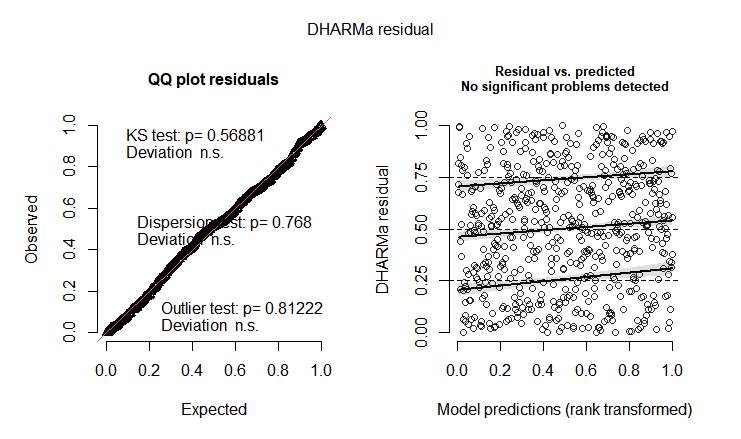


DHARMa residual diagnostics output for Relative-change before transformation:


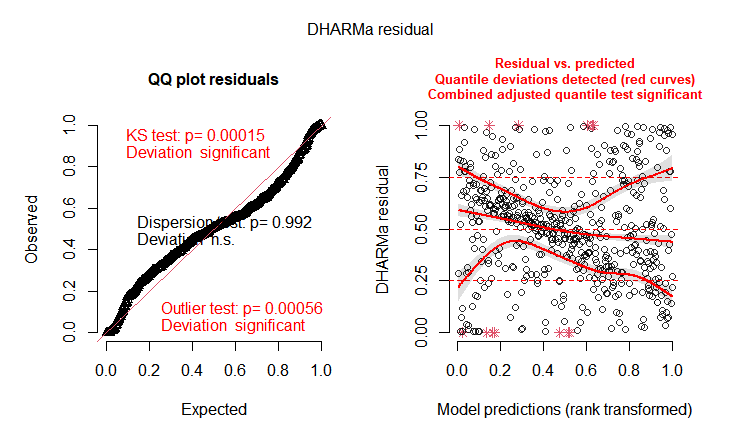
DHARMa residual diagnostics output for Relative-change

after log-modulus transformation:


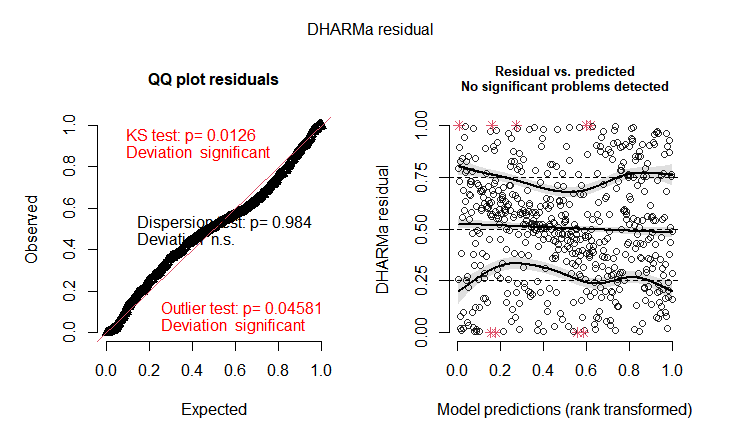


DHARMa residual diagnostics output for Rate-of-change before transformation:


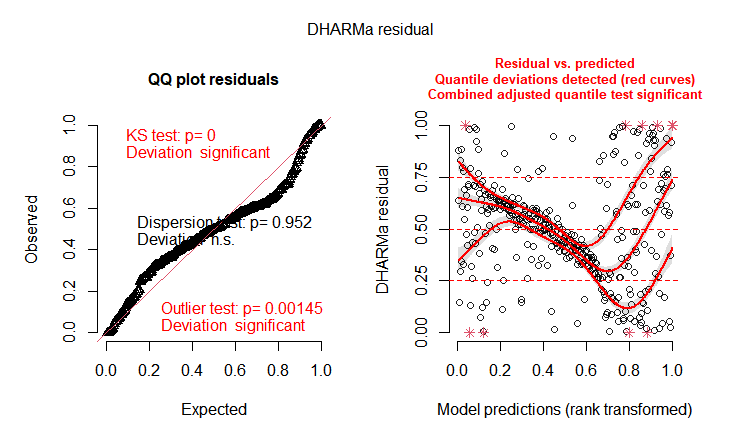


DHARMa residual diagnostics output for Rate-of-change after log-modulus transformation:


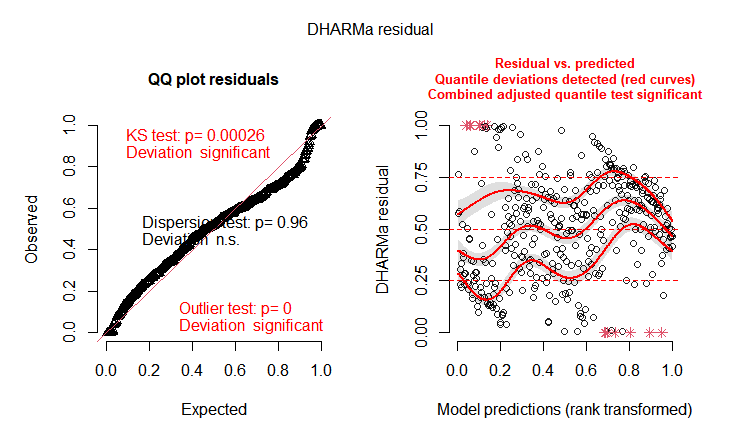


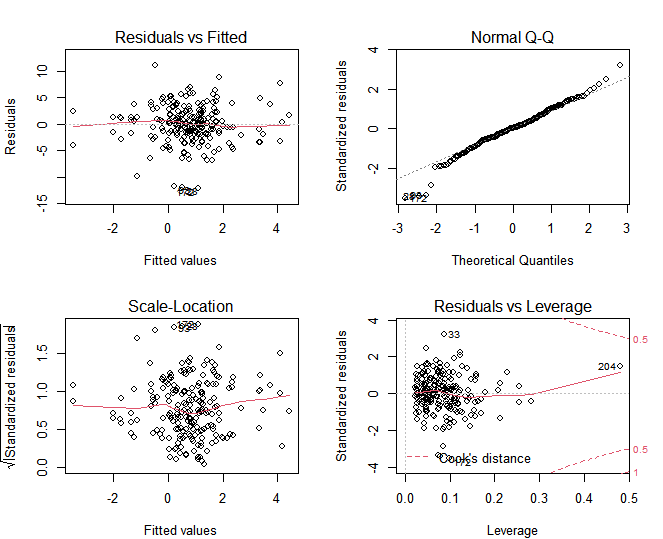
Residual diagnostics plots for Abundance shift:

Residual diagnostics plots for Centroid shift before transformation:


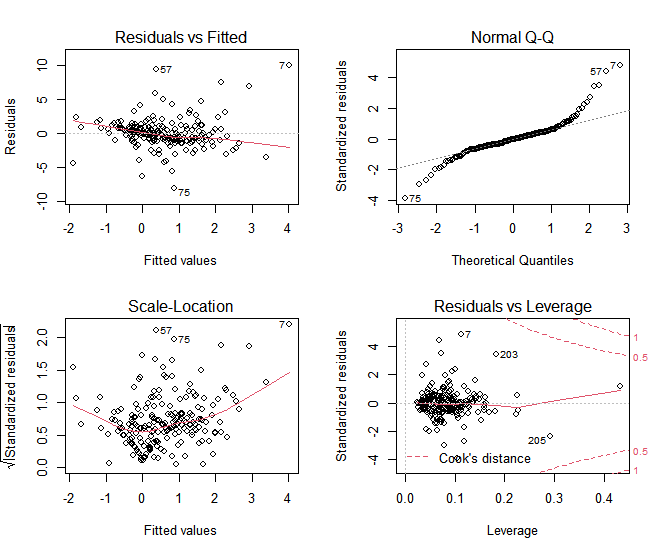

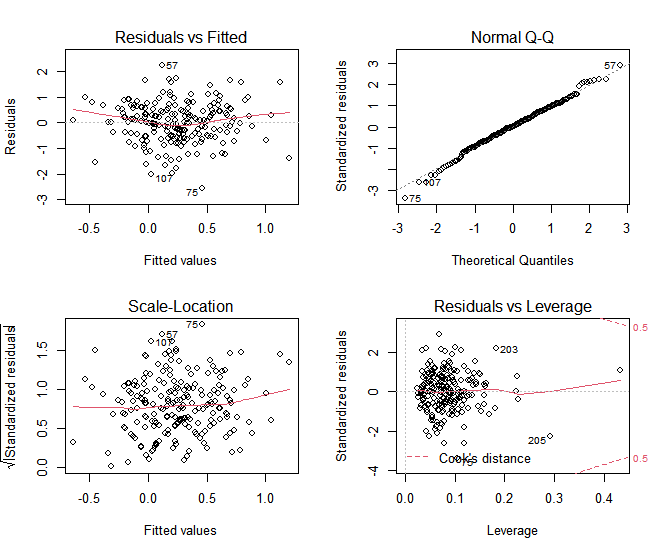


Residual diagnostics plots for Centroid shift after log-modulus transformation:


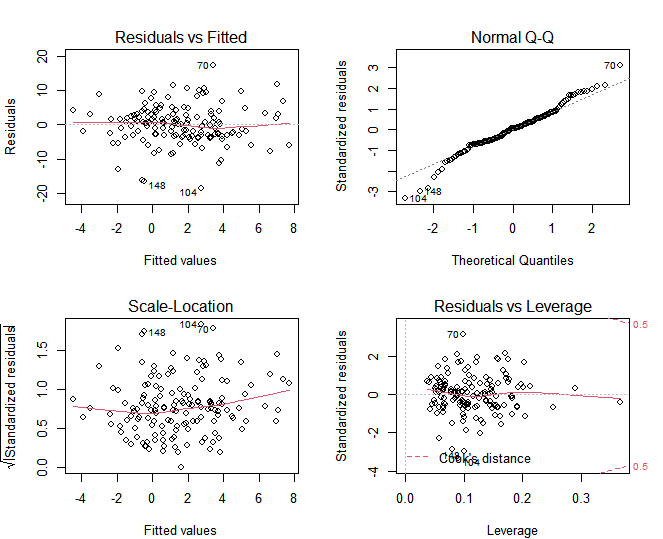


Residual diagnostics plots for

N-margin shift:

**Appendix 6 - Supplementary Data File**

The accompanying Excel spreadsheet contains the raw data used in this study, including a sheet with the raw range shift datapoints for each range shift category including species traits, a sheet with a species list, and a sheet with the species trait data.

Filename: Warmer et al._Appx6.xlsx

**Appendix 7 - Supplementary Data File**

The accompanying text file shows the R script for the calculation of the species thermal maximum, minimum and range, as well as the calculation of the historical range variables.

Filename: Appendix 7 in Dryad: Dataset DOI: [10.5061/dryad.wstqjq2z5](https://datadryad.org/submission/10.5061/dryad.wstqjq2z5)

**Appendix 8 - Supplementary Data File**

The accompanying text file shows the R script for model selection and multi-model inference of the Range size change dataset, including the three range shift types Change-type, Relative-change, and Rate-of-change.

Filename: Appendix 8 in Dryad: Dataset DOI: [10.5061/dryad.wstqjq2z5](https://datadryad.org/submission/10.5061/dryad.wstqjq2z5)

**Appendix 9 - Supplementary Data File**

The accompanying text file shows the R script for model selection and multi-model inference of the Abundance shift dataset.

Filename: Appendix 9 in Dryad: Dataset DOI: [10.5061/dryad.wstqjq2z5](https://datadryad.org/submission/10.5061/dryad.wstqjq2z5)

**Appendix 10 - Supplementary Data File**

The accompanying text file shows the R script for model selection and multi-model inference of the Centroid shift dataset.

Filename: Appendix 6 in Dryad: Dataset DOI: [10.5061/dryad.wstqjq2z5](https://datadryad.org/submission/10.5061/dryad.wstqjq2z5)

**Appendix 11 - Supplementary Data File**

The accompanying text file shows the R script for model selection and multi-model inference of the Northern range margin shift dataset.

Filename: Appendix 11 in Dryad: Dataset DOI: [10.5061/dryad.wstqjq2z5](https://datadryad.org/submission/10.5061/dryad.wstqjq2z5)

**Appendix 12 – Histogram plots**


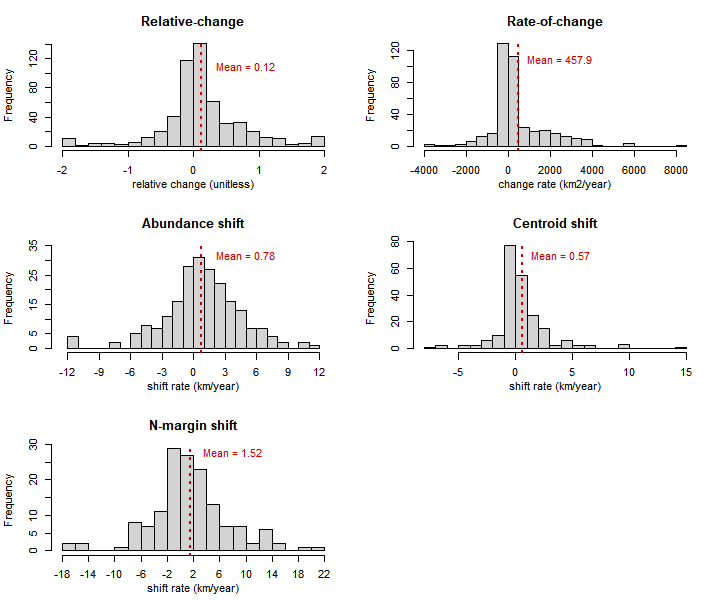
The figure below shows the histogram plots with indicated mean value of the range shift data for all range shift types except Change-type.
