# Supplementary material for: The Role of Intrinsic Factors in Explaining Range Shifts of European Breeding Birds: A Meta‐Analysis
Source: Ecol Evol. 2025 Apr 21;15(4):e71308. doi: 10.1002/ece3.71308 (PMC12012262; doi:10.1002/ece3.71308)
Supplement: Supplementary file 1 — Data S1. [file ECE3-15-e71308-s001.zip › Warmer et al._Appx3.docx]

**Appendix 3 – Supplementary tables**

**Table S1** Model calls used in the model selection and multi-model inference process.

**Table S2** Output of the model selection for Change-type using generalized linear mixed models. All models with Δi≤2 are reported with **x** indicating when a predictor (with **x** for categories of that predictor) was included in a model. Per model, positive coefficients are shaded green and negative coefficients are shaded red. Akaike weights (wi, indicating the relative likelihood of a model (Burnham & Anderson, 2004)) and marginal R2 values are reported for each of the top-ranked models. For each predictor variable, the model averaged regression coefficient β and its confidence interval (CI) are reported, plus the exponent of β for odds ratios. The predictor specific w_sum_ is the sum of w_i_ for the models a predictor is included in and indicates the relative importance of a predictor variable. w_sum_ is 1 when a predictor is included in all top-ranked models. p-values are reported, and bold values mark significant β values with CI not including zero. Per model significances are illustrated with asterisks.

**Table S3** Output of the model selection for Relative-change using linear mixed models. Table components and variables are as explained for Table S2.

**Table S4** Output of the model selection for Rate-of-change using linear mixed models. Table components and variables are as explained for Table S2.

**Table S5** Output of the model selection for Abundance shift using linear models. Table components and variables are as explained for Table S2.

**Table S6** Output of the model selection for Centroid shift using linear models. Table components and variables are as explained for Table S2.

**Table S7** Output of the model selection for N-margin shift using linear models. Table components and variables are as explained for Table S2.

**Table S1**

|  |  | | |  |  |  |
| --- | --- | --- | --- | --- | --- | --- |
| **Response variables** | **Range size change** | | | **Abundance shift** | **Centroid shift** | **N-margin shift** |
|  | **Change-type^a^** | **Relative-change^b^** | **Rate-of-change^c^** |  |  |  |
| **Type of test** | glmm | lmm | lmm | lm | lm | lm |
| **n** | 211/368 | 546 | 394 | 213 | 211 | 149 |
|  |  |  |  |  |  |  |
| **Predictor variables** |  |  |  |  |  |  |
| Habitat type | **✓** | **✓** | **✓** | **x** | **x** | **x** |
| Habitat breadth | **✓** | **✓** | **✓** | **✓** | **✓** | **✓** |
| Diet type | **✓** | **✓** | **✓** | **✓** | **✓** | **✓** |
| Diet breadth | **✓** | **✓** | **✓** | **✓** | **✓** | **✓** |
| Body mass | **✓** | **✓** | **✓** | **✓** | **✓** | **✓** |
| Clutch size | **✓** | **✓** | **✓** | **✓** | **✓** | **✓** |
| Lifespan | **✓** | **✓** | **✓** | **✓** | **✓** | **✓** |
| Migration strategy | **✓** | **✓** | **✓** | **✓** | **✓** | **✓** |
| Natal dispersal | **✓** | **✓** | **✓** | **✓** | **✓** | **✓** |
| IUCN status | **✓** | **✓** | **✓** | **✓** | **✓** | **✓** |
| Thermal max | **✓** | **✓** | **✓** | **✓** | **✓** | **✓** |
| Thermal range | **✓** | **✓** | **✓** | **x** | **x** | **x** |
| Historical range size | **✓** | **✓** | **✓** | **✓** | **✓** | **✓** |
| Historical northern limit | **x** | **x** | **x** | **✓** | **✓** | **✓** |
| Region | **✓** | **✓** | **✓** | **x** | **x** | **✓** |
|  |  |  |  |  |  |  |
| **Random factor** | Species | Species | Species | x | x | x |
| **Notes** | Binomial distribution | Log-modulus transformed | Log-modulus transformed |  | Log modulus transformed |  |

a Range contraction or range expansion

b Relative-change = (range size at time 2 – range size at time 1)/((range size at time 2 + range size at time 1)/2)

c Rate of the change in range size (km2/year)

**Table S2**

|  |  |  |  |  |  |  |  |  |  |  |  |  |  |  |  |  |  |  |  |  |  |  |  |  |  |  | |  | |  | |  | |  | |  | |  |  |  |  |
| --- | --- | --- | --- | --- | --- | --- | --- | --- | --- | --- | --- | --- | --- | --- | --- | --- | --- | --- | --- | --- | --- | --- | --- | --- | --- | --- | --- | --- | --- | --- | --- | --- | --- | --- | --- | --- | --- | --- | --- | --- | --- |
|  |  | glmm - Model rank | | | | | | | | | | | | | | | | | | | | | | | | |  | |  | |  | |  | |  | | Model average | | | |  |
|  |  | 1 | 2 | 3 | 4 | 5 | 6 | 7 | 8 | 9 | 10 | 11 | 12 | 13 | 14 | 15 | 16 | 17 | 18 | 19 | 20 | 21 | 22 | 23 | 24 | 25 | | 26 | | 27 | | 28 | |  | | β | | 95% CI | w_sum_ | exp β | P |
| **Intercept** | | **x**** | **x**** | **x**** | **x**** | **x**** | **x**** | **x**** | **x**** | **x**** | **x**** | **x**** | **x**** | **x**** | **x**** | **x**** | **x**** | **x**** | **x**** | **x**** | **x*** | **x**** | **x**** | **x*** | **x***** | **x**** | | **x**** | | **x**** | | **x**** | |  | | **1.75** | | **0.46 to 3.03** | **-** | **-** | **0.008** |
| **Habitat type (ref: habitat generalist)** | | **x**** | **x**** | **x**** | **x**** | **x**** | **x**** | **x**** | **x**** | **x**** | **x**** | **x**** | **x**** | **x**** | **x**** | **x**** | **x**** | **x**** | **x**** | **x**** | **x**** | **x**** | **x**** | **x**** | **x**** | **x**** | | **x**** | | **x**** | | **x**** | |  | |  | |  | **1.00** |  |  |
| **Agriculture & Grasslands** |  | **x**** | **x**** | **x**** | **x***** | **x***** | **x**** | **x***** | **x**** | **x***** | **x**** | **x**** | **x**** | **x***** | **x***** | **x**** | **x***** | **x**** | **x***** | **x**** | **x**** | **x**** | **x***** | **x**** | **x***** | **x**** | | **x**** | | **x***** | | **x***** | |  | | **-2.57** | | **-4.28 to -0.87** |  | **0.08** | **0.003** |
| Forest |  | x | x | x | x | x | x | x | x | x | x | x | x | x* | x | x | x | x | x | x | x | x | x | x | x | x | | x | | x | | x | |  | | -0.83 | | -2.29 to 0.62 |  | 0.46 | 0.260 |
| Inland wetlands |  | x* | x | x | x* | x* | x* | x* | x | x** | x | x* | x* | x** | x* | x | x* | x | x* | x* | x | x | x* | x | x* | x* | | x* | | x** | | x* | |  | | -1.32 | | -2.76 to 0.12 |  | 0.28 | 0.072 |
| **Tundra-Mire-Moor** |  | **x***** | **x***** | **x***** | **x***** | **x***** | **x***** | **x***** | **x***** | **x***** | **x***** | **x***** | **x***** | **x***** | **x***** | **x***** | **x***** | **x***** | **x***** | **x***** | **x***** | **x***** | **x***** | **x***** | **x***** | **x***** | | **x***** | | **x***** | | **x***** | |  | | **-3.58** | | **-5.48 to -1.69** |  | **0.03** | **<0.001** |
| **Wood & Shrubland** |  | **x*** | **x*** | **x** | **x**** | **x**** | **x*** | **x*** | **x** | **x**** | **x*** | **x*** | **x*** | **x**** | **x*** | **x** | **x**** | **x** | **x**** | **x*** | **x*** | **x** | **x*** | **x*** | **x*** | **x*** | | **x*** | | **x**** | | **x*** | |  | | **-1.41** | | **-2.73 to -0.08** |  | **0.26** | **0.038** |
| Other |  | x | x | x | x | x | x | x | x | x | x | x | x | x | x | x | x | x | x | x | x | x | x | x | x | x | | x | | x | | x | |  | | -1.01 | | -2.89 to 0.87 |  | 0.39 | 0.294 |
| **IUCN status: 'Threatened'** | | **x**** | **x**** | **x**** | **x**** | **x**** | **x**** | **x**** | **x*** | **x**** | **x*** | **x**** | **x**** | **x**** | **x**** | **x*** | **x**** | **x**** | **x**** | **x**** | **x**** | **x**** | **x**** | **x**** | **x**** | **x**** | | **x**** | | **x**** | | **x**** | |  | | **-1.26** | | **-2.15 to -0.38** | **1.00** | **0.28** | **0.005** |
| **Lifespan** | | **x**** | **x**** | **x**** | **x*** | **x*** | **x**** | **x**** | **x*** | **x*** | **x*** | **x**** | **x**** | **x*** | **x**** | **x*** | **x*** | **x**** | **x**** | **x**** | **x*** | **x**** | **x** | **x** | **x**** | **x**** | | **x**** | | **x**** | | **x**** | |  | | **0.40** | | **0.08 to 0.73** | **1.00** | **1.50** | **0.014** |
| **Region (ref: Central EU)** | | **x**** | **x**** | **x**** | **x**** | **x**** | **x**** | **x**** | **x**** | **x**** | **x**** | **x**** | **x**** | **x**** | **x**** | **x**** | **x**** | **x**** | **x**** | **x**** | **x**** | **x**** | **x**** | **x**** | **x**** | **x**** | | **x***** | | **x**** | | **x**** | |  | |  | |  | **1.00** |  |  |
| **Fennoscandia** |  | **x***** | **x***** | **x***** | **x***** | **x***** | **x***** | **x***** | **x***** | **x***** | **x***** | **x***** | **x***** | **x***** | **x***** | **x***** | **x***** | **x***** | **x***** | **x***** | **x***** | **x***** | **x***** | **x***** | **x***** | **x***** | | **x***** | | **x***** | | **x***** | |  | | **2.20** | | **1.45 to 2.94** |  | **9.00** | **<0.001** |
| **Iberian Peninsula** |  | **x*** | **x*** | **x*** | **x*** | **x*** | **x*** | **x*** | **x*** | **x*** | **x*** | **x*** | **x*** | **x*** | **x*** | **x*** | **x*** | **x*** | **x*** | **x*** | **x*** | **x*** | **x*** | **x*** | **x*** | **x*** | | **x*** | | **x*** | | **x*** | |  | | **1.10** | | **0.15 to 2.05** |  | **3.02** | **0.024** |
| **North-western EU** |  | **x***** | **x***** | **x***** | **x***** | **x***** | **x***** | **x***** | **x***** | **x***** | **x***** | **x***** | **x***** | **x***** | **x***** | **x***** | **x***** | **x***** | **x***** | **x***** | **x***** | **x***** | **x***** | **x***** | **x***** | **x***** | | **x***** | | **x***** | | **x***** | |  | | **-1.86** | | **-2.49 to -1.22** |  | **0.16** | **<0.001** |
| Body mass | | x* | x | x | x | x* | x* | x | x | x* |  | x | x* | x* | x |  | x | x | x* | x* | x | x |  | x | x | x | | x* | | x* | | x | |  | | -0.22 | | -0.50 to 0.07 | 0.90 | 0.81 | 0.133 |
| Habitat breadth | | x | x |  | x | x | x | x |  | x | x | x | x | x | x |  | x |  | x | x |  |  | x |  | x | x | | x | | x | | x | |  | | -0.20 | | -0.55 to 0.14 | 0.76 | 0.82 | 0.253 |
| Historical range size | | x |  | x |  | x | x |  |  |  |  |  | x |  |  |  |  | x | x | x | x |  |  |  |  |  | | x | | x | |  | |  | | -0.09 | | -0.35 to 0.18 | 0.41 | 0.92 | 0.528 |
| Migration strategy (ref: sedentary) | |  |  |  | x | x |  |  |  | x |  |  |  | x |  |  | x |  | x |  | x |  | x | x |  |  | |  | | x | |  | |  | |  | |  | 0.34 |  |  |
| Partial |  |  |  |  | x | x |  |  |  | x |  |  |  | x |  |  | x |  | x |  | x |  | x | x |  |  | |  | | x | |  | |  | | 0.18 | | -0.45 to 0.81 |  | 1.17 | 0.575 |
| Short-distance |  |  |  |  | x* | x |  |  |  | x* |  |  |  | x* |  |  | x* |  | x |  | x |  | x* | x |  |  | |  | | x | |  | |  | | 0.25 | | -0.57 to 1.07 |  | 1.25 | 0.551 |
| Long-distance |  |  |  |  | x | x |  |  |  | x |  |  |  | x |  |  | x |  | x |  | x |  | x | x |  |  | |  | | x | |  | |  | | 0.02 | | -0.35 to 0.39 |  | 1.02 | 0.634 |
| Diet breadth | |  |  |  |  |  | x |  |  |  |  | x |  | x |  |  | x | x | x |  |  | x |  |  | x |  | | x | |  | | x | |  | | -0.05 | | -0.24 to 0.14 | 0.31 | 0.95 | 0.634 |
| Thermal Range | |  |  |  |  |  |  |  |  | x |  |  |  | x | x |  |  |  |  |  |  |  |  |  |  |  | |  | |  | | x | |  | | -0.02 | | -0.16 to 0.12 | 0.13 | 0.98 | 0.766 |
| Clutch size | |  |  |  |  |  |  |  |  |  |  |  | x |  |  |  |  |  |  |  |  |  |  |  |  | x | | x | | x | |  | |  | | 0.01 | | -0.10 to 0.13 | 0.11 | 1.01 | 0.813 |
| Thermal max | |  |  |  |  |  |  | x |  |  |  |  |  |  |  |  |  |  |  | x |  |  |  |  | x |  | |  | |  | |  | |  | | -0.02 | | -0.16 to 0.13 | 0.10 | 0.98 | 0.819 |
| Natal dispersal | |  |  |  |  |  |  |  |  |  |  |  |  |  |  |  |  |  |  |  |  |  |  |  |  |  | |  | |  | |  | |  | | - | | - | - | - | - |
| Diet type | |  |  |  |  |  |  |  |  |  |  |  |  |  |  |  |  |  |  |  |  |  |  |  |  |  | |  | |  | |  | |  | | - | | - | - | - | - |
|  |  |  |  |  |  |  |  |  |  |  |  |  |  |  |  |  |  |  |  |  |  |  |  |  |  |  | |  | |  | |  | |  | |  | |  |  |  |  |
| Δ_i_ | | 0.00 | 0.39 | 0.63 | 0.76 | 0.83 | 0.95 | 1.01 | 1.04 | 1.08 | 1.16 | 1.22 | 1.23 | 1.32 | 1.32 | 1.39 | 1.41 | 1.44 | 1.48 | 1.58 | 1.69 | 1.71 | 1.73 | 1.76 | 1.85 | 1.88 | | 1.92 | | 1.96 | | 1.96 | |  | |  | |  |  |  |  |
| w_i_ | | 0.067 | 0.055 | 0.049 | 0.046 | 0.044 | 0.042 | 0.040 | 0.040 | 0.039 | 0.037 | 0.036 | 0.036 | 0.035 | 0.034 | 0.033 | 0.033 | 0.033 | 0.032 | 0.030 | 0.029 | 0.028 | 0.028 | 0.028 | 0.026 | 0.026 | | 0.026 | | 0.025 | | 0.025 | |  | |  | |  |  |  |  |
| marginal R^2^ | | 0.421 | 0.413 | 0.414 | 0.424 | 0.430 | 0.424 | 0.419 | 0.407 | 0.430 | 0.407 | 0.419 | 0.422 | 0.434 | 0.417 | 0.401 | 0.428 | 0.418 | 0.434 | 0.424 | 0.424 | 0.411 | 0.418 | 0.418 | 0.421 | 0.415 | | 0.426 | | 0.432 | | 0.420 | |  | |  | |  |  |  |  |
|  |  |  |  |  |  |  |  |  |  |  |  |  |  |  |  |  |  |  |  |  |  |  |  |  |  |  | |  | |  | |  | |  | |  | |  |  |  |  |

Sign. codes: * p<0.05, ** p<0.01, *** p<0.001

**Table S3**

|  |  |  |  |  |  |  |  |  |  |  |  |  |  |  |  |  |  |  |  |  | |  | |  | |  |  |  |  |
| --- | --- | --- | --- | --- | --- | --- | --- | --- | --- | --- | --- | --- | --- | --- | --- | --- | --- | --- | --- | --- | --- | --- | --- | --- | --- | --- | --- | --- | --- |
|  |  | lmm - Model rank | | | | | | | | | | | | | | | | | | |  | |  | | Model average | | |  |  |
|  |  | 1 | 2 | 3 | 4 | 5 | 6 | 7 | 8 | 9 | 10 | 11 | 12 | 13 | 14 | 15 | 16 | 17 | 18 | 19 | |  | | β | | 95% CI | w_sum_ | Exp β | p |
| **Intercept** | | **x***** | **x***** | **x***** | **x***** | **x***** | **x***** | **x***** | **x***** | **x***** | **x***** | **x***** | **x***** | **x***** | **x***** | **x***** | **x***** | **x***** | **x***** | **x***** | |  | | **0.34** | | **0.19 to 0.49** | **-** | **1.40** | **<0.001** |
| **Habitat type (ref: Habitat generalist)** | | **x***** | **x***** | **x***** | **x***** | **x***** | **x***** | **x***** | **x***** | **x***** | **x***** | **x***** | **x***** | **x***** | **x***** | **x***** | **x***** | **x***** | **x***** | **x***** | |  | |  | |  | **1.00** |  |  |
| **Agriculture & Grasslands** |  | **x***** | **x***** | **x***** | **x***** | **x***** | **x***** | **x***** | **x***** | **x***** | **x***** | **x***** | **x***** | **x***** | **x***** | **x***** | **x***** | **x***** | **x***** | **x***** | |  | | **-0.44** | | **-0.66 to -0.23** |  | **0.64** | **<0.001** |
| **Forest** |  | **x**** | **x**** | **x**** | **x***** | **x**** | **x***** | **x***** | **x**** | **x**** | **x***** | **x***** | **x**** | **x**** | **x***** | **x***** | **x**** | **x**** | **x**** | **x**** | |  | | **-0.26** | | **-0.43 to -0.10** |  | **0.77** | **0.002** |
| **Inland wetlands** |  | **x***** | **x**** | **x**** | **x***** | **x**** | **x***** | **x***** | **x**** | **x**** | **x***** | **x**** | **x**** | **x***** | **x***** | **x***** | **x**** | **x**** | **x**** | **x**** | |  | | **-0.27** | | **-0.44 to -0.11** |  | **0.76** | **0.001** |
| **Tundra-Mire-Moor** |  | **x***** | **x***** | **x***** | **x***** | **x***** | **x***** | **x***** | **x***** | **x***** | **x***** | **x***** | **x***** | **x***** | **x***** | **x***** | **x***** | **x***** | **x***** | **x***** | |  | | **-0.81** | | **-1.05 to -0.58** |  | **0.44** | **<0.001** |
| **Wood & Shrubland** |  | **x***** | **x***** | **x***** | **x***** | **x***** | **x***** | **x***** | **x***** | **x***** | **x***** | **x***** | **x***** | **x***** | **x***** | **x***** | **x***** | **x***** | **x***** | **x***** | |  | | **-0.33** | | **-0.49 to -0.17** |  | **0.72** | **<0.001** |
| **Other** |  | **x***** | **x***** | **x***** | **x***** | **x***** | **x***** | **x***** | **x***** | **x**** | **x***** | **x***** | **x***** | **x**** | **x***** | **x***** | **x***** | **x***** | **x***** | **x***** | |  | | **-0.40** | | **-0.63 to -0.18** |  | **0.67** | **0.001** |
| **IUCN status: Threatened** | | **x**** | **x**** | **x**** | **x**** | **x**** | **x**** | **x**** | **x**** | **x**** | **x**** | **x**** | **x**** | **x**** | **x**** | **x**** | **x**** | **x**** | **x**** | **x**** | |  | | **-0.19** | | **-0.32 to -0.06** | **1.00** | **0.83** | **0.004** |
| **Region (ref: Central EU)** | | **x***** | **x***** | **x***** | **x***** | **x***** | **x***** | **x***** | **x***** | **x***** | **x***** | **x***** | **x***** | **x***** | **x***** | **x***** | **x***** | **x***** | **x***** | **x***** | |  | |  | |  | **1.00** |  |  |
| **Fennoscandia** |  | **x***** | **x***** | **x***** | **x***** | **x***** | **x***** | **x***** | **x***** | **x***** | **x***** | **x***** | **x***** | **x***** | **x***** | **x***** | **x***** | **x***** | **x***** | **x***** | |  | | **0.31** | | **0.24 to 0.38** |  | **1.37** | **<0.001** |
| **North-western EU** |  | **x***** | **x***** | **x***** | **x***** | **x***** | **x***** | **x***** | **x***** | **x***** | **x***** | **x***** | **x***** | **x***** | **x***** | **x***** | **x***** | **x***** | **x***** | **x***** | |  | | **-0.15** | | **-0.23 to -0.06** |  | **0.86** | **0.001** |
| Migration strategy (ref: sedentary) | | x | x | x | x | x | x | x | x | x | x | x | x | x | x | x | x | x | x | x | |  | |  | |  | 1.00 |  |  |
| Partial |  | x | x | x | x | x | x | x | x | x | x | x | x | x | x | x | x | x | x | x | |  | | 0.05 | | -0.05 to 0.15 |  | 1.05 | 0.351 |
| Short-distance |  | x* | x | x | x | x | x* | x | x | x* | x | x | x* | x* | x | x* | x | x | x | x | |  | | 0.11 | | -0.01 to 0.22 |  | 1.11 | 0.063 |
| Long-distance |  | x | x | x | x | x | x | x | x | x | x | x | x | x | x | x | x | x | x | x | |  | | -0.08 | | -0.18 to 0.02 |  | 0.93 | 0.127 |
| Historical range size | | x | x* | x* |  | x* | x | x | x* |  |  |  | x | x | x** |  | x | x* | x* | x* | |  | | -0.03 | | -0.08 to 0.02 | 0.75 | 0.97 | 0.217 |
| Natal dispersal | | x* |  |  | x* | x | x | x* | x | x* | x* | x* | x* | x* |  | x* |  |  |  |  | |  | | 0.03 | | -0.03 to 0.08 | 0.64 | 1.03 | 0.322 |
| Lifespan | |  | x* | x |  | x |  |  | x |  |  |  |  |  | x* |  | x* | x* | x* | x* | |  | | 0.02 | | -0.03 to 0.07 | 0.47 | 1.02 | 0.455 |
| Diet breadth | |  | x |  |  |  | x |  | x |  | x |  |  |  | x |  | x | x | x |  | |  | | -0.01 | | -0.04 to 0.02 | 0.40 | 0.99 | 0.552 |
| Thermal max | |  |  |  | x |  |  | x |  |  | x | x |  |  |  |  | x |  |  |  | |  | | -0.01 | | -0.04 to 0.03 | 0.25 | 0.99 | 0.667 |
| Habitat breadth | |  |  |  |  |  |  |  |  |  |  | x | x |  |  |  |  | x |  | x | |  | | -0.003 | | -0.03 to 0.02 | 0.17 | 1.00 | 0.781 |
| Clutch size | |  |  |  |  |  |  |  |  |  |  |  |  | x |  |  |  |  | x |  | |  | | 0.001 | | -0.01 to 0.01 | 0.08 | 1.00 | 0.866 |
| Body mass | |  |  |  |  |  |  |  |  |  |  |  |  |  | x |  |  |  |  |  | |  | | -0.001 | | -0.01 to 0.01 | 0.04 | 1.00 | 0.897 |
| Thermal range | |  |  |  |  |  |  |  |  |  |  |  |  |  |  | x |  |  |  |  | |  | | -0.001 | | -0.01 to 0.01 | 0.04 | 1.00 | 0.870 |
| Diet type | |  |  |  |  |  |  |  |  |  |  |  |  |  |  |  |  |  |  |  | |  | | - | | - | - | - | - |
|  |  |  |  |  |  |  |  |  |  |  |  |  |  |  |  |  |  |  |  |  | |  | |  | |  |  |  |  |
| Δ_i_ | | 0.00 | 0.09 | 0.38 | 0.60 | 0.85 | 1.08 | 1.31 | 1.33 | 1.37 | 1.41 | 1.44 | 1.46 | 1.53 | 1.59 | 1.66 | 1.68 | 1.69 | 1.80 | 1.89 | |  | |  | |  |  |  |  |
| w_i_ | | 0.093 | 0.089 | 0.077 | 0.069 | 0.061 | 0.054 | 0.048 | 0.048 | 0.047 | 0.046 | 0.045 | 0.045 | 0.043 | 0.042 | 0.041 | 0.040 | 0.040 | 0.038 | 0.036 | |  | |  | |  |  |  |  |
| marginal R^2^ | | 0.287 | 0.291 | 0.288 | 0.286 | 0.289 | 0.288 | 0.288 | 0.292 | 0.281 | 0.288 | 0.288 | 0.288 | 0.288 | 0.292 | 0.284 | 0.292 | 0.292 | 0.291 | 0.289 | |  | |  | |  |  |  |  |
|  |  |  |  |  |  |  |  |  |  |  |  |  |  |  |  |  |  |  |  |  | |  | |  | |  |  |  |  |

Sign. codes: * p<0.05, ** p<0.01, *** p<0.001

**Table S4**

|  | | | | | | | | | | | | | | | | | | |
| --- | --- | --- | --- | --- | --- | --- | --- | --- | --- | --- | --- | --- | --- | --- | --- | --- | --- | --- |
|  |  |  |  |  |  |  |  |  |  |  |  |  |  |  |  |  |  |  |
|  |  | lmm - Model rank | | | | | | | | | |  |  | Model average | | | |  |
|  |  | 1 | 2 | 3 | 4 | 5 | 6 | 7 | 8 | 9 | 10 | 11 |  | β | 95% CI | w_sum_ | exp β | p |
| Intercept | | **x** | **x** | **x** | **x** | **x** | **x** | **x** | **x** | **x** | **x** | **x** |  | 1.26 | -0.77 to 3.30 |  | 3.53 | 0.244 |
| Habitat type (ref: Habitat generalist) | | **x** | **x** | **x** | **x** | **x** | **x** | **x** | **x** | **x** | **x** | **x** |  |  |  | **1.00** |  |  |
| **Agriculture & Grasslands** |  | **x*** | **x*** | **x** | **x*** | **x** | **x** | **x*** | **x*** | **x*** | **x*** | **x*** |  | **-2.81** | **-5.57 to -0.04** |  | **0.06** | **0.047** |
| Forest |  | x | x | x | x | x | x | x | x | x | x | x |  | -0.46 | -2.76 to 1.83 |  | 0.63 | 0.693 |
| Inland wetlands |  | x | x | x | x | x | x | x | x | x | x | x |  | -1.71 | -4.02 to 0.59 |  | 0.18 | 0.145 |
| **Tundra-Mire-Moor** |  | **x***** | **x***** | **x***** | **x***** | **x***** | **x***** | **x***** | **x***** | **x***** | **x***** | **x***** |  | **-5.53** | **-8.15 to -2.91** |  | **0.004** | **<0.001** |
| Wood & Shrubland |  | x | x | x | x | x | x | x | x | x | x | x |  | -1.34 | -3.45 to 0.78 |  | 0.26 | 0.216 |
| Other |  | x | x | x | x | x | x | x | x | x | x | x |  | -0.54 | -3.36 to 2.29 |  | 0.59 | 0.710 |
| **Region (ref: Central EU)** | | **x***** | **x***** | **x***** | **x***** | **x***** | **x***** | **x***** | **x***** | **x***** | **x***** | **x***** |  |  |  | **1.00** |  |  |
| **Fennoscandia** |  | **x***** | **x***** | **x***** | **x***** | **x***** | **x***** | **x***** | **x***** | **x***** | **x***** | **x***** |  | **5.85** | **5.06 to 6.63** |  | **346.19** | **<0.001** |
| **North-western EU** |  | **x***** | **x***** | **x***** | **x***** | **x***** | **x***** | **x***** | **x***** | **x***** | **x***** | **x***** |  | **-3.54** | **-4.47 to -2.60** |  | **0.03** | **<0.001** |
| **Clutch size** | | **x*** | **x*** | **x** | **x*** | **x*** | **x*** | **x*** | **x*** | **x*** | **x*** | **x*** |  | **0.47** | **0.04 to 0.91** | **1.00** | **1.61** | **0.034** |
| **Natal dispersal** | | **x**** | **x**** | **x**** | **x***** | **x*** | **x**** | **x**** | **x*** | **x**** | **x**** | **x**** |  | **0.69** | **0.22 to 1.16** | **1.00** | **1.99** | **0.004** |
| Habitat breadth | | x | x |  | x |  |  | x | x | x | x | x |  | -0.29 | -0.85 to 0.26 | 0.71 | 0.75 | 0.302 |
| Lifespan | |  | x |  |  | x |  |  | x |  |  |  |  | 0.08 | -0.27 to 0.44 | 0.28 | 1.09 | 0.638 |
| Migration strategy (ref: Sedentary) | |  |  |  | x |  | x |  |  |  |  |  |  |  |  | 0.18 |  |  |
| Partial |  |  |  |  | x |  | x |  |  |  |  |  |  | 0.06 | -0.47 to 0.59 |  | 1.06 | 0.827 |
| Short-distance |  |  |  |  | x |  | x |  |  |  |  |  |  | 0.11 | -0.59 to 0.81 |  | 1.12 | 0.758 |
| Long-distance |  |  |  |  | x |  | x |  |  |  |  |  |  | -0.10 | -0.74 to 0.53 |  | 0.90 | 0.746 |
| Diet breadth | |  |  |  |  |  |  |  | x | x |  |  |  | -0.02 | -0.20 to 0.16 | 0.13 | 0.98 | 0.824 |
| Thermal range | |  |  |  |  |  |  | x |  |  |  |  |  | 0.01 | -0.12 to 0.15 | 0.07 | 1.01 | 0.862 |
| Body mass | |  |  |  |  |  |  |  |  |  | x |  |  | 0.01 | -0.09 to 0.11 | 0.06 | 1.01 | 0.914 |
| IUCN status: Threatened | |  |  |  |  |  |  |  |  |  |  | x |  | -0.02 | -0.40 to 0.36 | 0.06 | 0.98 | 0.915 |
| Thermal max | |  |  |  |  |  |  |  |  |  |  |  |  | - | - | - | - | - |
| Historical range size | |  |  |  |  |  |  |  |  |  |  |  |  | - | - | - | - | - |
| Diet type | |  |  |  |  |  |  |  |  |  |  |  |  | - | - | - | - | - |
| - |  |  |  |  |  |  |  |  |  |  |  |  |  |  |  |  |  |  |
| Δ_i_ | | 0.00 | 0.33 | 0.39 | 0.88 | 1.17 | 1.29 | 1.50 | 1.67 | 1.80 | 1.92 | 1.93 |  |  |  |  |  |  |
| w_i_ | | 0.154 | 0.131 | 0.127 | 0.099 | 0.086 | 0.081 | 0.073 | 0.067 | 0.063 | 0.059 | 0.059 |  |  |  |  |  |  |
| marginal R^2^ | | 0.530 | 0.533 | 0.527 | 0.537 | 0.529 | 0.534 | 0.531 | 0.534 | 0.531 | 0.531 | 0.531 |  |  |  |  |  |  |
|  |  |  |  |  |  |  |  |  |  |  |  |  |  |  |  |  |  |  |

Sign. codes: * p<0.05, ** p<0.01, *** p<0.001

**Table S5**

|  | |  | | | | | | | | | |  |  | |  |  |
| --- | --- | --- | --- | --- | --- | --- | --- | --- | --- | --- | --- | --- | --- | --- | --- | --- |
|  | | lm - Model rank | | | | | | | | | |  | Model average | |  |  |
|  |  | 1 | 2 | 3 | 4 | 5 | 6 | 7 | 8 | 9 | 10 |  | β | 95% CI | w_sum_ | p |
| **Intercept** | | **x*** | **x*** | **x*** | **x*** | **x*** | **x*** | **x*** | **x*** | **x*** | **x*** |  | **0.62** | **0.13 to 1.12** | - | **0.014** |
| **Diet breadth** | | **x*** | **x*** | **x*** | **x*** | **x*** | **x*** | **x*** | **x*** | **x*** | **x*** |  | **-0.60** | **-1.10 to -0.09** | **1.00** | **0.020** |
| **IUCN status: Threatened** | | **x*** | **x*** | **x**** | **x**** | **x*** | **x*** | **x**** | **x*** | **x*** | **x*** |  | **2.80** | **0.58 to 5.02** | **1.00** | **0.013** |
| Habitat breadth | | x |  | x |  | x | x |  |  |  | x |  | -0.22 | -0.76 to 0.32 | 0.55 | 0.422 |
| Natal dispersal | |  |  | x | x |  |  | x |  |  |  |  | -0.09 | -0.49 to 0.32 | 0.28 | 0.665 |
| Historical range size | |  |  |  |  | x |  | x | x |  |  |  | -0.06 | -0.40 to 0.28 | 0.25 | 0.710 |
| Thermal max | |  |  |  |  |  | x |  |  | x |  |  | -0.03 | -0.29 to 0.22 | 0.16 | 0.792 |
| Historical northern limit | |  |  |  |  |  |  |  |  |  | x |  | 0.01 | -0.14 to 0.15 | 0.07 | 0.917 |
| Body mass | |  |  |  |  |  |  |  |  |  |  |  | - | - | - | - |
| Clutch size | |  |  |  |  |  |  |  |  |  |  |  | - | - | - | - |
| Lifespan | |  |  |  |  |  |  |  |  |  |  |  | - | - | - | - |
| Migration strategy | |  |  |  |  |  |  |  |  |  |  |  | - | - | - | - |
| Diet type | |  |  |  |  |  |  |  |  |  |  |  | - | - | - | - |
|  |  |  |  |  |  |  |  |  |  |  |  |  |  |  |  |  |
|  | Δ_i_ | 0.00 | 0.81 | 1.11 | 1.63 | 1.65 | 1.68 | 1.71 | 1.80 | 1.81 | 1.94 |  |  |  |  |  |
|  | w_i_ | 0.193 | 0.129 | 0.110 | 0.085 | 0.085 | 0.084 | 0.082 | 0.079 | 0.078 | 0.073 |  |  |  |  |  |
|  | adj. R^2^ | 0.061 | 0.052 | 0.061 | 0.053 | 0.058 | 0.058 | 0.058 | 0.053 | 0.053 | 0.057 |  |  |  |  |  |
|  |  |  |  |  |  |  |  |  |  |  |  |  |  |  |  |  |

Sign. codes: * p<0.05, ** p<0.01, *** p<0.001

|  | | |  |  |  |  |  |  |  |  |  |  |  |  |  |  |  |  |  |
| --- | --- | --- | --- | --- | --- | --- | --- | --- | --- | --- | --- | --- | --- | --- | --- | --- | --- | --- | --- |
|  | | | lm - Model rank | | | | | | | | | | |  | Model average | |  |  |  |
|  |  | | 1 | 2 | 3 | 4 | 5 | 6 | 7 | 8 | 9 | 10 | 11 |  | β | 95% CI | w_sum_ | P | expβ |
| **Intercept** | | | **x***** | **x***** | **x***** | **x***** | **x***** | **x***** | **x***** | **x***** | **x***** | **x***** | **x***** |  | **0.41** | **0.12 to 0.71** | - | **0.006** | **1.51** |
| **Diet breadth** | | | **x*** | **x*** | **x*** | **x*** | **x*** | **x*** | **x*** | **x*** | **x*** | **x**** | **x*** |  | **0.13** | **0.01 to 0.24** | **1.00** | **0.027** | **1.14** |
| **Historical northern limit** | | | **x**** | **x**** | **x**** | **x** | **x**** | **x**** | **x**** | **x**** | **x**** | **x**** | **x**** |  | **-0.18** | **-0.31 to -0.05** | **1.00** | **0.007** | **0.84** |
| **Historical range size** | | | **x*** | **x** | **x*** | **x*** | **x*** | **x*** | **x*** | **x** | **x*** | **x*** | **x*** |  | **-0.15** | **-0.29 to -0.01** | **1.00** | **0.040** | **0.86** |
| Migration strategy (ref: Sedentary) | | | x |  | x | x | x | x | x |  | x | x |  |  |  |  | 0.74 |  |  |
|  | Partial |  | **x*** |  | **x*** | **x*** | **x*** | **x*** | **x*** |  | **x*** | **x*** |  |  | -0.27 | -0.70 to 0.16 |  | 0.219 | 0.76 |
|  | Short-distance |  | **x*** |  | **x*** | **x*** | **x*** | **x*** | **x*** |  | **x*** | **x*** |  |  | -0.30 | -0.75 to 0.16 |  | 0.203 | 0.74 |
|  | Long-distance |  | x |  | x | x | x | x | x |  | x | x |  |  | -0.12 | -0.42 to 0.19 |  | 0.463 | 0.89 |
| Lifespan | | |  |  | x | x |  |  | x |  |  |  |  |  | 0.03 | -0.08 to 0.13 | 0.28 | 0.625 | 1.03 |
| Body mass | | |  |  |  | x |  | x |  | x |  |  |  |  | -0.01 | -0.09 to 0.06 | 0.22 | 0.725 | 0.99 |
| Thermal max | | |  |  |  |  | x |  |  |  |  |  | x |  | 0.01 | -0.09 to 0.12 | 0.16 | 0.796 | 1.01 |
| Clutch size | | |  |  |  |  |  |  | x |  |  | x |  |  | 0.01 | -0.05 to 0.06 | 0.13 | 0.823 | 1.01 |
| IUCN status: Threatened | | |  |  |  |  |  |  |  |  | x |  |  |  | 0.01 | -0.10 to 0.12 | 0.07 | 0.888 | 1.01 |
| Natal dispersal | | |  |  |  |  |  |  |  |  |  |  |  |  | - | - | - | - | -  -  - |
| Habitat breadth | | |  |  |  |  |  |  |  |  |  |  |  |  | - | - | - | - | -  - |
| Diet type | | |  |  |  |  |  |  |  |  |  |  |  |  | - | - | - | - | - |
|  |  | |  |  |  |  |  |  |  |  |  |  |  |  |  |  |  |  |  |
|  | Δ_i_ | | 0.00 | 0.50 | 0.52 | 1.14 | 1.34 | 1.69 | 1.70 | 1.74 | 1.77 | 1.90 | 1.93 |  |  |  |  |  |  |
|  | w_i_ | | 0.164 | 0.128 | 0.127 | 0.093 | 0.084 | 0.071 | 0.070 | 0.069 | 0.068 | 0.063 | 0.063 |  |  |  |  |  |  |
|  | adj. R^2^ | | 0.076 | 0.059 | 0.078 | 0.080 | 0.075 | 0.073 | 0.078 | 0.058 | 0.073 | 0.072 | 0.057 |  |  |  |  |  |  |
|  |  | |  |  |  |  |  |  |  |  |  |  |  |  |  |  |  |  |  |

**Table S6**

Sign. codes: * p<0.05, ** p<0.01, *** p<0.001

**Table S7**

|  |  |  |  |  |  |  |  |  |  |  |  |  |  |  |
| --- | --- | --- | --- | --- | --- | --- | --- | --- | --- | --- | --- | --- | --- | --- |
|  |  |  | lm - Model rank | | | | | | |  | Model average | |  |  |
|  |  |  | 1 | 2 | 3 | 4 | 5 | 6 | 7 |  | β | 95% CI | w_sum_ | p |
|  | Intercept | | **x** | **x** | **x** | **x** | **x** | **x** | **x*** |  | 1.58 | -2.49 to 5.62 | - | 0.437 |
|  | **IUCN status: Threatened** | | **x*** | **x*** | **x*** | **x** | **x** | **x*** | **x*** |  | **-3.25** | **-6.30 to -0.19** | **1.00** | **0.038** |
|  | **Natal dispersal** | | **x*** | **x*** | **x*** | **x*** | **x*** | **x*** | **x*** |  | **1.45** | **0.26 to 2.65** | **1.00** | **0.017** |
|  | Diet type (ref: Omnivore) | | x | x | x | x | x | x | x |  |  |  | 1.00 |  |
|  | Herbivore |  | x | **x*** | **x*** | **x** | **x*** | x | x |  | 4.23 | -0.57 to 9.03 |  | 0.084 |
|  | Invertivore |  | x | x | x | x | x | x | x |  | -0.42 | -5.14 to 4.30 |  | 0.861 |
|  | Omni-carnivore |  | x | x | x | x | x | x | x |  | -0.27 | -5.31 to 4.76 |  | 0.915 |
|  | Clutch size | | x* | x* | x* | x* | x* | x* |  |  | 1.25 | -0.18 to 2.52 | 0.91 | 0.090 |
|  | Habitat breadth | | x |  | x |  |  | x | x |  | -0.43 | -1.47 to 0.62 | 0.56 | 0.424 |
|  | Diet breadth | |  |  | x | x |  |  |  |  | 0.25 | -0.98 to 1.47 | 0.25 | 0.694 |
|  | Body mass | |  |  |  |  | x | x |  |  | -0.14 | -0.96 to 0.67 | 0.22 | 0.729 |
|  | Lifespan | |  |  |  |  |  |  |  |  | - | - | - | - |
|  | Migration strategy | |  |  |  |  |  |  |  |  | - | - | - | - |
|  | Thermal max | |  |  |  |  |  |  |  |  | - | - | - | - |
|  | Historical range size | |  |  |  |  |  |  |  |  | - | - | - | - |
|  | Historical northern limit | |  |  |  |  |  |  |  |  | - | - | - | - |
|  | Region | |  |  |  |  |  |  |  |  | - | - | - | - |
|  |  |  |  |  |  |  |  |  |  |  |  |  |  |  |
|  | Δ_i_ | | 0.00 | 0.08 | 0.095 | 1.28 | 1.31 | 1.38 | 1.78 |  |  |  |  |  |
|  | w_i_ | | 0.220 | 0.212 | 0.137 | 0.116 | 0.115 | 0.110 | 0.090 |  |  |  |  |  |
|  | adj. R^2^ | | 0.076 | 0.068 | 0.077 | 0.068 | 0.068 | 0.075 | 0.057 |  |  |  |  |  |
|  |  |  |  |  |  |  |  |  |  |  |  |  |  |  |
|  |  |  |  |  |  |  |  |  |  |  |  |  |  |  |

Sign. codes: * p<0.05, ** p<0.01, *** p<0.001
