# Supplementary material for: The Role of Intrinsic Factors in Explaining Range Shifts of European Breeding Birds: A Meta‐Analysis
Source: Ecol Evol. 2025 Apr 21;15(4):e71308. doi: 10.1002/ece3.71308 (PMC12012262; doi:10.1002/ece3.71308)
Supplement: Supplementary file 1 — Data S1. [file ECE3-15-e71308-s001.zip › Warmer et al._Appx4.docx]

**Appendix 4 – Comparison between full models**

Comparison between full models (lowest AICc values) including both study and species as random factor and those including only species as random factor.

###### Relative change (D): both study (authors) and species (eng_name) included as random factors

Linear mixed model fit by maximum likelihood . t-tests use Satterthwaite's method ['lmerModLmerTest']

Formula: (sign(D) * log(abs(D) + 1)) ~ IUCN + H_type + D_type + H_breadth_sum +

D_breadth_sum + migration + body_mass + clutch_size + lifespan +

natal_dispersal + thermal_max + thermal_range + hist_range + region + (1 | eng_name) + (1 | authors)

Data: D_glmm

AIC BIC logLik deviance df.resid

422.7 555.7 -180.3 360.7 509

Scaled residuals:

Min 1Q Median 3Q Max

-3.4438 -0.4382 0.0017 0.4248 3.5132

Random effects:

Groups Name Variance Std.Dev.

eng_name (Intercept) 0.01299 0.1140

authors (Intercept) 0.02211 0.1487

Residual 0.09878 0.3143

Number of obs: 540, groups: eng_name, 226; authors, 14

Fixed effects:

Estimate Std. Error df t value Pr(>|t|)

(Intercept) 0.475412 0.134906 30.233639 3.524 0.001375 **

IUCNThreatened -0.241266 0.065586 183.731030 -3.679 0.000308 ***

H_typeAgriculture_Grasslands -0.496723 0.119776 131.937395 -4.147 5.99e-05 ***

H_typeForest -0.385462 0.101487 147.476401 -3.798 0.000213 ***

H_typeInland wetlands -0.356610 0.099827 149.912628 -3.572 0.000476 ***

H_typeTundraMireMoor -0.903473 0.134045 220.106237 -6.740 1.37e-10 ***

H_typeWood_Shrubland -0.392817 0.093572 152.016127 -4.198 4.57e-05 ***

H_typeOther -0.454154 0.137580 200.820139 -3.301 0.001140 **

D_typeHerbi-invertivore -0.124121 0.098888 123.327166 -1.255 0.211793

D_typeHerbivore 0.024020 0.073091 122.800855 0.329 0.742997

D_typeInvertivore -0.081253 0.058727 128.104349 -1.384 0.168902

D_typeOmnicarnivore 0.044611 0.108686 167.513200 0.410 0.681992

D_typeOmnivore -0.005567 0.125499 117.316275 -0.044 0.964691

D_typePiscivore -0.102579 0.115480 177.069082 -0.888 0.375593

H_breadth_sum -0.028052 0.022715 128.501917 -1.235 0.219100

D_breadth_sum -0.051693 0.030868 134.144652 -1.675 0.096342 .

migrationMixed 0.060559 0.049368 97.702406 1.227 0.222890

migrationShort-distance 0.102782 0.058375 144.910279 1.761 0.080394 .

migrationLong-distance -0.078930 0.050482 112.688860 -1.564 0.120736

body_mass -0.030368 0.018569 267.161343 -1.635 0.103142

clutch_size 0.022373 0.022061 133.140009 1.014 0.312358

lifespan 0.029038 0.026584 140.039054 1.092 0.276570

natal_dispersal 0.041523 0.024696 138.283014 1.681 0.094948 .

thermal_max -0.017026 0.025503 264.527850 -0.668 0.504966

thermal_range -0.008181 0.025238 197.383687 -0.324 0.746179

hist_range -0.045073 0.029780 182.536368 -1.514 0.131871

regionFennoscandia 0.228669 0.159041 6.378558 1.438 0.197683

regionNorthwestern Europe -0.323066 0.124024 7.959547 -2.605 0.031513 *

---

R2m R2c

[1,] 0.3125525 0.4927934

##### Relative change (D): only species (eng_name) included as random factor

Linear mixed model fit by maximum likelihood . t-tests use Satterthwaite's method ['lmerModLmerTest']

Formula: (sign(D) * log(abs(D) + 1)) ~ IUCN + H_type + D_type + H_breadth_sum +

D_breadth_sum + migration + body_mass + clutch_size + lifespan +

natal_dispersal + thermal_max + thermal_range + hist_range + region + (1 | eng_name)

Data: D_glmm

AIC BIC logLik deviance df.resid

434.1 562.8 -187.0 374.1 510

Scaled residuals:

Min 1Q Median 3Q Max

-3.3064 -0.4902 -0.0017 0.4889 3.3302

Random effects:

Groups Name Variance Std.Dev.

eng_name (Intercept) 0.01368 0.1170

Residual 0.10476 0.3237

Number of obs: 540, groups: eng_name, 226

Fixed effects:

Estimate Std. Error df t value Pr(>|t|)

(Intercept) 4.748e-01 1.067e-01 1.519e+02 4.449 1.66e-05 ***

IUCNThreatened -2.085e-01 6.713e-02 1.892e+02 -3.106 0.002192 **

H_typeAgriculture_Grasslands -4.985e-01 1.231e-01 1.390e+02 -4.051 8.45e-05 ***

H_typeForest -3.679e-01 1.041e-01 1.542e+02 -3.533 0.000542 ***

H_typeInland wetlands -3.382e-01 1.023e-01 1.565e+02 -3.306 0.001174 **

H_typeTundraMireMoor -9.117e-01 1.355e-01 2.245e+02 -6.731 1.39e-10 ***

H_typeWood_Shrubland -3.991e-01 9.617e-02 1.599e+02 -4.150 5.39e-05 ***

H_typeOther -4.764e-01 1.414e-01 2.105e+02 -3.370 0.000894 ***

D_typeHerbi-invertivore -1.229e-01 1.011e-01 1.290e+02 -1.216 0.226207

D_typeHerbivore 1.763e-02 7.504e-02 1.282e+02 0.235 0.814673

D_typeInvertivore -8.672e-02 6.016e-02 1.332e+02 -1.442 0.151753

D_typeOmnicarnivore 5.856e-02 1.116e-01 1.745e+02 0.525 0.600300

D_typeOmnivore -3.805e-02 1.287e-01 1.218e+02 -0.296 0.767962

D_typePiscivore -8.533e-02 1.186e-01 1.860e+02 -0.720 0.472701

H_breadth_sum -2.523e-02 2.328e-02 1.338e+02 -1.084 0.280355

D_breadth_sum -4.747e-02 3.165e-02 1.406e+02 -1.500 0.135830

migrationMixed 5.205e-02 5.064e-02 1.021e+02 1.028 0.306499

migrationShort-distance 8.546e-02 5.962e-02 1.500e+02 1.433 0.153807

migrationLong-distance -8.544e-02 5.182e-02 1.180e+02 -1.649 0.101857

body_mass -2.831e-02 1.904e-02 2.765e+02 -1.487 0.138152

clutch_size 2.335e-02 2.263e-02 1.387e+02 1.032 0.303914

lifespan 3.214e-02 2.721e-02 1.468e+02 1.181 0.239354

natal_dispersal 3.376e-02 2.518e-02 1.430e+02 1.341 0.182146

thermal_max -2.781e-02 2.485e-02 2.780e+02 -1.119 0.264045

thermal_range 2.463e-04 2.552e-02 2.049e+02 0.010 0.992310

hist_range -2.590e-02 2.963e-02 1.864e+02 -0.874 0.383139

regionFennoscandia 3.118e-01 3.655e-02 3.624e+02 8.532 3.98e-16 ***

regionNorthwestern Europe -1.477e-01 4.241e-02 3.367e+02 -3.482 0.000564 ***

---

R2m R2c

[1,] 0.3102611 0.3899435

##### Rate-of-change (km2/year):both study (authors) and species (eng_name) included as random factors

Linear mixed model fit by maximum likelihood . t-tests use Satterthwaite's method ['lmerModLmerTest']

Formula: (sign(km2.year) * log(abs(km2.year) + 1)) ~ H_type + D_type +

H_breadth_sum + D_breadth_sum + migration + IUCN + body_mass + clutch_size + lifespan + natal_dispersal + thermal_max +

thermal_range + hist_range + region + (1 | eng_name) + (1 | authors)

Data: km2_glmm

AIC BIC logLik deviance df.resid

2129.0 2251.9 -1033.5 2067.0 358

Scaled residuals:

Min 1Q Median 3Q Max

-3.6973 -0.4717 0.0391 0.4748 3.2641

Random effects:

Groups Name Variance Std.Dev.

eng_name (Intercept) 0.9595 0.9795

authors (Intercept) 0.8443 0.9189

Residual 10.7025 3.2715

Number of obs: 389, groups: eng_name, 220; authors, 13

Fixed effects:

Estimate Std. Error df t value Pr(>|t|)

(Intercept) 2.17791 1.41808 33.93869 1.536 0.133854

H_typeAgriculture_Grasslands -2.81504 1.38628 212.87321 -2.031 0.043535 *

H_typeForest -1.10051 1.18267 239.39914 -0.931 0.353036

H_typeInland wetlands -2.23421 1.16439 239.99777 -1.919 0.056198 .

H_typeTundraMireMoor -5.78856 1.49355 295.05142 -3.876 0.000131 ***

H_typeWood_Shrubland -1.60171 1.10035 245.44487 -1.456 0.146771

H_typeOther -1.28969 1.61275 253.17785 -0.800 0.424644

D_typeHerbi-invertivore -0.23650 1.10672 182.82452 -0.214 0.831020

D_typeHerbivore 0.20381 0.82858 184.62186 0.246 0.805971

D_typeInvertivore -0.39280 0.66987 198.10463 -0.586 0.558288

D_typeOmnicarnivore 1.73419 1.24859 258.83954 1.389 0.166051

D_typeOmnivore 1.27454 1.40773 177.93074 0.905 0.366485

D_typePiscivore 0.07299 1.35620 236.51275 0.054 0.957124

H_breadth_sum -0.44430 0.25077 199.51033 -1.772 0.077962 .

D_breadth_sum -0.61983 0.34417 205.13548 -1.801 0.073179 .

migrationMixed 0.32167 0.55956 152.19347 0.575 0.566237

migrationShort-distance 0.53853 0.67831 220.63805 0.794 0.428085

migrationLong-distance -0.61851 0.57940 181.96023 -1.068 0.287160

IUCNThreatened -0.73456 0.73397 257.68506 -1.001 0.317861

body_mass -0.09327 0.21102 358.48304 -0.442 0.658753

clutch_size 0.42247 0.25188 202.66815 1.677 0.095031 .

lifespan 0.06991 0.30545 219.24697 0.229 0.819185

natal_dispersal 0.68353 0.28185 209.81322 2.425 0.016148 *

thermal_max -0.10254 0.30717 293.60867 -0.334 0.738742

thermal_range 0.17058 0.30737 296.29504 0.555 0.579347

hist_range -0.03659 0.35780 263.56035 -0.102 0.918633

regionFennoscandia 4.87689 1.19099 2.66844 4.095 0.032840 *

regionNorthwestern Europe -4.53887 1.01643 3.74979 -4.466 0.012861 *

---

R2m R2c

[1,] 0.516228 0.5860024

##### Rate-of-change (km2/year): only species (eng_name) included as random factor

Linear mixed model fit by maximum likelihood . t-tests use Satterthwaite's method ['lmerModLmerTest']

Formula: (sign(km2.year) * log(abs(km2.year) + 1)) ~ H_type + D_type +

H_breadth_sum + D_breadth_sum + migration + IUCN + body_mass + clutch_size + lifespan + natal_dispersal + thermal_max +

thermal_range + hist_range + region + (1 | eng_name)

Data: km2_glmm

AIC BIC logLik deviance df.resid

2128.7 2247.6 -1034.4 2068.7 359

Scaled residuals:

Min 1Q Median 3Q Max

-3.6424 -0.4791 0.0280 0.4645 3.3592

Random effects:

Groups Name Variance Std.Dev.

eng_name (Intercept) 1.009 1.004

Residual 10.982 3.314

Number of obs: 389, groups: eng_name, 220

Fixed effects:

Estimate Std. Error df t value Pr(>|t|)

(Intercept) 1.74993 1.24336 248.08345 1.407 0.160554

H_typeAgriculture_Grasslands -2.91499 1.40556 219.31859 -2.074 0.039257 *

H_typeForest -1.00966 1.19748 244.89550 -0.843 0.399968

H_typeInland wetlands -2.21855 1.17833 245.31814 -1.883 0.060913 .

H_typeTundraMireMoor -5.82742 1.49945 294.43334 -3.886 0.000126 ***

H_typeWood_Shrubland -1.62615 1.11547 252.76381 -1.458 0.146133

H_typeOther -1.33564 1.63314 259.33986 -0.818 0.414200

D_typeHerbi-invertivore -0.28229 1.11958 186.11769 -0.252 0.801211

D_typeHerbivore 0.12030 0.83974 188.99489 0.143 0.886241

D_typeInvertivore -0.36930 0.67753 201.03421 -0.545 0.586311

D_typeOmnicarnivore 1.83693 1.26422 263.86443 1.453 0.147409

D_typeOmnivore 0.99687 1.42459 180.52207 0.700 0.484979

D_typePiscivore 0.09580 1.37473 243.04911 0.070 0.944500

H_breadth_sum -0.43785 0.25383 202.82501 -1.725 0.086056 .

D_breadth_sum -0.55664 0.34819 209.87040 -1.599 0.111406

migrationMixed 0.31065 0.56699 155.18243 0.548 0.584558

migrationShort-distance 0.52638 0.68371 221.42175 0.770 0.442188

migrationLong-distance -0.65123 0.58698 185.64539 -1.109 0.268666

IUCNThreatened -0.54908 0.74015 258.26528 -0.742 0.458851

body_mass -0.05555 0.21301 363.22079 -0.261 0.794419

clutch_size 0.43548 0.25498 206.19099 1.708 0.089162 .

lifespan 0.06051 0.30873 222.83989 0.196 0.844789

natal_dispersal 0.67360 0.28431 212.46859 2.369 0.018720 *

thermal_max -0.03246 0.29612 323.97455 -0.110 0.912783

thermal_range 0.15235 0.30701 295.67209 0.496 0.620100

hist_range -0.02550 0.35244 266.25618 -0.072 0.942382

regionFennoscandia 5.86549 0.42822 272.30940 13.697 < 2e-16 ***

regionNorthwestern Europe -3.52858 0.47991 263.16963 -7.353 2.46e-12 ***

---

R2m R2c

[1,] 0.53964 0.5783645

##### Change-type (shift): both study (authors) and species (eng_name) included as random factors

Generalized linear mixed model fit by maximum likelihood (Laplace Approximation) ['glmerMod']

Family: binomial ( logit )

Formula: shift_type ~ H_type + H_breadth_sum + D_breadth_sum + D_type +

migration + IUCN + body_mass + clutch_size + lifespan + natal_dispersal +

thermal_max + thermal_range + hist_range + region + (1 | eng_name) + (1 | authors)

Data: EC_glmm

Control: glmerControl(optimizer = "bobyqa", optCtrl = list(maxfun = 1e+06))

AIC BIC logLik deviance df.resid

649.6 787.7 -293.8 587.6 605

Scaled residuals:

Min 1Q Median 3Q Max

-5.4171 -0.4907 0.2429 0.5374 3.9153

Random effects:

Groups Name Variance Std.Dev.

eng_name (Intercept) 0.2251 0.4745

authors (Intercept) 0.4970 0.7050

Number of obs: 636, groups: eng_name, 244; authors, 33

Fixed effects:

Estimate Std. Error z value Pr(>|z|)

(Intercept) 1.875028 0.864326 2.169 0.030056 *

H_typeAgriculture_Grasslands -2.756427 0.892365 -3.089 0.002009 **

H_typeForest -1.178894 0.757875 -1.556 0.119821

H_typeInland wetlands -1.877053 0.752730 -2.494 0.012643 *

H_typeTundraMireMoor -3.394515 0.995339 -3.410 0.000649 ***

H_typeWood_Shrubland -1.677300 0.704018 -2.382 0.017197 *

H_typeOther -1.234079 1.039107 -1.188 0.234978

H_breadth_sum -0.220775 0.159468 -1.384 0.166222

D_breadth_sum -0.511744 0.224515 -2.279 0.022647 *

D_typeHerbi-invertivore -0.263563 0.740272 -0.356 0.721814

D_typeHerbivore 0.809563 0.564116 1.435 0.151259

D_typeInvertivore -0.003148 0.435669 -0.007 0.994235

D_typeOmnicarnivore 2.081062 0.901737 2.308 0.021008 *

D_typeOmnivore 1.345269 0.906664 1.484 0.137873

D_typePiscivore 1.002257 0.881472 1.137 0.255528

migrationMixed 0.608003 0.362220 1.679 0.093240 .

migrationShort-distance 0.563335 0.424801 1.326 0.184802

migrationLong-distance 0.000653 0.368431 0.002 0.998586

IUCNThreatened -1.507915 0.462095 -3.263 0.001102 **

body_mass -0.301326 0.141363 -2.132 0.033042 *

clutch_size 0.096796 0.167976 0.576 0.564447

lifespan 0.367262 0.205180 1.790 0.073461 .

natal_dispersal 0.026124 0.192411 0.136 0.892003

thermal_max -0.139338 0.195051 -0.714 0.475001

thermal_range 0.062076 0.185572 0.335 0.737994

hist_range -0.167686 0.217092 -0.772 0.439867

regionFennoscandia 1.557543 0.752819 2.069 0.038551 *

regionIberian peninsula 1.711833 0.790288 2.166 0.030304 *

regionNorthwestern Europe -2.245478 0.628117 -3.575 0.000350 ***

---

R2m R2c

theoretical 0.4141208 0.5195757

delta 0.3729298 0.4678955

##### Change-type (shift): only species (eng_name) included as random factor

Generalized linear mixed model fit by maximum likelihood (Laplace Approximation) ['glmerMod']

Family: binomial ( logit )

Formula: shift_type ~ H_type + H_breadth_sum + D_breadth_sum + D_type +

migration + IUCN + body_mass + clutch_size + lifespan + natal_dispersal +

thermal_max + thermal_range + hist_range + region + (1 | eng_name)

Data: EC_glmm

Control: glmerControl(optimizer = "bobyqa", optCtrl = list(maxfun = 1e+06))

AIC BIC logLik deviance df.resid

656.1 789.7 -298.0 596.1 606

Scaled residuals:

Min 1Q Median 3Q Max

-5.6707 -0.5377 0.2689 0.5733 4.5861

Random effects:

Groups Name Variance Std.Dev.

eng_name (Intercept) 0.1314 0.3625

Number of obs: 636, groups: eng_name, 244

Fixed effects:

Estimate Std. Error z value Pr(>|z|)

(Intercept) 1.680870 0.737244 2.280 0.022611 *

H_typeAgriculture_Grasslands -2.731837 0.845277 -3.232 0.001230 **

H_typeForest -1.145187 0.719628 -1.591 0.111529

H_typeInland wetlands -1.720086 0.711670 -2.417 0.015650 *

H_typeTundraMireMoor -3.299590 0.935374 -3.528 0.000419 ***

H_typeWood_Shrubland -1.637706 0.671014 -2.441 0.014661 *

H_typeOther -1.226080 0.977590 -1.254 0.209774

H_breadth_sum -0.227785 0.150183 -1.517 0.129338

D_breadth_sum -0.463666 0.213839 -2.168 0.030136 *

D_typeHerbi-invertivore -0.257502 0.703737 -0.366 0.714435

D_typeHerbivore 0.785699 0.535408 1.467 0.142246

D_typeInvertivore -0.006326 0.412462 -0.015 0.987763

D_typeOmnicarnivore 1.996339 0.866696 2.303 0.021257 *

D_typeOmnivore 1.171778 0.860274 1.362 0.173167

D_typePiscivore 0.894573 0.827888 1.081 0.279898

migrationMixed 0.570345 0.344187 1.657 0.097503 .

migrationShort-distance 0.518994 0.396627 1.309 0.190697

migrationLong-distance 0.002402 0.348764 0.007 0.994504

IUCNThreatened -1.446766 0.434488 -3.330 0.000869 ***

body_mass -0.286621 0.132430 -2.164 0.030440 *

clutch_size 0.104433 0.160033 0.653 0.514030

lifespan 0.393889 0.194217 2.028 0.042551 *

natal_dispersal 0.038626 0.179064 0.216 0.829215

thermal_max -0.169681 0.177078 -0.958 0.337949

thermal_range 0.038791 0.172832 0.224 0.822412

hist_range -0.128601 0.196677 -0.654 0.513199

regionFennoscandia 1.874480 0.344835 5.436 5.45e-08 ***

regionIberian peninsula 1.665640 0.479430 3.474 0.000512 ***

regionNorthwestern Europe -1.835201 0.315385 -5.819 5.92e-09 ***

---

R2m R2c

theoretical 0.4399665 0.4614761

delta 0.3701616 0.3882585
