# Supplementary material for: The Role of Intrinsic Factors in Explaining Range Shifts of European Breeding Birds: A Meta‐Analysis
Source: Ecol Evol. 2025 Apr 21;15(4):e71308. doi: 10.1002/ece3.71308 (PMC12012262; doi:10.1002/ece3.71308)
Supplement: Supplementary file 1 — Data S1. [file ECE3-15-e71308-s001.zip › Warmer et al_ecol evol_Appendices.docx]

# Appendix 1 – Methods, additional information

**Search queries:**

Web of Science

1. ALL=(distribut* OR occupancy OR biogeography OR “range shift” OR "range change" OR expansion OR contraction) AND ALL=(bird* OR avifauna) AND ALL=(europ* OR “western palearctic” OR palearctic OR Scandinavia OR Mediterranean OR British OR Baltic OR Balkan) NOT ALL=(genet*) NOT ALL=(infection OR immunity OR disease) NOT ALL=(sexual OR selection) AND pubyear > 1969

AND WC=(Ecology or Ornithology or Biodiversity Conservation or Environmental Sciences or Zoology or Multidisciplinary Sciences or Evolutionary Biology or Geography Physical or Biology or Marine Freshwater Biology or Behavioral Sciences or Forestry or Environmental Studies or Agriculture Multidisciplinary or Geography or Remote Sensing or Computer Science Theory Methods ) AND SU=(Environmental Sciences Ecology OR Zoology OR Biodiversity Conservation OR Science Technology Other Topics OR Physical Geography OR Forestry OR Agriculture)

1. (distribution OR range OR occupancy OR biogeography) AND (shift OR change OR size OR expansion OR contraction OR reduction OR northward OR latitudinal) AND (bird* OR avifauna)) NOT (mammal* OR carnivore OR fish OR insect) AND (europ* OR palearctic OR scandinavia* OR british OR baltic OR balkan OR mediterranean) NOT (asia OR china OR america OR canada) AND Ecology or Ornithology or Biodiversity Conservation or Environmental Sciences or Zoology or Multidisciplinary Sciences or Evolutionary Biology or Geography Physical or Biology or Behavioral Sciences (Web of Science Categories)

Scopus

1. ( TITLE-ABS-KEY ( distribut* OR occupancy OR biogeography OR "range shift" OR "range change" OR expansion OR contraction OR reduction ) AND TITLE-ABS-KEY ( bird* OR avifauna ) AND TITLE-ABS-KEY ( europ* OR "western palearctic" OR palearctic OR scandinavia OR mediterranean OR british OR baltic OR balkan ) AND NOT TITLE-ABS-KEY ( carnivore* OR mammal* OR insect ) AND NOT TITLE-ABS-KEY ( asia OR america ) AND NOT TITLE-ABS-KEY ( infection OR immunity OR disease ) AND NOT TITLE-ABS-KEY ( sexual OR selection ) AND NOT TITLE-ABS-KEY ( foraging ) ) AND PUBYEAR > 1969 AND ( LIMIT-TO ( SUBJAREA , "AGRI" ) OR LIMIT-TO ( SUBJAREA , "ENVI" ) OR LIMIT-TO ( SUBJAREA , "BIOC" ) OR LIMIT-TO ( SUBJAREA , "EART" ) OR LIMIT-TO ( SUBJAREA , "MULT" ) )

**
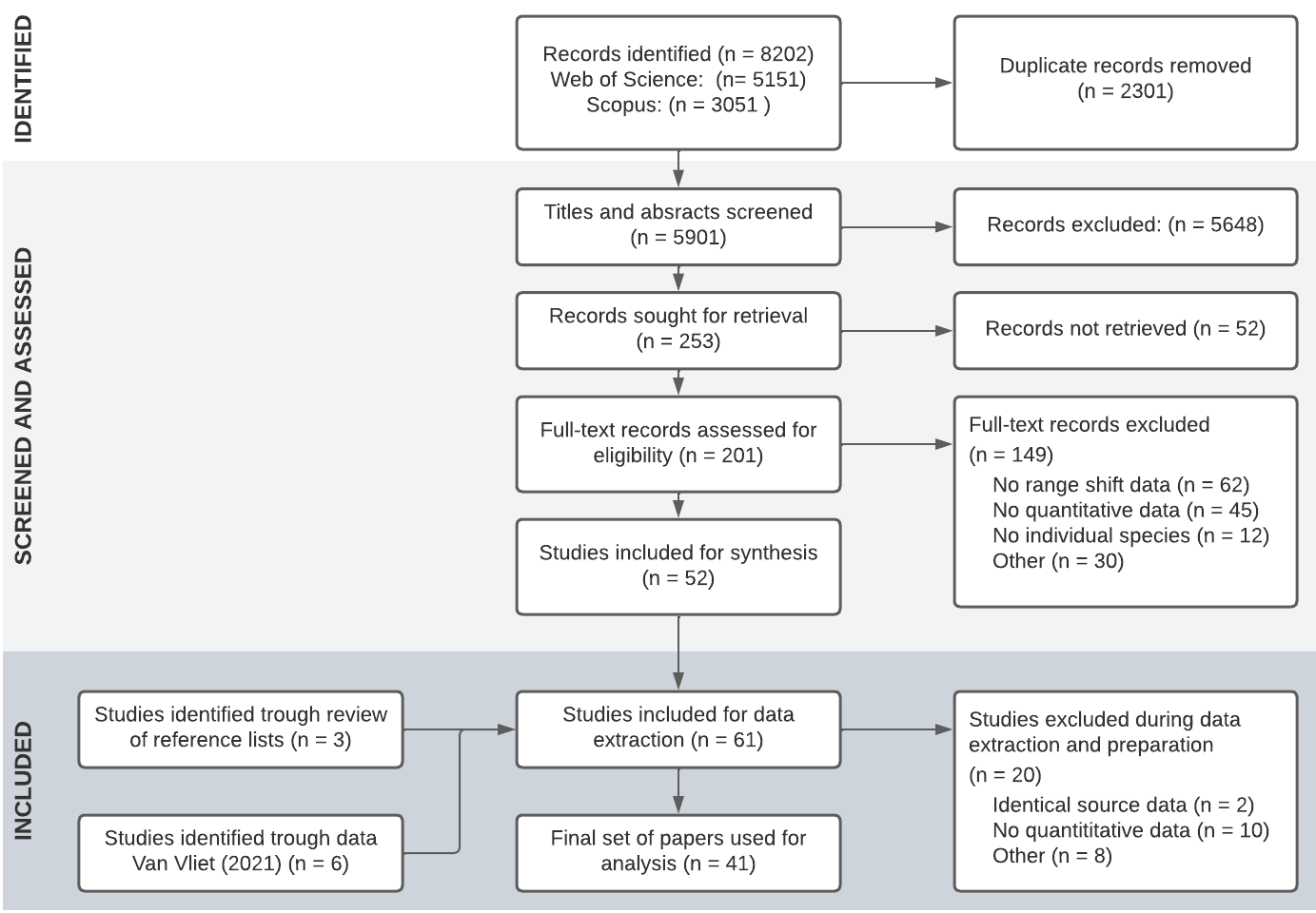
Structured literature review flow diagram, adapted from Page et al. (2021):**

# Appendix 2 – Source papers

**Reference list of the papers used for range shift data collection.**

Arroyo, B., Lafitte, J., Sourp, E., Rousseau, D., Albert, L., Heuacker, V., Terrasse, J. F., & Razin, M. (2021). Population expansion and breeding success of Bearded Vultures Gypaetus barbatus in the French Pyrenees: results from long-term population monitoring. Ibis, 163(1), 213-230. <https://doi.org/10.1111/ibi.12852>

Assandri, G., & Morganti, M. (2015). Is the Spectacled Warbler Sylvia conspicillata expanding northward because of climate warming? Bird Study, 62(1), 126-131. <https://doi.org/10.1080/00063657.2014.988600>

Banks, A. N., Crick, H. Q. P., Coombes, R., Benn, S., Ratcliffe, D. A., & Humphreys, E. M. (2010). The breeding status of Peregrine Falcons Falco peregrinus in the UK and Isle of Man in 2002. Bird Study, 57(4), 421-436. <https://doi.org/10.1080/00063657.2010.511148>

Böhning-Gaese, K., & Bauer, H. G. (1996). Changes in species abundance, distribution, and diversity in a central European bird community. Conservation Biology, 10(1), 175-187. <https://doi.org/10.1046/j.1523-1739.1996.10010175.x>

Brommer, J. E. (2004). The range margins of northern birds shift polewards. Annales Zoologici Fennici, 41(2), 391-397.

Brommer, J. E., Lehikoinen, A., & Valkama, J. (2012). The Breeding Ranges of Central European and Arctic Bird Species Move Poleward. PloS one, 7(9), 7, Article e43648. <https://doi.org/10.1371/journal.pone.0043648>

Brotons, L., Herrando, S., & Pons, P. (2008). Wildfires and the expansion of threatened farmland birds: the ortolan bunting Emberiza hortulana in Mediterranean landscapes. Journal of Applied Ecology, 45(4), 1059-1066. <https://doi.org/10.1111/j.1365-2664.2008.01467.x>

Carrillo, C., Barbosa, A., Valera, F., Barrientos, R., & Moreno, E. (2007). Northward expansion of a desert bird: effects of climate change? Ibis, 149(1), 166-169.

Conway, G., Wotton, S., Henderson, I., Eaton, M., Drewitt, A., & Spencer, J. (2009). The status of breeding Woodlarks Lullula arborea in Britain in 2006. Bird Study, 56, 310-325, Article Pii 913194031. <https://doi.org/10.1080/00063650902792163>

Conway, G., Wotton, S., Henderson, I., Langston, R., Drewitt, A., & Currie, F. (2007). Status and distribution of European Nightiars Caprimuigus europaeus in the UK in 2004. Bird Study, 54, 98-111. <https://doi.org/10.1080/00063650709461461>

Donald, P. F., & Greenwood, J. J. D. (2001). Spatial patterns of range contraction in British breeding birds. Ibis, 143(4), 593-601. <https://doi.org/10.1111/j.1474-919X.2001.tb04887.x>

Dougall, T. W., Holland, R. K., & Yalden, D. W. (2010). The population biology of Common Sandpipers in Britain. British Birds, 103(2), 100-114.

Engström, H. (2001). The occurrence of the Great Cormorant Phalacrocorax carbo in Sweden, with special emphasis on the recent population growth. Ornis Svecica, 11(3), 155-170.

Ferrer, X., Motis, A., & Peris, S. J. (1991). Changes in the breeding range of starlings in the Iberian Peninsula during the last 30 years: competition as a limiting factor. Journal of Biogeography, 18(6), 631-636. <https://doi.org/10.2307/2845544>

Fuller, R. J., Gregory, R. D., Gibbons, D. W., Marchant, J. H., Wilson, J. D., Baillie, S. R., & Carter, N. (1995). Population declines and range contractions among lowland farmland birds in Britain. Conservation Biology, 9(6), 1425-1441. <https://doi.org/10.1046/j.1523-1739.1995.09061425.x>

Fuller, R. J., Noble, D. G., Smith, K. W., & Vanhinsbergh, D. (2005). Recent declines in populations of woodland birds in Britain: A review of possible causes. British Birds, 98(3), 116-143.

Gil-Tena, A., Brotons, L., & Saura, S. (2009). Mediterranean forest dynamics and forest bird distribution changes in the late 20th century. Global change biology, 15(2), 474-485. <https://doi.org/10.1111/j.1365-2486.2008.01730.x>

Gregory, R. D., Wilkinson, N. I., Noble, D. G., Robinson, J. A., Brown, A. F., Hughes, J., Procter, D., Gibbons, D. W., & Galbraith, C. A. (2002). The population status of birds in the United Kingdom, Channel Islands and Isle of Man: An analysis of conservation concern 2002-2007. British Birds, 95(9), 410-448.

Henderson, I., Calladine, J., Massimino, D., Taylor, J. A., & Gillings, S. (2014). Evidence for contrasting causes of population change in two closely related, sympatric breeding species the Whinchat Saxicola rubetra and Stonechat Saxicola torquata in Britain. Bird Study, 61(4), 553-565. <https://doi.org/10.1080/00063657.2014.962482>

Henderson, I., Wilson, A., Steele, D., & Vickery, J. (2002). Population estimates, trends and habitat associations of breeding Lapwing Vanellus vanellus, Curlew Numenius arquata and Snipe Gallinago gallinago in Northern Ireland in 1999. Bird Study, 49(1), 17-25.

Heward, C. J., Hoodless, A. N., Conway, G. J., Aebischer, N. J., Gillings, S., & Fuller, R. J. (2015). Current status and recent trend of the Eurasian Woodcock Scolopax rusticola as a breeding bird in Britain. Bird Study, 62(4), 535-551. <https://doi.org/10.1080/00063657.2015.1092497>

Kolecek, J., & Reif, J. (2011). Differences between the predictors of abundance, trend and distribution as three measures of avian population change. Acta Ornithologica, 46(2), 143-153. <https://doi.org/10.3161/000164511x625919>

Lehikoinen, A., & Virkkala, R. (2016). North by north-west: climate change and directions of density shifts in birds. Global change biology, 22(3), 1121-1129. https://doi.org/10.1111/gcb.13150

Maclean, I. M. D., Austin, G. E., Rehfisch, M. M., Blew, J., Crowe, O., Delany, S., Devos, K., Deceuninck, B., Gunther, K., Laursen, K., Van Roomen, M., & Wahl, J. (2008). Climate change causes rapid changes in the distribution and site abundance of birds in winter. Global change biology, 14(11), 2489-2500. <https://doi.org/10.1111/j.1365-2486.2008.01666.x>

Marion, L., & Bergerot, B. (2018). Northern range shift may be due to increased competition induced by protection of species rather than to climate change alone [Article]. Ecology and Evolution, 8(16), 8364-8379. <https://doi.org/10.1002/ece3.4348>

Mason, S. C., Palmer, G., Fox, R., Gillings, S., Hill, J. K., Thomas, C. D., & Oliver, T. H. (2015). Geographical range margins of many taxonomic groups continue to shift polewards. Biological Journal of the Linnean Society, 115(3), 586-597. <https://doi.org/10.1111/bij.12574>

Pagel, J., Martinez-Abrain, A., Gomez, J. A., Jimenez, J., & Oro, D. (2014). A Long-Term Macroecological Analysis of the Recovery of a Waterbird Metacommunity after Site Protection. PloS one, 9(8), 13, Article e105202. <https://doi.org/10.1371/journal.pone.0105202>

Potvin, D. A., Välimäki, K., & Lehikoinen, A. (2016). Differences in shifts of wintering and breeding ranges lead to changing migration distances in European birds. Journal of Avian Biology, 47(5), 619-628. <https://doi.org/10.1111/jav.00941>

Purger, J. J. (2008). Numbers and distribution of red-footed falcons (Falco vespertinus) breeding in Voivodina (northern Serbia): A comparison between 1990-1991 and 2000-2001. Belgian Journal of Zoology, 138(1), 3-7.

Raine, A. F., Brown, A. F., Amano, T., & Sutherland, W. J. (2009). Assessing population changes from disparate data sources: the decline of the Twite Carduelis flavirostris in England. Bird Conservation International, 19(4), 401-416. <https://doi.org/10.1017/s0959270909990086>

Reif, J., St'astny, K., & Bejcek, V. (2010). Contrasting effects of climatic and habitat changes on birds with northern range limits in central Europe as revealed by an analysis of breeding bird distribution in the Czech Republic. Acta Ornithologica, 45(1), 83-90. <https://doi.org/10.3161/000164510x516128>

Stanbury, A., Davies, M., Grice, P., Gregory, R., & Wotton, S. (2010). The status of the Cirl Bunting in the UK in 2009. British Birds, 103(12), 702-711.

Tayleur, C., Caplat, P., Massimino, D., Johnston, A., Jonzen, N., Smith, H. G., & Lindstrom, A. (2015). Swedish birds are tracking temperature but not rainfall: evidence from a decade of abundance changes. Global Ecology and Biogeography, 24(7), 859-872. https://doi.org/10.1111/geb.12308

Taylor, K., Hudson, R., & Horne, G. (1988). Buzzard breeding distribution and abundance in britain and northern ireland in 1983. Bird Study, 35(2), 109-118. <https://doi.org/10.1080/00063658809480387>

Thorup, K., Sunde, P., Jacobsen, L. B., & Rahbek, C. (2010). Breeding season food limitation drives population decline of the Little Owl Athene noctua in Denmark. Ibis, 152(4), 803-814. <https://doi.org/10.1111/j.1474-919X.2010.01046.x>

Treinys, R., Dementavičius, D., Rumbutis, S., Švažas, S., Butkauskas, D., Sruoga, A., & Dagys, M. (2016). Settlement, habitat preference, reproduction, and genetic diversity in recovering the white-tailed eagle haliaeetus albicilla population. Journal of Ornithology, 157(1), 311-323. <https://doi.org/10.1007/s10336-015-1280-8>

Valera, F., Rey, P., Sanchez-Lafuente, A. M., & Muñoz-Cobo, J. (1993). Expansion of Penduline Tit (Remiz pendulinus) through migration and wintering. Journal Fur Ornithologie, 134(3), 273-282.

Välimäki, K., Linden, A., & Lehikoinen, A. (2016). Velocity of density shifts in Finnish landbird species depends on their migration ecology and body mass. Oecologia, 181(1), 313-321. <https://doi.org/10.1007/s00442-015-3525-x>

Van Rijn, S. (2018). Broedende Rode Wouwen in Nederland in 1976-2017. Limosa, 91(1), 3-15.

Velevski, M., Nikolov, S. C., Ben, H., Dobrev, V., Sidiropoulos, L., Saravia, V., Tsiakiris, R., Arkumarev, V., Galanaki, A., Kominos, T., Stara, K., Kret, E., Grubac, B., Lisicanec, E., Kastritis, T., Vavylis, D., Topi, M., Hoxha, B., & Oppel, S. (2015). Population decline and range contraction of the Egyptian Vulture Neophron percnopterus in the Balkan Peninsula. Bird Conservation International, 25(4), 440-450. <https://doi.org/10.1017/s0959270914000343>

Virkkala, R., Heikkinen, R. K., Lehikoinen, A., & Valkama, J. (2014). Matching trends between recent distributional changes of northern-boreal birds and species-climate model predictions. Biological Conservation, 172, 124-127. <https://doi.org/10.1016/j.biocon.2014.01.041>

Virkkala, R., & Lehikoinen, A. (2017). Birds on the move in the face of climate change: High species turnover in northern Europe. Ecology and Evolution, 7(20), 8201-8209. <https://doi.org/10.1002/ece3.3328>

Zielińska, M., Zieliński, P., Kołodziejczyk, P., Szewczyk, P., & Betleja, J. (2007). Expansion of the Mediterranean gull Larus melanocephalus in Poland. Journal of Ornithology, 148(4), 543-548.

# Appendix 3 – Supplementary tables

Supplementary file with all supplementary tables (S1-S7).

Filename: Warmer et al._Appx3.docx

# Appendix 4 – Model comparisons full models

Supplementary file with all full model comparisons.

Filename: Warmer et al._Appx4.docx

# Appendix 5 – Residual diagnostics

DHARMa residual diagnostics output for Change-type:


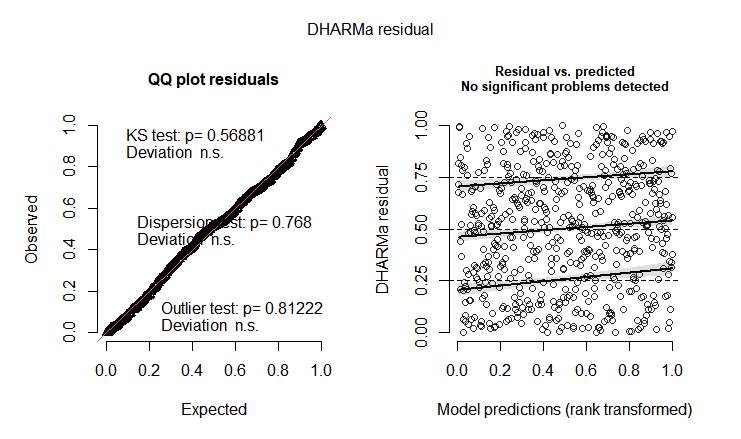


DHARMa residual diagnostics output for Relative-change before transformation:


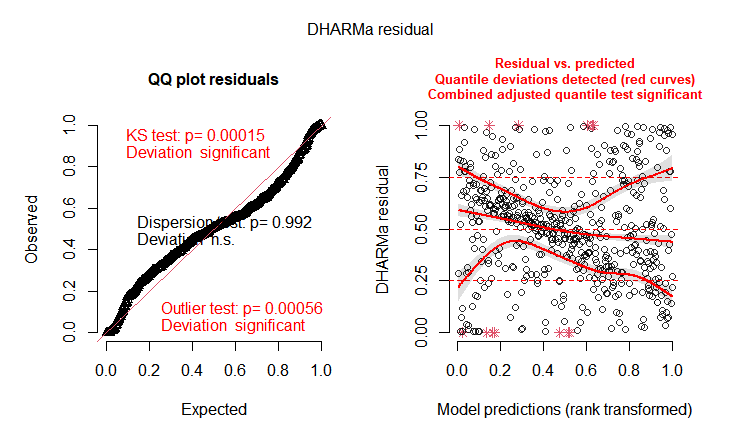
DHARMa residual diagnostics output for Relative-change

after log-modulus transformation:


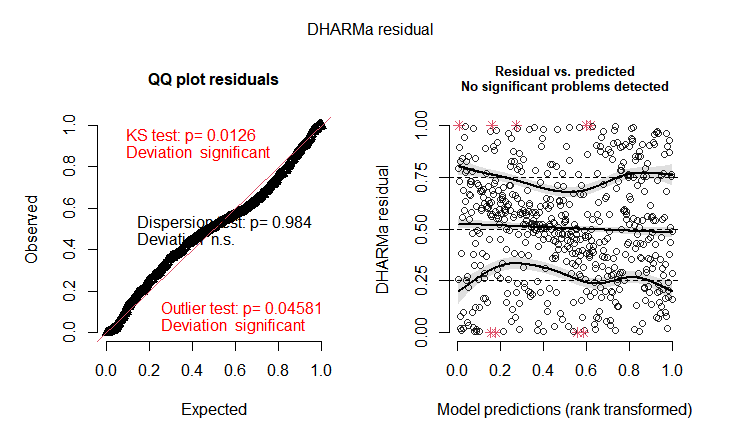


DHARMa residual diagnostics output for Rate-of-change before transformation:


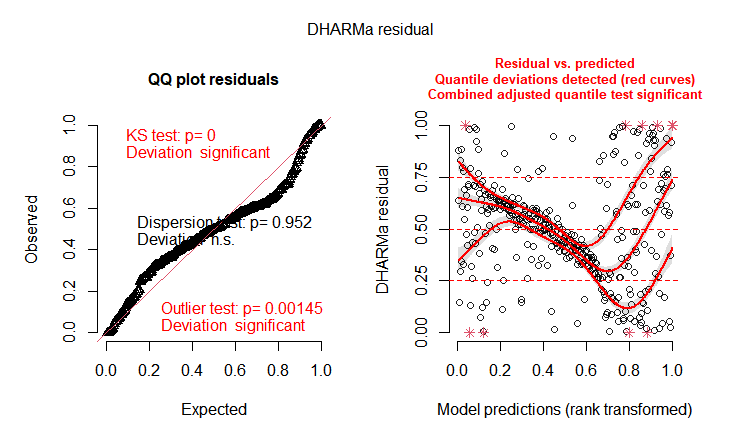


DHARMa residual diagnostics output for Rate-of-change after log-modulus transformation:


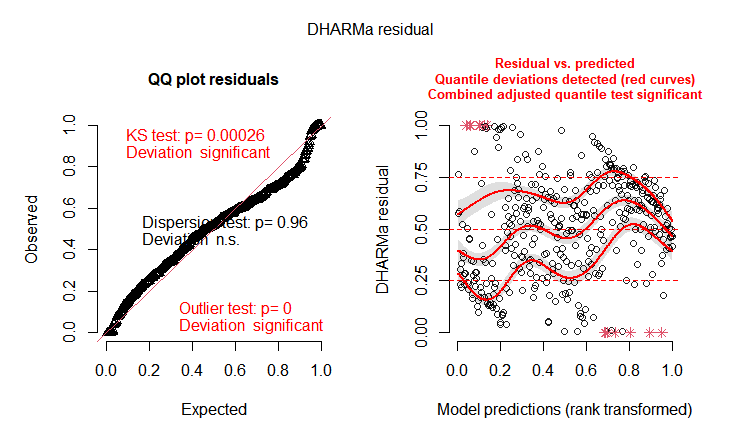


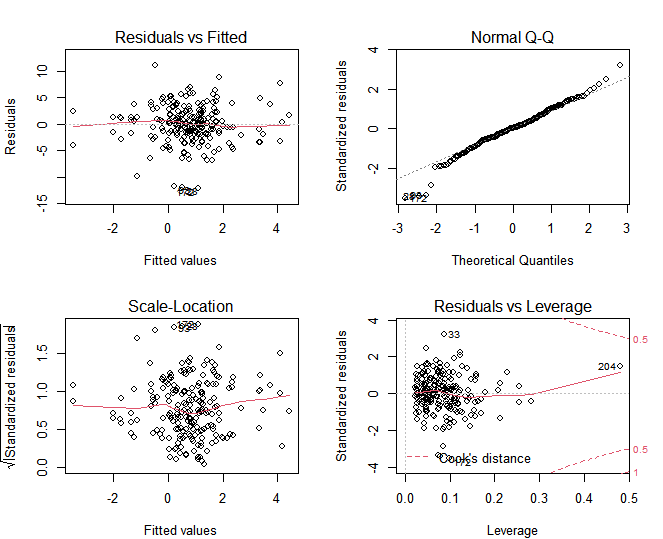
Residual diagnostics plots for Abundance shift:

Residual diagnostics plots for Centroid shift before transformation:


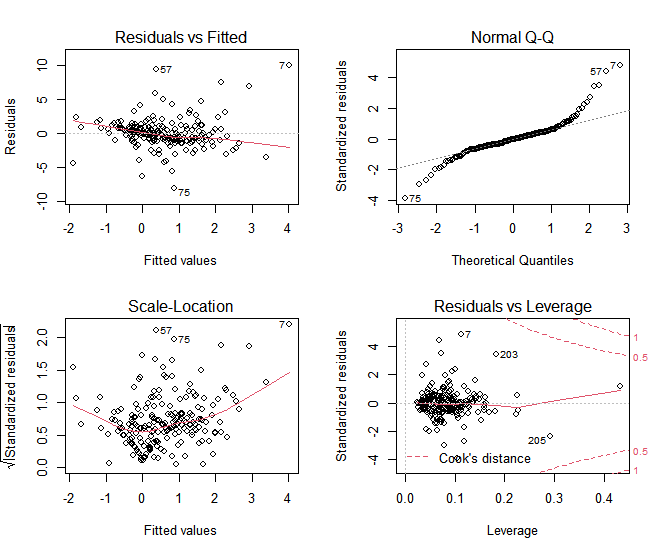

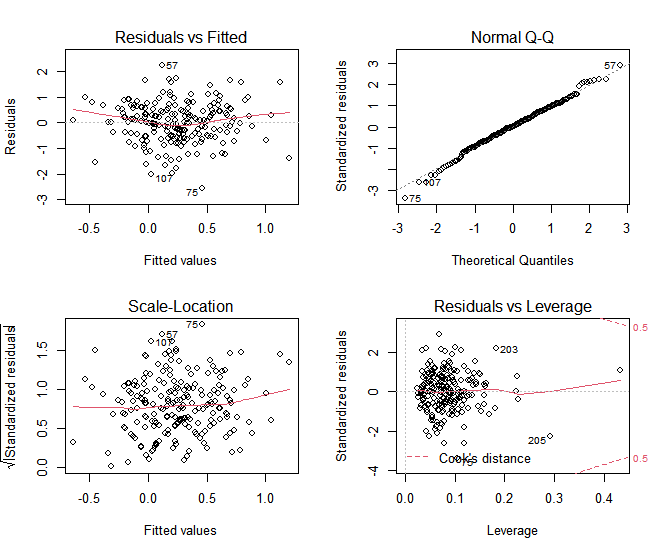


Residual diagnostics plots for Centroid shift after log-modulus transformation:


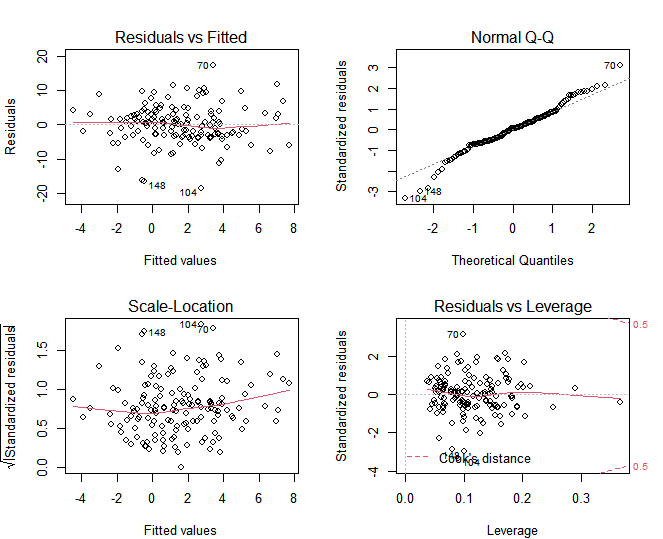


Residual diagnostics plots for

N-margin shift:

# Appendix 6 - Supplementary Data File

The accompanying Excel spreadsheet contains the raw data used in this study, including a sheet with the raw range shift datapoints for each range shift category including species traits, a sheet with a species list, and a sheet with the species trait data.

Filename: Warmer et al._Appx6.xlsx

# Appendix 7 - Supplementary Data File

The accompanying text file shows the R script for the calculation of the species thermal maximum, minimum and range, as well as the calculation of the historical range variables.

Filename: Appendix 7 in Dryad: Dataset DOI: [10.5061/dryad.wstqjq2z5](https://datadryad.org/submission/10.5061/dryad.wstqjq2z5)

# Appendix 8 - Supplementary Data File

The accompanying text file shows the R script for model selection and multi-model inference of the Range size change dataset, including the three range shift types Change-type, Relative-change, and Rate-of-change.

Filename: Appendix 8 in Dryad: Dataset DOI: [10.5061/dryad.wstqjq2z5](https://datadryad.org/submission/10.5061/dryad.wstqjq2z5)

# Appendix 9 - Supplementary Data File

The accompanying text file shows the R script for model selection and multi-model inference of the Abundance shift dataset.

Filename: Appendix 9 in Dryad: Dataset DOI: [10.5061/dryad.wstqjq2z5](https://datadryad.org/submission/10.5061/dryad.wstqjq2z5)

# Appendix 10 - Supplementary Data File

The accompanying text file shows the R script for model selection and multi-model inference of the Centroid shift dataset.

Filename: Appendix 6 in Dryad: Dataset DOI: [10.5061/dryad.wstqjq2z5](https://datadryad.org/submission/10.5061/dryad.wstqjq2z5)

# Appendix 11 - Supplementary Data File

The accompanying text file shows the R script for model selection and multi-model inference of the Northern range margin shift dataset.

Filename: Appendix 11 in Dryad: Dataset DOI: [10.5061/dryad.wstqjq2z5](https://datadryad.org/submission/10.5061/dryad.wstqjq2z5)

# Appendix 12 – Histogram plots


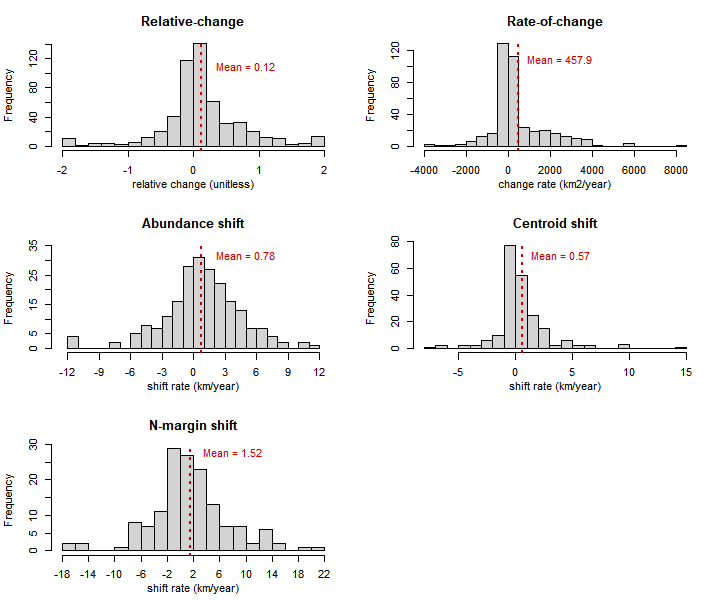
The figure below shows the histogram plots with indicated mean value of the range shift data for all range shift types except Change-type.
